# The WIRE study a phase II, multi-arm, multi-centre, non-randomised window-of-opportunity clinical trial platform using a Bayesian adaptive design for proof-of-mechanism of novel treatment strategies in operable renal cell cancer – a study protocol

**DOI:** 10.1186/s12885-021-08965-4

**Published:** 2021-11-18

**Authors:** Stephan Ursprung, Helen Mossop, Ferdia A. Gallagher, Evis Sala, Richard Skells, Jamal A. N. Sipple, Thomas J. Mitchell, Anita Chhabra, Kate Fife, Athena Matakidou, Gemma Young, Amanda Walker, Martin G. Thomas, Mireia Crispin Ortuzar, Mark Sullivan, Andrew Protheroe, Grenville Oades, Balaji Venugopal, Anne Y. Warren, John Stone, Tim Eisen, James Wason, Sarah J. Welsh, Grant D. Stewart

**Affiliations:** 1 CRUK Cambridge Centre, University of Cambridge, Cambridge, UK; 2 Population Health Sciences Institute, Newcastle University, Newcastle upon Tyne, UK; 3 Addenbrooke’s Hospital, Cambridge University Hospitals NHS Foundation Trust, Cambridge, UK; 4 AstraZeneca, Cambridge, UK; 5 Wellcome Sanger Institute, Hinxton, UK; 6 Oxford University Hospitals National Health Service Foundation Trust, Oxford, UK; 7 Department of Urology, Queen Elizabeth University Hospital, Glasgow, UK; 8 Institute of Cancer Sciences, University of Glasgow, Beatson West of Scotland Cancer Centre, Glasgow, UK; 9 Medical Research Council Biostatistics Unit, University of Cambridge, Cambridge, UK

**Keywords:** Clinical trial protocol [MeSH], Clear cell renal cell carcinoma [MeSH], Phase II clinical trial [MeSH], Bayesian adaptive trial, Olaparib [MeSH], Cediranib [MeSH], Durvalumab [MeSH], Window-of-opportunity, Neoadjuvant therapy [MeSH]

## Abstract

**Background:**

Window-of-opportunity trials, evaluating the engagement of drugs with their biological target in the time period between diagnosis and standard-of-care treatment, can help prioritise promising new systemic treatments for later-phase clinical trials. Renal cell carcinoma (RCC), the 7^th^ commonest solid cancer in the UK, exhibits targets for multiple new systemic anti-cancer agents including DNA damage response inhibitors, agents targeting vascular pathways and immune checkpoint inhibitors. Here we present the trial protocol for the WIndow-of-opportunity clinical trial platform for evaluation of novel treatment strategies in REnal cell cancer (WIRE).

**Methods:**

WIRE is a Phase II, multi-arm, multi-centre, non-randomised, proof-of-mechanism (single and combination investigational medicinal product [IMP]), platform trial using a Bayesian adaptive design. The Bayesian adaptive design leverages outcome information from initial participants during pre-specified interim analyses to determine and minimise the number of participants required to demonstrate efficacy or futility. Patients with biopsy-proven, surgically resectable, cT1b+, cN0–1, cM0–1 clear cell RCC and no contraindications to the IMPs are eligible to participate. Participants undergo diagnostic staging CT and renal mass biopsy followed by treatment in one of the treatment arms for at least 14 days. Initially, the trial includes five treatment arms with cediranib, cediranib + olaparib, olaparib, durvalumab and durvalumab + olaparib. Participants undergo a multiparametric MRI before and after treatment. Vascularised and de-vascularised tissue is collected at surgery. A ≥ 30% increase in CD8+ T-cells on immunohistochemistry between the screening and nephrectomy is the primary endpoint for durvalumab-containing arms. Meanwhile, a reduction in tumour vascular permeability measured by *K*^trans^ on dynamic contrast-enhanced MRI by ≥30% is the primary endpoint for other arms. Secondary outcomes include adverse events and tumour size change. Exploratory outcomes include biomarkers of drug mechanism and treatment effects in blood, urine, tissue and imaging.

**Discussion:**

WIRE is the first trial using a window-of-opportunity design to demonstrate pharmacological activity of novel single and combination treatments in RCC in the pre-surgical space. It will provide rationale for prioritising promising treatments for later phase trials and support the development of new biomarkers of treatment effect with its extensive translational agenda.

**Trial registration:**

ClinicalTrials.gov: NCT03741426 / EudraCT: 2018–003056-21.

**Supplementary Information:**

The online version contains supplementary material available at 10.1186/s12885-021-08965-4.

## Background

Renal cell carcinoma (RCC) is the 12^th^ commonest solid cancer globally (> 400,000 cases/year) and the 7^th^ commonest in the United Kingdom (~ 14,000 cases/year) [1]. Clear cell RCC (ccRCC), is the commonest histological subtype found in 75–80% of patients with RCC, and presents with metastatic disease in 16% of patients [2, 3].

Surgery is the treatment of choice in medically-fit patients whose disease burden is amenable to resection [4]. Consequently, 56% of patients undergo partial or radical resection of the tumour-bearing kidney as part of their treatment [5]. Currently guidelines recommend no peri-operative treatment in patients undergoing surgery with curative intent; however, 30% of patients will experience recurrence [6, 7]. For patients whose disease is not amenable to surgery, first-line treatment options for medically fit patients consist of immune-checkpoint inhibitors and/or vascular endothelial growth factor (VEGF) tyrosine kinase inhibitors (TKIs) [8, 9].

The increasing number of possible single-agent and combination therapeutic options makes it very challenging to prioritise agents in combination for further development in large randomised clinical trials. Therefore, novel trial designs, such as this window-of-opportunity trial assessing promising new combination treatments in the period of time between diagnosis and surgery, are required to determine if the combination results in the hypothesised mechanistic pharmacology [10]. A further advantage of the window-of-opportunity design allows assessing the biological activity of drugs in treatment naïve patients and ensures that translational analyses are not biased by previous treatments [10]. In the United Kingdom, Canada and the United States, patients often wait 1 month or more between the decision to proceed with nephrectomy and their surgery [11–13], making a window-of-opportunity trial design appropriate for the clinical pathway.

Genomic instability is a hallmark of cancer which chemo- and radiotherapy exploit while causing significant collateral damage to normal tissues [14]. More recently, targeted DNA damage response inhibitors (DDRi) and immune checkpoint inhibitors have taken advantage of tumour genomic instability and the resulting high mutational burden resulting in higher tumour specificity and efficacy in a range of different tumour types [15–17]. DDRi cause synthetic lethality in cells genetically lacking specific DNA repair mechanisms [18]. In contrast to this, contextual synthetic lethality occurs synergistically between a pharmacological DDRi and a second agent [19]. While ccRCC is not commonly associated with DDR deficiency, recent in-vitro and in-vivo data demonstrating replication stress as a consequence of common mutations associated with RCC (VHL loss: 85% of ccRCC, SETD2 mutations: 12% of ccRCC) and VEGF inhibition suggest that DDR-targeted treatment may be an effective and relevant therapeutic strategy [20, 21]. The initial arms of the WIndow-of-opportunity clinical trial platform for evaluation of novel treatment strategies in REnal cell cancer (WIRE) will evaluate the pharmacological activity of DDRi (Olaparib) alone and in combination with drug classes well established to be active in ccRCC including TKIs (cediranib) and immune checkpoint inhibitors (durvalumab), in patients with surgically resectable ccRCC.

Olaparib inhibits poly (ADP-ribose) polymerases (PARP 1–3) which are central to DNA damage repair and genomic stability. It inhibits the repair of single-strand DNA breaks specifically in cells deficient in homologous recombination, resulting in double strand-breaks, subsequent accumulation of mutations and cell death [22]. Olaparib has demonstrated improved progression-free survival (PFS) in placebo-controlled phase III studies in BRCA-mutated ovarian cancer [23, 24] and prolonged PFS in a phase III trial of patients with human epidermal growth factor receptor 2 (HER2)-negative breast cancer with a germline BRCA1/2 mutation compared to standard-of-care treatment [16]. In patients with metastatic prostate cancer and deleterious alterations in genes involved in homologous recombination repair, olaparib conferred prolonged overall survival (OS) and PFS compared to abiraterone or enzalutamide [25]. Meanwhile, cediranib, a potent VEGF-A and VEGFR-2 inhibitor causing a significant and rapid reduction in tumour blood-flow measured by dynamic contrast-enhanced magnetic resonance imaging (DCE-MRI), has demonstrated activity against ccRCC among other cancer types [26–28]. Cediranib in combination with olaparib improved PFS in ovarian cancer [29]. Given the replication stress induced by VEGF inhibition, the combination of cediranib + olaparib was prioritized as a potential therapeutic strategy for this platform trial [20, 21]. Finally, the immune checkpoint inhibitor durvalumab prolonged OS in patients with non-small cell lung cancer undergoing chemoradiotherapy in a placebo-controlled phase III trial [30]. The established efficacy of PD1/PD-L1 inhibitors in metastatic RCC [31, 32], coupled with increased anti-tumour activity of immune checkpoint inhibitors in combination with DDRi, owing to the increased expression of PD-L1 and activation of the stimulator of interferon genes (STING) pathway observed pre-clinically, rationalises this combination treatment [33, 34]. Here we present the trial protocol for the WIRE trial platform evaluating these novel treatment strategies in renal cancer and describe associated translational analyses.

## Methods/design

### Objectives

#### Primary objective

The WIRE trial aims to determine proof-of-mechanism for several agents (cediranib, olaparib and durvalumab) used as single or combination therapies by demonstrating engagement with the expected cellular pathways. Changes in capillary permeability will be determined in the cediranib, olaparib and cediranib + olaparib arms and changes to intra-tumoral CD8+ T-cell infiltration will be determined in the durvalumab and durvalumab + olaparib arms.

#### Secondary objectives

Secondary objectives will aim to investigate the safety of the investigational medicinal products (IMP) in the preoperative period and determine changes in primary tumour size and overall response measured according to the Response Evaluation Criteria in Solid Tumors (RECIST) version 1.1 [35]. Where they are not primary objectives, changes in intratumoral CD8+ T-cells and capillary permeability will be assessed as secondary objectives.

#### Exploratory objectives

Exploratory objectives will aim to define the molecular biological response to the IMPs for candidate markers of biological effects in blood, urine, and tissue samples. Additionally, the relationship of the primary endpoints with genetic mutations in related genes will be investigated. Finally, the ability of automated histological image analysis and hyperpolarised [1-^13^C] pyruvate MRI imaging to detect biological treatment response will be explored.

### Trial design and participants

WIRE is a Phase II, multi-arm, multi-centre, non-randomised, proof-of-mechanism (single and combination IMPs), platform trial using a Bayesian adaptive design (ClinicalTrials.gov Identifier: NCT03741426) with sample size chosen to control frequentist statistical characteristics. It will be conducted at academic medical centres in the United Kingdom.

**Fig. 1:**
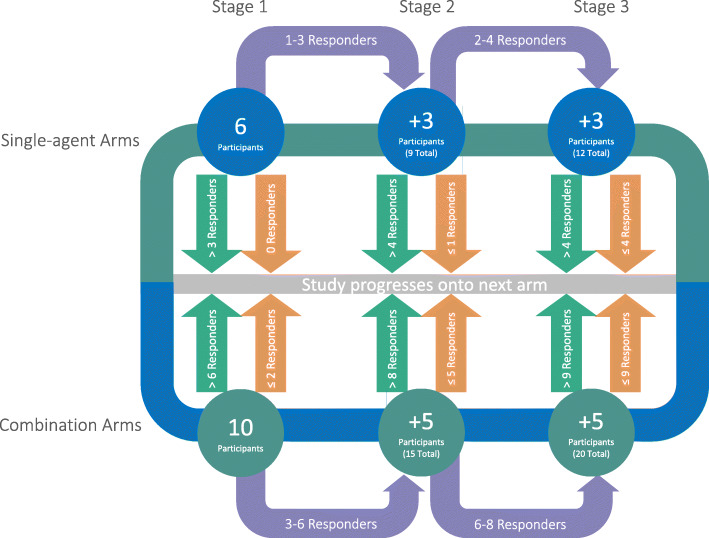
Bayesian adaptive trial design with interim analyses and decision boundaries for single-agent and combination-therapy arms. Green arrows denote efficacy, orange arrows denote futility

The Bayesian adaptive design leverages outcome information from initial participants to determine the recruitment target for each treatment arm (see Fig. 1). After reaching the recruitment target of six participants into single agent and ten participants into combination treatment arms in the initial stage, recruitment will continue into subsequent arms until the interim analysis is complete. Depending on the number of patients achieving the primary endpoint during these pre-planned interim analyses, the independent data monitoring committee (IDMC) may close the arm for reaching statistical efficacy or futility. If recruiting additional participants is likely to confer statistical efficacy, additional patients will be recruited into an arm until the target for the second interim analysis is reached. Each arm may proceed through up to three recruitment stages.

Potential participants will be identified during multi-disciplinary team case discussions and invited to participate in the trial during their standard clinical care visit. Participants will be provisionally assigned to the currently recruiting arm and provided a screening identifier. Upon successfully completing screening, participants will be allocated a trial identifier and continue with the arm allocated at screening. Participants may not cross to another arm or enter a reserve pool if they are ineligible for the current arm.

#### Eligibility criteria

Key inclusion criteria include age ≥ 18 years, biopsy-proven, surgically resectable, cT1b+, cN0–1, cM0–1 ccRCC, Eastern Cooperative Oncology Group (ECOG) performance status of 0 or 1, at least one measurable lesion according to RECIST 1.1, adequate organ and bone marrow function and contraception. Patients may not have been exposed to any PARP, tyrosine kinase, immune checkpoint or mammalian target of rapamycin (mTOR) inhibitors.

Key exclusion criteria include cT1a, N0, M0 ccRCC, brain metastasis, spinal cord compression unless definitely treated and non-progressive for 28 days and leptomeningeal involvement. Furthermore, patients with a bodyweight ≤30 kg, contraindication for any of the trial compounds, other invasive malignancy within the last 2 years, concurrent participation in another interventional clinical trial, cardio- or cerebrovascular complications within the last 12 months, immunosuppression or active infection, pregnancy or contraindications for MRI are excluded from participation. Women of childbearing potential must use two highly effective forms of contraception from consent until 3 months after the end of the trial.

Detailed reference values for adequate organ and bone marrow function as well as in- and exclusion criteria specific to individual treatment arms can be found in the Additional file 2.

#### Informed consent

A trial entrant will provide written informed consent before any trial-specific intervention in accordance with the principles of the International Council for Harmonisation of Technical Requirements for Pharmaceuticals for Human Use – Good Clinical Practice (IHC-GCP) [36]. Translation of the patient information and consent form will be provided if necessary and trial entrants will have at least 24 h to consider the patient information materials. Additional consent for the collection of blood, urine and tissue samples as well as genetic analyses will be sought. Consent documents are available in the Additional file 3.

### Interventions

#### Intervention description

This trial includes five sequential treatment arms: 1) cediranib; 2) cediranib + olaparib; 3) olaparib; 4) durvalumab and 5) olaparib + durvalumab. IMPs for the individual arms are summarized in Table 1.

**Table 1:** Treatment Arms in the WIRE trial. bd: twice daily, IMP: Investigational Medicinal Product, i.v.: intravenous, od: once daily. Dose reductions permissible in case of toxicity

Arm	IMP	Dosing, Stopping and Dose Adjustments
1	Cediranib	20 mg oral od, until 36 h before surgery, single reduction to 15 mg od
2	Cediranib + Olaparib	Cediranib 20 mg oral od and Olaparib 300 mg oral bd, stopping and dose adjustments as in single-agent arms
3	Olaparib	300 mg oral bd, until morning of surgery, two reductions to 250 mg bd and 200 mg bd
4	Durvalumab	1500 mg i.v. (single infusion)
5	Olaparib + Durvalumab	Olaparib 300 mg oral bd and Durvalumab 1500 mg i.v. (single infusion). Stopping and dose adjustment for Olaparib as in single-agent arm above.

*bd* twice daily, *od* once daily, *i.v*. intravenous

Regardless of the treatment arm, patients will undergo nephrectomy between 17 and 28 days after the first day of IMP (Fig. 1).

#### Criteria for discontinuing or modifying allocated interventions

In the case of toxicity, oral IMPs may be interrupted and can be dose-reduced as detailed in Table 1. If the toxicity persists, the IMP should be discontinued. In the combination arms, the doses of cediranib and olaparib will both be reduced unless the toxicity can be attributed to the toxicity profile of one of the IMPs. In case of discontinuation, both IMPs will be discontinued.

Participants will have their IMP discontinued if they experience unacceptable toxicity despite dose reductions, disease progression per RECIST 1.1, pregnancy, weight loss to ≤30 kg or withdraw consent. Participants receiving olaparib will have their IMP discontinued if they develop myelodysplastic syndrome or acute myeloid leukemia.

#### Relevant concomitant care permitted or prohibited during the trial

All routinely used supportive care will be allowed during WIRE, except for certain medications prohibited and restricted for safety or efficacy reasons. These are detailed in the Additional file 2.

#### Participant timeline

**Fig. 2:**
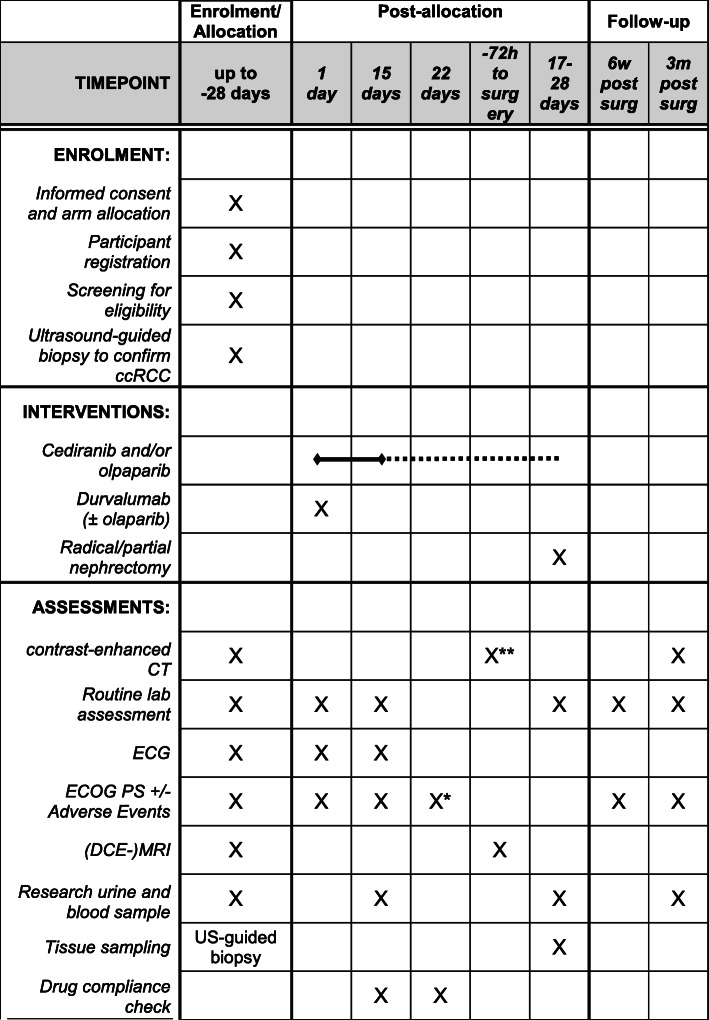
Timeline of interventions and assessments for the WIRE trial. *Optional, **Only in patients with cM1 disease. US: ultrasound

Figure 2 summarises the participant timelines for all arms.

### Outcomes

#### Primary outcomes

Reduction in tumour vascular permeability by > 30% measured by median *K*^trans^ on DCE-MRI between the baseline and pre-surgical MRI will be the primary endpoint for the cediranib, olaparib and cediranib + olaparib treatment arms. *K*^trans^ is a sensitive and early marker of response to VEGFR TKI in renal cancer and to olaparib in ovarian cancer [37, 38]. Additionally, pre-clinical research has demonstrated that the impaired vascular endothelial cell migration resulting from PARP inhibition has an anti-angiogenic effect [39]. The primary endpoint for the durvalumab containing arms will be an increase in CD8+ T-cells on immunohistochemistry (IHC) assessed by histoscore between the baseline biopsy and samples taken at nephrectomy. The rationale for this endpoint is based on evidence in melanoma that an increased number of tumour infiltrating CD8+ T-cells is associated with a better response to immune checkpoint inhibitors [40, 41].

#### Secondary outcomes

- Adverse events assessed according to the Common Terminology Criteria for Adverse Events (CTCAE) Toxicity Criteria (Version 5.0)
- Tumour size change between pre- and post-treatment MRI
- Tumour response according to RECIST v1.1 on pre- and post-treatment MRI in participants with M0 disease and pre-and post-treatment contrast-enhanced CT in participants with M1 disease
- Changes in *K*^trans^ and tumour infiltrating CD8+ T-cells where these are not primary outcomes

#### Exploratory outcomes

Exploratory outcomes will include the assessment of blood, urine, tissue and imaging-based biomarkers of drug mechanism and treatment response.

### Data collection and management

#### Assessment and collection of outcomes

Primary endpoints will be assessed centrally for all trial sites when the number of participants required for an interim or final analysis has been reached.

All pseudo-anonymised imaging data will be transferred to the trial radiologist for the measurement of the tissue transfer constant *K*^trans^ on DCE-MRI. Following motion correction, the MiStar software (Apollo Medical, Melbourne, Australia) will be used for kinetic modelling of the contrast-enhancement data using the extended Tofts model [42] and a model arterial input function [43]. Regions of interest encompassing the entire tumour will be defined and independently reviewed by two radiologists. Two radiologists verify the correct recording of the median *K*^trans^ value independently.

The CD8+ T-cell count will be established centrally following transfer of the formalin-fixed paraffin-embedded tissue blocks. Following slicing of the block, endogenous peroxidase suppression and heat-mediated antigen retrieval, the mouse monoclonal anti-human CD8 (DAKO Cytomation, Glostrup, Denmark, 1:100 dilution) will be the primary antibody. Slides will be scanned and two board-certified uro-pathologists will quantify the CD8+ T-cells in ten random fields of view per tumour sample, expressed as the median number of cells/mm^2^ before and after treatment.

#### Participant replacement

Participants will be replaced and not evaluated for the primary endpoint if they have not received at least 14 days of IMP and/or a single infusion of durvalumab, had to interrupt the IMP for > 7 consecutive days, had a compliance rate of < 70% in the 7 days preceding the pre-surgical imaging, had no pre-surgical imaging or had surgery more than 28 days after the first day of IMP.

#### Data management and confidentiality

All trial-related information will be stored securely at the trial site and transferred pseudo-anonymised through an encrypted electronic case report form (eCRF). The eCRF has inbuilt mechanisms to validate the integrity of data entries. Data will be checked for clarity, errors and completeness ahead of data export by two independent staff members. A pseudo-anonymised copy of imaging data used to obtain the primary endpoint will be retained centrally.

#### Collection, evaluation and storage of biological specimens and translational analyses

During screening, patients will undergo urine and blood collection followed by ultrasound-guided, multi-regional biopsy of the renal tumour. The biopsy procedure will be carried out by a consultant interventional radiologist. Additional blood and urine samples will be collected after 14 days of IMP and immediately before surgery. At surgery, samples will be collected from the vascularized (i.e. during the nephrectomy) and devascularised (i.e. ex vivo) tumour. 3D-printed, patient-specific tumour moulds will be used to collect devascularised samples from locations spatially registered to pre-surgical imaging at one of the trial sites. If lesions have a major axis > 20 cm, biopsies will be taken without the mould. All trial samples will be stored and analysed in a pseudo-anonymised manner.

The translational analysis aims to explore potential future endpoint markers and determine the response of molecular markers in DDR-, immune-, apoptosis- and cell-cycle pathways.

DNA and RNA analyses in tissue, blood and urine will attempt to explore signatures of response and resistance to IMPs and confirm the IMP’s mechanism of action. Additionally, intratumor genetic heterogeneity will be assessed from multi-regional tumour biopsies and compared to the genetic landscape observed in cell-free tumour DNA. Furthermore, WIRE aims to investigate the applicability of the NEAT (N-cadherin, EPCAM, Age, mTOR) proteomic score [44], which was developed for survival prediction in patients receiving sunitinib, in the IMPs under investigation, including the TKI cediranib. WIRE can explore immune- and cytokine-based signatures in peripheral blood mononucleocytes (PBMCs), plasma or urine which may predict response, resistance, or toxicity. These will be complemented by metabolomics analyses using mass-spectrometry and nuclear magnetic resonance on matched biopsy and surgical tumour tissue pairs, plasma, and urine. Finally, translational research within WIRE aims to address the unmet need for new in-vivo and in-vitro models of RCC, generating reproducible patient-derived xenograft (PDX) and cell line models to support the rational design of future therapies.

### Statistical methods

#### Sample size and power

Up to a maximum of 76 evaluable participants will be recruited into the initial five arms and across all stages.

##### Monotherapy arms (Arm 1 [Cediranib], Arm 3 [Olaparib], Arm 4 [Durvalumab])

Each monotherapy arm will enrol a maximum of 12 evaluable participants with interim analyses after 6 and 9 evaluable participants have primary outcome data available.

The null hypothesis is 20% or less of participants show a response on the primary outcome. The alternative hypothesis is that more than 20% of participants show a response:H_0_: *p* ≤ 0.2 H_1_: *p* > 0.2with the power calculated as the probability of rejecting H_0_ when *p* = 0.5.

The IMP will be recommended as promising (null hypothesis rejected) if the arm stops after stage 1 or 2 for efficacy (see Interim Analyses). If the IMP continues to stage 3, a *p*-value will be found by calculating the tail probability of seeing that many or more responses if the distribution were binomial with *p* = 0.2. The treatment will be recommended as promising if the p-value is ≤0.1.

The overall type I error rate for monotherapy arms is 20%; the overall power is 92% when *p* = 0.5. The ‘overall type I error rate’ for an arm is defined as the probability of an inefficacious arm (*p* < =0.2) having its null hypothesis rejected. This is the sum of the probabilities of meeting an efficacy stopping rule after stage 1 or 2 and reaching the final analysis and having a final p-value of ≤0.1. The overall power is this sum of probabilities when the arm is efficacious (*p* = 0.5).

Although 20% type I error rate is higher than traditional in phase II, each monotherapy is tested again within the combination IMP arms.

$$ \mathrm{Combination}\ \mathrm{IMP}\ \mathrm{arms}\ \left(\mathrm{Arm}\ 2\ \left[\mathrm{Cediranib}+\mathrm{Olaparib}\right],\mathrm{Arm}\ 5\left[\mathrm{Olaparib}+\mathrm{Durvalumab}\right]\right) $$

##### Each combination arm will enrol a maximum of 20 evaluable participants with interim analyses after 10 and 15 evaluable participants have primary outcome data available

The null hypothesis is 30% or less of participants show a response on the primary outcome. The alternative hypothesis is that more than 30% of participants show a response:

- H0: *p* ≤ 0.3
- H1: *p* > 0.3

with the power calculated as the probability of rejecting H0 when *p* = 0.6.

The combination will be recommended as promising (null hypothesis rejected) if the arm stops after stage 1 or 2 for efficacy (see Interim Analyses). If the combination continues to stage 3, a *p*-value will be found by calculating the tail probability of seeing that many or more responses if the distribution were binomial with *p* = 0.3. The IMP will be recommended as promising if the p-value is ≤0.05.

The overall type I error rate for combination IMP arms is 10%; the overall power is 93% when p = 0.6.

Simulation code in R to recreate the statistical properties is provided as Additional file 1.

#### Interim analyses

Interim data analyses at predefined points will decide whether the recruitment of additional patients into an arm should occur. The Bayesian analysis will use a Beta (0.3, 0.7) prior for the biological response rate (the p parameter). Through updating this prior with the observed number of biological responders using a Beta-Binomial conjugate distribution we will calculate a Beta posterior distribution p.

For monotherapy arms, if the calculated posterior probability of the biological response rate being above 0.2 is above 98%, the arm will be closed early for efficacy. If the predictive probability that the IMP would be found to be promising (i.e. the final one-sided *p*-value would be ≤0.1) if the full enrolment of 12 was reached is < 2%, then the arm will be closed early for futility. This use of the posterior distribution for efficacy stopping and predictive distribution for futility stopping is as recommended by Berry et al. [45].

For combination IMP arms, if the calculated posterior probability of the biological response rate being above 0.3 is above 98%, the arm will be closed early for efficacy. If the predictive probability that the IMP would be found to be promising (i.e. the final one-sided p-value would be ≤0.05) if the full enrolment of 20 was reached is < 2%, then the arm will be closed early for futility.

The final p-value thresholds are chosen to control the overall type I error rate at 20% for monotherapy arms and 10% for combination arms.

We note that the efficacy stopping rule is provided as a recommendation to the IDMC (see Additional file 2*)*. The IDMC may decide that continuing to recruit participants to an arm that meets the efficacy stopping rules is warranted. If this occurs, then the type I error rate and power will both be lower than the aforementioned levels.

The recruitment targets for the interim analyses are summarised in Fig. 1. No subgroup analysis beyond the treatment arms is planned for this trial.

#### Statistical methods for primary and secondary outcomes and missing data

A Statistical Analysis Plan (SAP) will define appropriate 95% two-sided Confidence Intervals for primary and secondary endpoint quantities prior to the first interim analysis. The extent of missing data will be reported however no imputation of missing data is planned.

#### Plans to give access to the full protocol, participant level-data and statistical code

The full protocol and participant level-data will not be made available. A more detailed description of the development of the Bayesian adaptive statistics used in the design of this trial will be available in an independent publication. Statistical code will be made available upon reasonable request.

### Oversight and monitoring

#### Adverse event reporting and harms

Adverse events will be monitored and recorded from the time of consent until 90 days after the last administration of IMP. Any adverse event will be recorded in the patient notes and the eCRF. The principal investigators will report serious adverse events (SAEs) to the chief investigator within 24 h of awareness. The chief investigator will subsequently assess SAEs for expectedness and relatedness prior to onwards notification to the sponsor within a further 24 h. The chief investigator will additionally report SAEs to AstraZeneca within 24 h. The CI must report suspected unexpected serious adverse reactions (SUSARs) to the Medicines and Healthcare products Regulatory Agency (MHRA) and the ethics committee within 7 to 15 days depending on the severity. Reporting of SAEs which are attributable to any of the IMPs (i.e. serious adverse reactions) and SUSARs continues beyond the 90-day monitoring period and after withdrawal of consent by the participant, where investigators become aware of any new events.

More details on trial oversight are available in the Additional file 2.

### Dissemination plans

The clinical and exploratory endpoints are anticipated to be of great interest to the RCC research community due to the novel combinations being used. The results will be reported at major oncology conferences and then published in high-impact journals. Patient facing publications will be made available to interested trial participants and via our website.

## Discussion

Here we present an adaptive and expandable phase II clinical trial platform to demonstrate proof-of-mechanism for single-agent and combination treatments in ccRCC.

The Bayesian statistics-based adaptive trial design optimises the number of trial participants in each arm, ensuring that no more participants than necessary are recruited to demonstrate proof of mechanism or futility of any arm [45]. This accelerates the screening of agents and combinations. Also, the Bayesian framework allows adjusting for the uncertainty arising from the unknown variability of emerging endpoints in the context of new treatments. In addition to prioritising promising treatment arms for later phase clinical trials, the extensive liquid and tumour bio-sampling and use of advanced imaging support the identification of new biomarkers of response, which are an area of unmet clinical need in RCC. The platform design of the trial also allows learning from and defining new endpoints based on accumulating study data. We have previously shown the benefit of using imaging biomarkers as part of an adaptive trial design protocol in the assessment of patients with RCC in the metastatic setting [46].

The window-of-opportunity design of this trial exposes patients to the trial medication for a brief period, prior to standard-of-care nephrectomy. While this will allow measuring the engagement of IMPs with their biological target, this trial is neither designed nor powered to accrue evidence of long-term treatment benefit, although there may be unmeasurable survival advantages for patients. Therefore, larger studies in patients who are not surgical candidates will be required to investigate survival benefits of promising treatment arms. Furthermore, the brief treatment may lead to an underestimation of the treatment effects.

A limitation inherent to the window-of-opportunity design is the risk of delaying standard-of-care treatment when serious adverse reactions occur. This risk is mitigated by the strict inclusion criteria, close oncology supervision to pre-empt and identify early any significant toxicity events, and the Bayesian adaptive trial design which leverages information gained during interim analyses to reduce the number of participants compared to conventional trial designs. This is also an advantage compared to other, slowly accruing window-of-opportunity trials. The risk of delaying surgery is discussed with potential participants in detail. On the other hand, an advantage of the window-of-opportunity design is that it increases the possibility to detect biological treatment effects quickly and in a small number of patients. Specifically, examining previously untreated patients who will undergo standard-of-care nephrectomy allows the investigation of IMPs in the absence of mutations induced by previous treatment(s) as well as access to tissue at surgery for extensive translational analyses.

This is the first clinical trial we are aware of using this pre-surgical window-of-opportunity to investigate novel agents and combination therapies in RCC with a Bayesian design. In the future, additional treatment arms may be added to this trial. In addition to studying new systemic anti-cancer agents, synergistic effects between radiation therapy localised to the tumour and DDR inhibition could be investigated.

In summary, WIRE will provide an important impetus for future phase II/III clinical trials to prioritise treatment regimens that show biological efficacy. Additionally, it will be able to inform the optimal choice of treatment response biomarkers.

### Trial status

Version 3.0 of the trial protocol (05/12/2019) has received ethical approval and is in date as of publication of this trial protocol. Recruitment for WIRE commenced on the 12/08/2020 and is estimated to be completed in 12/2022.

## Supplementary Information

**Additional file 1.**

**Additional file 2.**

**Additional file 3.**

## Data Availability

Not applicable.

## Abbreviations

ccRCC: Clear cell Renal Cell Carcinoma
DCE-MRI: Dynamic Contrast Enhanced Magnetic Resonance Imaging
DDRi: DNA Damage Response inhibitor
eCRF: Electronic Case Report Form
ECOG: Eastern Cooperative Oncology Group
IDMC: Independent Data Monitoring Committee
IHC: Immuno Histochemistry
IMP: Investigational Medicinal Product
OS: Overall survival
PARP: Poly (ADP-Ribose) Polymerase
PFS: Progression-Free Survival
RECIST: Recponse Criteria in Solid Tumours
SAE: Serious Adverse Event
SUSAR: Suspected Unexpected Serious Adverse Reaction
TKI: Tyrosine Kinase Inhibitor
VEGF(R): Vascular Endothelial Growth Factor (Receptor)
