# Supplementary material for: The WIRE study a phase II, multi-arm, multi-centre, non-randomised window-of-opportunity clinical trial platform using a Bayesian adaptive design for proof-of-mechanism of novel treatment strategies in operable renal cell cancer – a study protocol
Source: BMC Cancer. 2021 Nov 18;21:1238. doi: 10.1186/s12885-021-08965-4 (PMC8600815; doi:10.1186/s12885-021-08965-4)
Supplement: Supplementary file 2 — Additional file 2. [file 12885_2021_8965_MOESM2_ESM.docx]

The WIRE study a phase II, multi-arm, multi-centre, non-randomised window-of-opportunity clinical trial platform using a Bayesian adaptive design for proof-of-mechanism of novel treatment strategies in operable renal cell cancer

Supplementary Materials

Stephan Ursprung^1^, Helen Mossop^2^, Ferdia A Gallagher^1,3^, Evis Sala^1,3^, Richard Skells^3,8^, Jamal A. N. Sipple^3^, Thomas J Mitchell^1,3,4^, Anita Chhabra^3^, Kate Fife^3^, Athena Matakidou^3^, Gemma Young^3^, Amanda Walker^3^, Martin G Thomas^3^, Mireia Crispin Ortuzar^1^, Mark Sullivan^5^, Andrew Protheroe^5^, Grenville Oades^6^, Balaji Venugopal^7^, Anne Y Warren^1,3^, John Stone^8^, Tim Eisen^1,3^, James Wason^3,9^, Sarah J Welsh^1,3^, Grant D Stewart^1,3,^*

* Corresponding author

Affiliations:

1 CRUK Cambridge Centre, University of Cambridge, Cambridge, UK

2 Population Health Sciences Institute, Newcastle University, Newcastle upon Tyne, UK

3 Addenbrooke’s Hospital, Cambridge University Hospitals NHS Foundation Trust, Cambridge, UK

4 Wellcome Sanger Institute, Hinxton, UK

5 Oxford University Hospitals National Health Service Foundation Trust, Oxford, UK

6 Department of Urology, Queen Elizabeth University Hospital, Glasgow, UK

7 Institute of Cancer Sciences, University of Glasgow, Beatson West of Scotland Cancer Centre, Glasgow, UK

8 AstraZeneca, Cambridge, UK

9 Medical Research Council Biostatistics Unit, University of Cambridge, Cambridge, UK

Contents

[WHO Trial Registration Data Set 3](#_Toc64480614)

[Inclusion and Exclusion Criteria 4](#_Toc64480615)

[Inclusion Criteria 4](#_Toc64480616)

[Exclusion Criteria 5](#_Toc64480617)

[Relevant concomitant care permitted or prohibited during the trial 9](#_Toc64480618)

[Strategies to improve adherence to interventions 10](#_Toc64480619)

[Participant retention 10](#_Toc64480620)

[Provisions for post-trial care 10](#_Toc64480621)

[Oversight and Monitoring 10](#_Toc64480622)

[Composition of the coordinating centre and trial steering committee 10](#_Toc64480623)

[Composition of the data monitoring committee, its role and reporting structure 10](#_Toc64480624)

[Frequency and plans for auditing trial conduct 11](#_Toc64480625)

[Plans for communicating important protocol amendments to relevant parties 11](#_Toc64480626)

# WHO Trial Registration Data Set

1. **Primary Registry and Trial Identifying Number**
   ClinicalTrials.gov: NCT03741426
2. **Date of Registration in Primary Registry**
   14^th^ November 2018
3. **Secondary Identifying Numbers**
   EudraCT: 2018-003056-21
   Sponsor: WIRE
4. **Source(s) of Monetary or Material Support**
   The trial will be part funded by AstraZeneca UK and Cancer Research UK. Olaparib, cediranib and durvalumab will be supplied free of charge by AstraZeneca UK.
5. **Primary Sponsor**
   The trial is sponsored by Cambridge University Hospitals NHS Foundation Trust (CUH) and University of Cambridge (UoC).
6. **Contact for Public Queries**
   Cambridge University Hospitals NHS Foundation Trust, Research and Development Department, Box 277, Cambridge Biomedical Campus, Hills Road, Cambridge CB2 0QQ, United Kingdom
7. **Contact for Scientific Queries**
   Professor Grant D. Stuart, Department of Surgery, University of Cambridge School of Clinical Medicine, Cambridge, UK, CB2 0QQ, [gds35@cam.ac.uk](mailto:gds35@cam.ac.uk)
8. **Public Title**
   WIRE Novel Treatments in Renal Cancer
9. **Scientific Title**
   WIndow-of-opportunity clinical trial platform for evaluation of novel treatment strategies in REnal cell cancer (WIRE)
10. **Countries of Recruitment**
    United Kingdom
11. **Health Condition(s) or Problem(s) Studied**
    clear cell renal cell carcinoma
12. **Intervention(s)**
    Please refer to the main manuscript for the description of the trial interventions.
13. **Key Inclusion and Exclusion Criteria**
    Please refer to the main manuscript for the description of the inclusion and exclusion criteria.
14. **Study Type**
    WIRE is a Phase II, multi-arm, multi-centre, non-randomised, proof-of-mechanism (single and combination IMPs), platform trial using a Bayesian adaptive design
15. **Date of First Enrollment**
    August 2020
16. **Sample Size**
    Up to a maximum of 76 evaluable participants will be recruited into the initial five arms and across all stages.
17. **Recruitment Status**
    Recruiting: participants are currently being recruited and enrolled
18. **Primary Outcome(s)**
    Please refer to the main manuscript for the description of the primary outcomes.
19. **Key Secondary Outcomes**
    Please refer to the main manuscript for the description of the main secondary and exploratory outcomes.
20. **Ethics Review**
    Status: Approved
    - Date of approval: 07^th^ February 2020
    - Name and contact details of Ethics committee: London Harrow Research Ethics Committee, Level 3, Block B, Whitefrairs, Lewins Mead, Bristol, BS1 2NT, United Kingdom
21. **Completion date**
    study is ongoing
22. **Summary Results**
    not yet available
23. **IPD sharing statement**
    Individual clinical trial participant-level data (IPD) will not be made available.

# Inclusion and Exclusion Criteria

## Inclusion Criteria

To be included in the trial the participant must meet all of the following criteria:

- Capable of giving signed informed consent which includes compliance with the requirements and restrictions listed in the informed consent form (ICF) and in this protocol.
- Aged ≥18 years and over.
- Predicted life expectancy ≥ 16 weeks.
- Eastern Cooperative Oncology Group (ECOG) performance status (PS) 0 or 1.
- Have biopsy proven clear cell RCC, within 6 weeks prior to consent.
- Allow access to archival FFPE tumour tissue from biopsy and nephrectomy.
- Have a surgically resectable tumour as determined by the treating Urologist
- T1b or above, any N status, M0, OR have any T or N status, M1 (but if M1, the participant must be deemed suitable for cytoreductive nephrectomy at time of enrolment).
- No prior exposure to PARP inhibitors (including but not limited to olaparib), tyrosine kinase inhibitors (including but not limited to cediranib, sunitinib, pazopanib, axitinib, bevacizumab and cabozantinib), immunotherapy or immune checkpoint inhibitors (including but not limited to other anti-CTLA-4, anti-PD-1, or anti-PD-L1 antibodies, including durvalumab), nor prior treatment with an mammalian target of rapamycin (mTOR) inhibitor (including, but not limited to everolimus, temsirolimus, or sirolimus). Prior cytokine therapy (eg, IL-2, IFN-α) or treatment with cytotoxics is allowed.
- At least one measurable lesion according to RECIST Version 1.1 at screening that can be accurately assessed at screening by - MRI and is suitable for repeated assessment. A previously irradiated lesion cannot be considered a target lesion. Radiographic disease assessment can be performed up to 28 days prior to the first dose of trial treatment. It is acceptable for the measurable lesion to be planned for removal at surgery. CT reported RECIST assessments are acceptable at screening for participants with chest metastases.
- Have adequate organ and marrow function, as defined below (measured within 28 days of first dose of trial medication):
  - Haemoglobin ≥ 90 g/L
  - Platelet count ≥ 100 x 109/L
  - Neutrophil count ≥ 1.5 x 109/L
  - Creatinine clearance ≥30mL/min (calculated by Cockcroft and Gault equation: where estimated creatinine clearance = (140-age[years]) x weight (kg) (xF)a serum creatinine (mg/dL) x 72a where F=0.85 for females and 1 for males)) Participants with 2+ proteinuria on dipstick must also have UPC <0.5 on 2 consecutive samples.
- Adequate hepatic function:
  - Alanine Aminotransferase (ALT) (SGPT) ≤2.5x the institutional upper limit of normal (ULN) unless liver metastases are present, in which case it must be ≤5x the institutional ULN, AND
  - AST ≤2.5x the institutional ULN unless liver metastases are present, in which case it must be ≤5x the institutional ULN, AND
  - Total bilirubin ≤1.5x the institutional ULN unless in the presence of known or suspected Gilbert’s syndrome- the inclusion of potential participants with known/suspected Gilbert’s syndrome **must** be discussed with the Trial Oncologist prior to their inclusion in the trial.
- Evidence of post-menopausal status or negative serum pregnancy test for female pre-menopausal participants. Women will be considered post-menopausal if they have been amenorrhoeic for 12 months without an alternative medical cause. The following age-specific requirements apply:
  - Women <50 years of age would be considered post-menopausal if they have been amenorrheic for 12 months or more following cessation of exogenous hormonal treatments and if they have luteinizing hormone and follicle-stimulating hormone levels in the post-menopausal range for the institution or underwent surgical sterilization (bilateral oophorectomy or hysterectomy)
  - Women ≥50 years of age would be considered post-menopausal if they have been amenorrhoeic for 12 months or more following cessation of all exogenous hormonal treatments, had radiation-induced menopause with last menses >1 year ago, had chemotherapy-induced menopause with last menses >1 year ago, or underwent surgical sterilization (bilateral oophorectomy, bilateral salpingectomy or hysterectomy).
  - For women of childbearing potential a negative serum pregnancy test must be performed within 28 days of trial treatment and confirmed prior to treatment on day 1.
- Male participants must be willing to use a condom during treatment and for 3 months after the last dose of trial treatment when having sexual intercourse with a pregnant woman or with a woman of childbearing potential. Female partners of male participants should also be willing to use a highly effective form of contraception (see Section 11.12 for acceptable methods) if they are of childbearing potential
- Participant is willing and able to comply with the protocol for the duration of the trial.
- Adequately controlled thyroid function, with no symptoms of thyroid dysfunction

***Specific inclusion criteria for olaparib containing arms:***

- Haemoglobin must be ≥ 100 g/L
- If abnormalities in the Full Blood Count (and it is clinically indicated): Peripheral blood smear with no features of myelodysplastic syndrome or acute myeloid leukaemia
- Serum creatinine must be ≤1.5x the institutional ULN concurrent with creatinine clearance ≥51mL/min (calculated by Cockcroft and Gault equation as above) or based on a 24 hour urine creatinine clearance test.

***Specific inclusion criteria for olaparib plus cediranib arm only:***

- Urine protein:creatinine ratio (UPC) ≤1 OR ≤2+ proteinuria on two consecutive dipsticks taken no less than 1 week apart. Patients with 2+ proteinuria on dipstick must also have UPC <0.5 on 2 consecutive samples.

## Exclusion Criteria

- The presence of any of the following **core** exclusion criteria will preclude participant inclusion:
- cT1a N0 M0-staged Renal Cell Cancer
- Participants with brain metastases. A scan to confirm the absence of brain metastases is not required.
- Participants with spinal cord compression unless considered to have received definitive treatment for this and evidence of clinically stable disease for 28 days prior to start of first dose of treatment.
- History of leptomeningeal carcinomatosis.
- Body weight ≤30kg
- Contraindication to cediranib, olaparib, durvalumab or chimeric or humanized antibodies or fusion proteins.
- Specifically participants with hereditary galactose intolerance, Lapp lactase deficiency or glucose-galactose malabsorption should not enter the trial.
- History of hypersensitivity to active or inactive excipients of cediranib, olaparib or durvalumab.
- Other invasive malignancy within the last 2 years. Participants with previous history of malignancies with a negligible risk of metastasis or death and treated with expected curative intent are eligible at discretion of clinical team, for example:
  - Carcinoma in situ of the cervix.
  - Basal or squamous cell skin cancer.
    - Localized low to intermediate risk prostate cancer treated with curative intent and absence of prostate-specific antigen (PSA) relapse; or prostate cancer (Stage T1/T2a, Gleason ≤ 6 and PSA < 10 ng/mL) undergoing active surveillance and treatment naïve.
- Major surgery within 4 weeks prior to first dose of trial drug (excluding placement of vascular access). If participants have undergone major surgery more than 4 weeks prior to the scheduled first dose of trial drug, they must have fully recovered from the procedure.
- Minor surgery (not including the diagnostic biopsy) within 2 weeks prior to first dose of trial treatment
- Any concurrent chemotherapy, IP, biologic, or hormonal therapy for cancer treatment. Concurrent use of hormonal therapy for non-cancer-related conditions (e.g., hormone replacement therapy) is acceptable.
- Concurrent enrolment in another clinical trial unless it is an observational (non-interventional NOT involving CTIMPs) or translational clinical trial, or during the follow-up period of an interventional clinical trial.
- Receipt of the last dose of anticancer therapy or radiotherapy, chemotherapy, immunotherapy, endocrine therapy, targeted therapy, biologic therapy, tumour embolisation, monoclonal antibodies) ≤28 days prior to the first dose of trial drug*.*
- Gastrointestinal abnormalities including: o refractory nausea and vomiting,
  - inability to take oral medication;
  - requirement for intravenous alimentation;
  - prior surgical procedures affecting absorption including total gastric resection;
  - treatment for active peptic ulcer disease in the past 6 months prior to the first dose of trial treatment;
  - active gastrointestinal bleeding, unrelated to cancer, as evidenced by haematemesis, haematochezia or melena in the past 120 days priort to the first dose of trial treatment without evidence of resolution documented by endoscopy or colonoscopy;
  - malabsorption syndromes.
- Any of the following within 12 months prior to consent:
  - myocardial infarction,
  - uncontrolled angina,
  - coronary/peripheral artery bypass graft,
  - symptomatic congestive heart failure,
  - cerebrovascular accident or transient ischemic attack,
  - peripheral arterial embolus.
- Current or prior use of immunosuppressive agents within 28 days prior to the first day of trial treatment , including anti-TNF and anti-IL17 agents, with the exceptions of intranasal or inhaled corticosteroids, or systemic corticosteroids at physiological doses which are not to exceed 10mg/day prednisolone (or an equivalent corticosteroid). The following exceptions are allowed:
  - Intranasal, inhaled, topical or local steroid injections (e.g. intra articular injection).
  - Systemic corticosteroids at physiological doses not to exceed 10mg/day prednisolone (or equivalent).
  - Steroids for premedication of hypersensitivity reactions (e.g. as CT premedication).
- Immunocompromised participants (e.g., participants who are known to be serologically positive for human immunodeficiency virus (HIV), or have a history of active primary immunodeficiency).
- Active infection including tuberculosis (clinical history, physical examination and radiographic findings, and Tuberculosis (TB) testing in line with local practice), hepatitis B (known positive HBV surface antigen (HBsAg) result), hepatitis C, or human immunodeficiency virus (positive HIV1/2 antibodies).
  - Participants with a past or resolved HBV infection (defined as: presence of hepatitis B core antibody -anti-HBc- and absence of hepatitis B surface antigen –HbsAg-) are eligible.
- As judged by the Investigator, any participant considered a high medical risk due to a serious uncontrolled medical or psychiatric disorder, non-malignant systemic disease or on-going or active infection.
- Persistent toxicities (≥Common Terminology Criteria for Adverse Event (CTCAE) grade 2) caused by previous cancer therapy, excluding alopecia and vitiligo.
  - Participants with Grade ≥2 neuropathy will be evaluated on a case-by-case basis after consultation with the Chief Investigator.
  - ***For durvalumab-containing arms only:*** Participant with irreversible toxicity not reasonably expected to be exacerbated by treatment with durvalumab may be included only after consultation with the investigator.
- Women who are pregnant, or are lactating or breastfeeding.
  - Women of childbearing potential and male participants who are unwilling to use adequate contraception from consent and for 3 months after the last dose of trial drug.
- Participants with contraindication to MRI including; contraindicated metallic implants, contraindicated coronary stents and pacemakers. Inability to lie flat or still in an MRI scanner for whatever reason (e.g., claustrophobia).
- Judgement by the Investigator that the participant should not participate in the trial.
- Involvement in the planning and/or conduct of the trial
- Previous allogeneic bone marrow transplant or double umbilical cord blood transplantation.

***Specific exclusion criteria for cediranib and/or olaparib containing arms:***

- Current use or anticipated need for treatment with drugs that are known potent CYP3A4 inhibitors, or inducers or substrates for CYP1A2 (see Section 10.8, Concomitant therapy and Appendices 4 and 5 for full details).
- Concomitant medications known to prolong the QT interval (see Appendices 4, 5 & 6 , Concomitant therapy) or with factors that increase the risk of QTc prolongation or risk of arrhythmic events (such as heart failure, hypokalaemia, congenital long QT syndrome, family history of long QT syndrome or unexplained sudden death under 40 years-of-age), history of Torsades de pointes.
- Resting ECG indicating uncontrolled, potentially reversible cardiac conditions as judged by the investigator (eg., unstable ischaemia, uncontrolled symptomatic arrhythmia, congestive heart failure, QTcF prolongation >500ms, electrolyte disturbances, resting QTc of ≥470ms (Fridericia; as per local reading), etc.) on two or more time points within a 24 hour period or family history of long QT syndrome.
- Requirement of anticoagulant therapy with oral vitamin K antagonists.
  - Therapeutic use of low molecular weight heparin is allowed.
- Poorly controlled hypertension (persistently elevated > 150/100mmHg (or > 140/90 ***for olaparib plus cediranib arm only***), either systolic or diastolic or both, despite anti-hypertensive medication)
- Clinically significant signs and/or symptoms of bowel obstruction within 3 months prior to starting treatment
- History of intra-abdominal abscess within 3 months prior to starting the first dose of trial treatment
- History of GI perforation. Participants with a history of abdominal fistula will be considered eligible if the fistula was surgically repaired, there has been no evidence of fistula for at least 6 months prior to starting the first dose of trial treatment, and participant is deemed to be at low risk of recurrent fistula

***Specific exclusion criteria for cediranib monotherapy or combination therapy arms only:***

- Left ventricular ejection fraction (LVEF) < lower limit of normal (LLN) per institutional guidelines, or <55%, if threshold for normal not otherwise specified by institutional guidelines, for participants with the below risk factors (an Echocardiogram should be performed at baseline and if clinically indicated):
  - Prior treatment with anthracyclines
  - Prior treatment with trastuzumab
  - Prior central thoracic RT, including exposure of heart to therapeutic doses of ionising RTM
  - Prior history of other significant impaired cardiac function

***Specific exclusion criteria for durvalumab containing arms only:***

- Receipt of live, attenuated vaccine within the last 30 days. Note: enrolled participants should not receive live, attenuated vaccine while receiving durvalumab nor within 30 days of last dose of durvalumab.
- Active or prior documented autoimmune or inflammatory disorders (except vitiligo), for example:
  - Intestinal: Inflammatory Bowel Disease (Colitis (including ulcerative colitis), Crohn’s Disease), Diverticulitis (with the exception of Diverticulosis), Coeliac Disease (except participants with coeliac disease controlled by diet alone), irritable Bowel Disease
  - Vascular: any type of vasculitic disorder, e.g. Wegener syndrome, granulomatosis with polyangiitis.
  - Endocrine: any endocrine alteration related to an autoimmune process e.g. Hashimoto syndrome, Grave’s disease. NOTE: participants with hypothyroidism (eg, following Hashimoto syndrome) stable on hormone replacement treatment may be included.
  - Respiratory: Active Pneumonitis (of any origin: inflammatory or infectious), Sarcoidosis syndrome.
  - Dermatological: Psoriasis, Lupus/SLE (unless the skin condition has never required systemic therapy).
  - Other: Rheumatoid Arthritis, Hypophysitis, Uveitis.
  - History of organ transplant that requires use of immunosuppressive medications or any medical condition in which immunosuppressive agents were administered, including but, not limited to: Systemic corticosteroids, methotrexate, azathioprine.
  - Tumour necrosis factor alpha (TNF-α) blockers
  - Participants with autoimmune conditions without active disease in the past 5 years may be included but only after discussion with the Trial Medical Oncologist.
- Participants with persistent toxicities (≥Common Terminology Criteria for Adverse Event (CTCAE v 5.0) grade 2) caused by previous cancer therapy, excluding alopecia and vitiligo, which are not reasonably expected to be exacerbated by treatment with durvalumab may be included only after consultation with the Chief Investigator.

# Relevant concomitant care permitted or prohibited during the trial

Any concurrent chemotherapy, IMP, biologic, or hormonal therapy for cancer treatment is not allowed. Concurrent use of hormonal therapy for noncancer-related conditions (e.g., hormone replacement therapy) is acceptable. Receipt of the last dose of previous anticancer therapy (radiotherapy, chemotherapy, immunotherapy, endocrine therapy, targeted therapy, biologic therapy, tumour embolisation, monoclonal antibodies) must be at least 28 days prior to the first dose of IMP. Major surgery must be completed 4 weeks prior to the first dose of IMP (excluding placement of vascular access) and if participants have undergone major surgery more than 4 weeks prior to the scheduled first dose of IMP, they must have fully recovered. Minor surgery (not including the diagnostic biopsy) is allowed within 2 weeks prior to first dose of IMP.

In arms containing cediranib or olaprarib, drugs not allowed include known potent CYP3A4 inhibitors, or inducers or substrates for CYP1A2, concomitant medications known to prolong the QT interval, and anticoagulant therapy with oral vitamin K antagonists (therapeutic use of low molecular weight heparin is allowed). For durvalumab containing arms receipt of live, attenuated vaccine within the 30 days before or after durvalumab is not allowed, nor use of immunosuppressive agents within 28 days prior to the first dose of trial treatment, including anti-TNF and anti-IL17 agents, with the exceptions of intranasal, inhaled, topical or local steroid injections (e.g. intra articular injection), or systemic corticosteroids at physiological doses which are not to exceed 10mg/day prednisolone (or an equivalent corticosteroid or use of steroids for premedication of hypersensitivity reactions (e.g. as CT premedication).

Concurrent enrolment in another clinical trial is not allowed unless it is an observational (non-interventional not involving investigational products) or is a translational clinical trial, or during the follow-up period of an interventional clinical trial.

# Strategies to improve adherence to interventions

Participants will be asked to keep a diary of their medication intake and to return the empty packaging and remaining doses of IMP to each visit. Regular clinic visits or telephone calls will ensure additional support.

# Participant retention

In addition to the formal assessment points, patients will have direct access to a designated trials nurse in working hours and 24hr access to a trained oncology nurse for discussion of any medical issues. Dose adjustments and treatment interruption will be permitted within the boundaries outlined above. However, if a participant cannot continue in the trial or wishes to stop, data collected until the point of discontinuation will be retained and analysed where possible.

# Provisions for post-trial care

Following the completion of the trial after the follow-up visit three months post-surgery, patients will return to standard-of-care, risk-adapted follow-up as per the procedures at the recruiting medical centre.

# Oversight and Monitoring

## Composition of the coordinating centre and trial steering committee

The coordinating centre comprises of a clinical trial coordinator, data manager, and trial monitor. The clinical trial coordinator provides ongoing project management, local operational support, and management of external sites. The clinical trial data manager ensures statistical information and results from the trial are recorded accurately and oversees the eCRF. The trials monitor provides external oversight and ensures adherence to the trial protocol, local standard operating procedures, and good clinical practice (GCP).

## Composition of the data monitoring committee, its role and reporting structure

The independent data and safety monitoring committee (IDSMC) will meet to review the interim statistical report of the trial management group following the conclusion of each stage within a respective treatment arm. The IDSMC will make recommendations on the continuation of the overall trial and primary endpoint data for the individual arms. Subsequent IDSMC meetings will be undertaken 6-monthly during which the committee will review a full report of the trial progress. The IDSMC is formed of one urologist, oncologist, radiologist, pathologist, and statistician. The independent trial steering committee (TSC) will review recommendations made by the IDSMC and consider participant evaluability reports from the trial. Based on these recommendations and reports, the TSC will decide whether the subsequent treatment arms are to open (due to proof of mechanism or stopping for futility), or to re-open existing or previous treatment arms (due to insufficient data or participants not meeting evaluable criteria).

## Frequency and plans for auditing trial conduct

The sponsor pre-initiation risk assessment stipulates monitoring visits every 2 months, falling to 3 months based on the number and severity of the findings from preceding monitoring visits. Participating sites undergo monitoring on a similar schedule and frequency, with added monitoring visits corresponding with recruitment milestones. Additionally, serious adverse events and protocol deviations may trigger monitoring visits. The MHRA may audit the trial.

## Plans for communicating important protocol amendments to relevant parties

Following guidance from the Health Research Authority, protocol amendments require proportionate or full review by the ethics committee and approval by the HRA. Trial participants will be informed of changes and re-consented only if they are affected by the changes.
