# Supplementary material for: The WIRE study a phase II, multi-arm, multi-centre, non-randomised window-of-opportunity clinical trial platform using a Bayesian adaptive design for proof-of-mechanism of novel treatment strategies in operable renal cell cancer – a study protocol
Source: BMC Cancer. 2021 Nov 18;21:1238. doi: 10.1186/s12885-021-08965-4 (PMC8600815; doi:10.1186/s12885-021-08965-4)
Supplement: Supplementary file 3 — Additional file 3. [file 12885_2021_8965_MOESM3_ESM.pdf]

TO BE PRINTED ON HEADED PAPER

UNIVERSITY OF CAMBRIDGE

Cambridge University Hospitals NHS
NHS Foundation Trust

WIRE
NOVEL TREATMENTS IN RENAL CELL CANCER

<u>Group 1 – Cediranib Only</u>

<u>PARTICIPANT INFORMATION SHEET & INFORMED CONSENT FORM</u>

WIRE: <u>WI</u>ndow-of-opportunity clinical trials platform for evaluation of novel treatment strategies in **<u>RE</u>**nal cell cancer.

You are being invited to take part in a research trial. Before deciding whether to take part, you need to understand why this research is being done and what it involves.

Please take time to read the following information carefully and talk to others about the trial if you wish. Please ask your trial team if anything is not clear or if you would like more information. Please take time to decide whether or not you wish to take part. You will be receiving this Participant Information Sheet before any diagnosis has been made i.e. it is not yet confirmed that you have renal cell cancer.

Section 1 tells you the purpose of this trial and how the trial would involve you, including a description of what the various tests are, and some of the risks and restrictions of taking part

Section 2 gives you more detailed information about the trial process, including information about how the trial is run, and how we may use your data.

Section 1: Purpose of the trial and what will happen

1. What is the purpose of the trial?

Kidney cancer is the 7th most common cancer in the UK. Currently many patients with this cancer are offered a kidney removal operation (known as nephrectomy), which cures many patients with no further need for treatment. However, some may receive drug treatment after surgery.

WIRE is a trial that will administer drugs to patients in the time between the decision to operate and the nephrectomy operation. Patients are not usually given anti-cancer drugs during this period, and indeed may never need them, but it could give researchers important information to guide the best combinations of anti-cancer drugs to prioritise for later phase clinical trials. The trial will investigate the effect and safety of 3 different cancer drugs, individually or in combination, in patients with a specific type kidney cancer called clear cell renal cell cancer. The cancer may be localised (within the kidney) or metastatic (it has spread outside of the kidney).

2. What are the drugs being tested?

There are 3 drugs being used in this trial, Cediranib, Olaparib and Durvalumab. All three of these drugs target different parts of cancerous cells which might decrease the size of the tumour over a period of time. However, the duration of treatment in this trial is very short in comparison to how long these drugs would normally be given for. Therefore, it is unlikely that the trial treatment will noticeably reduce the tumour size if it is confirmed that you do have kidney cancer. . In this trial, we will primarily be looking at cancer's response to these drugs by taking a closer look at the tumour cells in the laboratory and by using new imaging technology.

Although the trial uses 3 drugs, we will only be giving you one or two of these drugs. The drugs you are given will depend on the group you are allocated. The trial team already know which group you will be allocated. Currently, we are recruiting participants for the **Cediranib** group. Each group has its own specific entry criteria which may involve different tests and assessments; this is discussed further under *Section 4 Do I have to take part?*

Cediranib is a targeted cancer therapy drug called a 'growth factor blocker'. It binds to a specific protein on the surface of the cancer cells which controls blood vessels' development (vascular endothelial growth factor (VEGF)). By blocking the usual action of this protein cediranib may reduce blood supply to the tumour and may slow down the growth and spread of the cancer. Cediranib is being tested in clinical trials, but is not currently licenced in the EU. This drug is an oral tablet (a tablet taken by mouth) which will be taken for at least 14 days in total, and until 36 hours before your surgery.

3. Why have I been invited?

You have been invited to participate in this trial because you may have renal cell cancer (kidney cancer) that may not be cured with surgery alone. The diagnosis of renal cell cancer will be confirmed or disproved with a pre-surgery biopsy. In normal care, usually your treating doctor will recommend proceeding with a nephrectomy or partial nephrectomy (removal or partial removal of the kidney) without the need of a pre-surgery biopsy. However, as part of the WIRE trial, participants will be required to undergo a biopsy during the screening stage to determine whether they have the type of cancer we are investigating.
We plan to include up to 76 patients with kidney cancer from approximately 2 hospitals across the UK.

4. Do I have to take part?

Participating in this trial is completely voluntary. If you decide to participate you will be asked to sign an Informed Consent Form. You are however still free to change your mind and leave the trial at any time and without giving a reason. If you chose not to participate or to leave the trial, your current and future medical treatment will not be affected in any way. Your normal standard care will not be affected in any way.

You should also be aware that entry into this trial requires you to meet some strict entry criteria that your doctor will assess you for over the next few weeks, during a period called 'screening'. At the moment we will not know whether you are eligible for the trial as there are further tests and assessments to do, but some people who consent to take part in this trial may not meet the entry criteria to receive the trial drugs.

Before proceeding, it is important to understand that you may not benefit from the extra drugs and procedures involved in this trial. Surgery alone may be sufficient to cure the cancer alone, although there is no way to predict this for certain. The possible side effects could potentially delay your surgery, and participation in the trial could make you feel

fatigued and nauseous. However, your doctors will be monitoring how you are feeling very closely, making every attempt to minimise any side effects.

It is also important you are aware that based on previous research the average chance of being cured by surgery alone needing no further treatment is 42%, in whom no further treatments would be needed. It is also important that you are aware that you will not have access to the trial drugs after your operation, and you might not be permitted to take part in other clinical trials if you decide to participate in the WIRE trial.

5. Expenses and payment

You will not receive any payment for participating in this trial however we can reimburse reasonable travel and parking costs incurred by your participation in this trial. This is up to a maximum of £180 for the entire duration of your participation in this trial. We will ask you to retain your receipts wherever possible.

6. What are the possible advantages and disadvantages of taking part?

It is unlikely that you will benefit clinically from taking part in this trial, although it is possible that the tumour may shrink very slightly. It is also hoped that taking the medications used in the trial could provide you with a better outcome, but this cannot be directly measured in this trial. You will gain access to drugs that are only available within a clinical trial. You are likely to have more contact and for longer with your care team than if you were not involved in a trial, and some patients gain reassurance from this. It is hoped that information collected as part of your participation in this trial will benefit patients with kidney cancer in the future and you may contribute to their future treatment and care. Your care team will be able to share information with you regarding how the tumour cells responded and/or any tumour size reduction, if you wish to know this.

In addition to the above benefits there are also aspects of your participation you may find difficult. For instance, if you decide to take part:

- You will need to undergo a biopsy at the screening stage which is likely to require a full day at the hospital. It is also likely that this biopsy would not have been required if you were not participating in this trial. This is an extra procedure for about 70% of patients. You can find more information about this under section 9 – Tumour biopsy on page 12.
- There will be an increased number of hospital visits due to participating in the trial. You will be required to come to the hospital an extra 4-5 times during the trial. These visits are to conduct initial assessments and scans (1 extra visit); clinic visits whilst receiving the trial drug(s) (2-3 extra visits); a visit for the scan before surgery (1 extra visit). The average length of time per visit is about 2 or 3 hours, but actual time may vary. The schedule and timing of these visits is available on page 9.
- You will undergo at least an additional 2 MRI scans if you decide to take part in this trial, with potentially another 2 MRI scans using a new type of scan called a 'hyperpolarised MRI' if available at your hospital (see section 9, pages 11 and 12 for more information). An additional 1-2 CT scans may be required as recommended by the doctor based on information from your diagnosis and previous scans. You would not have normally received these scans if you were not participating on the trial.
- You could experience drug side effects such as nausea, fatigue, diarrhoea, dizziness, decreased appetite and blood test abnormalities. A full list of known

side effects is listed on pages 15 and 16. It is also possible that some of these side effects might delay your surgery.

- There will be 6 additional tissue biopsies (samples) taken during your surgery, this will likely only add a few minutes to your standard surgical procedure. Whilst these are unlikely to impact you or prolong your stay in hospital, it is important that you are aware these are taken. More information about these tissue samples is mentioned on page 13 and 14.
  The drugs you will be taking as part of this trial will not be given to you after the surgery, and may prevent you from taking part in other clinical trials after your surgery.

7. What are the alternatives for treatment?

The standard care for you is a nephrectomy (removal of the kidney) or partial nephrectomy (removal of the tumour only) without any drug treatment before surgery.

8. What happens when the trial stops?

Following completion of the trial you will not be able to receive any further trial drug treatment and we will refer you back to your primary consultant. You will still be able to contact your trial Nurse or Practitioner, however, with any questions about the trial.

9. What will happen to me if I take part?

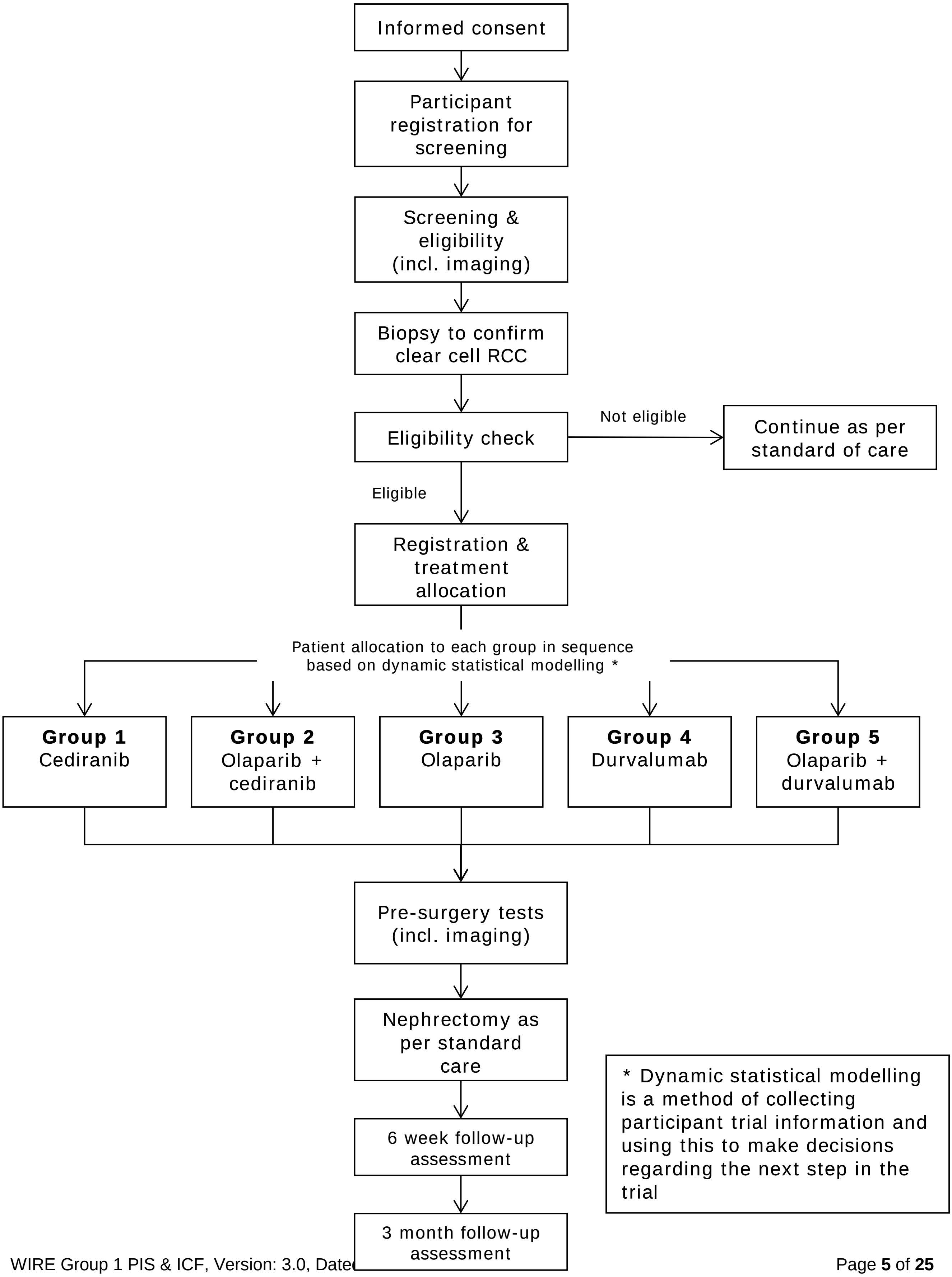

Hospital Appointments

Informed Consent
If you agree to participate in the trial, you will be invited to sign the Informed Consent Form at the end of this document. You will be given a copy of this to take away and refer to later.

Screening and baseline
Standard of care visit plus extra trial assessments 5-6 hours in duration. Screening may require an additional visit to complete all assessments, dependent on hospital capacity.
To check that you meet all of the requirements for entering this trial you will need to have some tests. This is called the "screening period". You will be given appointments to see the trial team for these tests, which will be carried out within a 28 day period. This may include more than one visit to the hospital. All of the tests listed during this period and the following visits are further explained later in this information sheet (see page 5). All of the tests listed under Screening will collect data for the research trial.

- A check of your weight, blood pressure and temperature
- A full physical examination
- Clinical review
- Performance status check
- Blood and urine sampling
- 3 Electrocardiogram assessments (ECGs), 5 minutes apart.
- A minimum of one type of scan, including a Dynamic Contrast Enhanced Magnetic Resonance Imaging (DCE-MRI) scan at least (a type of scan that uses strong magnetic fields and radio waves to produce detailed images of the body and organs). You may undergo a hyperpolarised MRI scan (a new MRI scanning technique which produces extremely detailed images of the body and organs), depending on the group you are allocated to, and the treatment centre you are attending. You may also undergo a CT scan, depending on how long ago your last CT scan took place (the risks of increased radiation exposure are explained further on in this document, in the 'Tests and Assessments' section).
- Collection of your Medical History
- Research blood and urine sampling- we will take these samples before you have the tumour biopsy, on the same day
- Tumour Biopsy
- Pregnancy test (female participants only)

Your weight, blood pressure, temperature, physical examination, performance status check, blood & urine samples, CT and ECGs may be assessed as part of your standard care pathway (these would usually take place without trial participation anyway as part of the usual pre-surgery checks). A tumour biopsy would not normally take place in about 70% of patients, but all participants will have a biopsy in order to be eligible for the trial.

Additional ECGs, a possible repeat CT scan (if you haven't had one within the last 28 days) and all of the other assessments and scans listed are extra for the trial.

Registration
Some of the tests performed during Screening require the results to fall within certain trial ranges. If any of your results do not fall within these ranges, you will not be able to participate in this trial and will be referred to follow the usual treatment pathway for patients with your condition. However, if the screening tests show that you are suitable to participate in this trial, your clinical team will register you into the trial.

There are 5 different treatment groups in this trial, but each participant will only be allocated to one of these groups, and given the appropriate information sheet for the drug that they might receive. If you decide to participate and meet the entry criteria, you will be allocated to Group 1 and be given cediranib only.

Clinic Visits

Extra visits for the trial, up to 3 hours per visit in duration

Once your participation in this trial has been confirmed we will ask you to attend regular clinic appointments to see the trial team whilst you are receiving the trial drug/s and we will perform a number of trial related tests to ensure you are tolerating the trial drugs well. Initially these will be performed on the 1st and approximately 15th day of your treatment but will be flexible around weekends and bank holidays. You will need to have fasted for 6 hours before the Research urine tests, and your research/care team will advise you regarding the timescales on this. Both of these visits are extra visits for the trial and all assessments for these visits below are needed just for the trial and are outside of standard care:

- A weight, blood pressure and temperature check
- A physical examination (if your doctor believes it is necessary)
- Performance status check
- Clinical review
- At least 1 electrocardiogram assessment (ECG). Based on your results, your trial team may ask you to perform 2 additional ECGs, all 3 taken 5 minutes apart.
- Blood and urine assessments. Pre-treatment (Day 1)
- Pregnancy test (female participants only)
- Dispensing of your tablets to take home, and a diary to record when you take them (at Day 1 only).

In addition to the assessments above, the following tests will be performed on Day 15 ONLY

- Research blood and urine sampling
- Assessment of any side effects from the medication
- Check that you have enough and are correctly taking, your medication

Your clinical trials team may decide to give you an additional assessment on approximately the 22nd day of treatment, if you are still taking the trial drug. This may be by telephone or in clinic; this day 22 assessment will be an extra assessment for the trial, and the below tests will be extra assessments for the trial, outside of standard of care:

- Performance status check
- Assessment of any side effects from the medication
- Check that you have enough and are correctly taking, your medication
- Clinical review

If you experience unmanageable side effects, or if your trial doctor thinks you should not continue, the trial drugs will be stopped and you will return to the normal standard of care provided by the hospital for the treatment of the cancer.

Pre-surgery

Extra visit, up to 6 hours in duration

Within 3 days of your surgery date, you will undergo at least one scan to assess whether the medication you have been taking is affecting the tumour. These are all extra assessments for the trial, outside of standard care, and the visit could take most of a day or more than one day:

- DCE-MRI
- Hyperpolarised MRI (depending on the group you are allocated to, and the treatment centre you are attending)
- CT scan (if the cancer has spread outside the kidney- your Trial Nurse or Practitioner will advise you)
- Research blood and urine sampling (these are outside of standard care and are required for the trial only)
- Routine blood sampling

<u>Surgery</u>
Standard of care visit. Surgical prep plus 3 hours surgery time.
During your nephrectomy, we will obtain tumour samples, as well as samples of the normal kidney tissue next to the tumour. The surgery is standard care but the amount of samples taken will be higher than in standard care.

<u>Follow-up – 6 weeks after surgery</u>
Standard of care visit, up to 2 hours in duration
You will be given an appointment to see the trial team approximately 6 weeks after surgery to ensure that you have recovered from any side effects. This 6 week check is usual for patients who have undergone nephrectomy, and is standard care. During this visit you will have:

- Performance status check
- Clinical review
- Routine Blood sampling
- Pregnancy Test

<u>End of trial follow-up – 3 months after surgery</u>
Standard of care visit, up to 2 hours in duration
You will be given another appointment to see the trial team approximately 3 months after surgery to ensure that you have tolerated the trial drugs well. A CT scan will be performed prior to this visit. Following this visit, you will not need to attend any further trial related visits. This 3 month check is usual for patients who have undergone nephrectomy, and is standard care. During this visit you will have:

- Performance status check
- Clinical review
- Routine blood sampling
- Research blood and urine sampling (these are outside of standard care and are required for the trial only)
- Pregnancy test

UNIVERSITY OF CAMBRIDGE

Cambridge University Hospitals NHS
NHS Foundation Trust

What will happen and when?

Tests and Assessments	Screening (up to 28 days prior to Day 1 of treatment)	Treatment phase			Pre-surgery	Surgery	Follow-up	
		**Day** 1 (± 5 days)	**Day 15** (± 5 days)	**Day 22 (Optional)** ± 5 days			6 weeks after surgery ± 7 days	3 months after surgery ± 14 days
Consent	X							
Medical history	X							
Vital signs	X	X	X					
Performance status check	X	X	X	X			X	X
Clinical review	X	X	X	X			X	X
Physical examination	X	If clinically necessary	If clinically necessary					
ECG	X	X	X					
Urine sample	X	X	X				X	X
Blood for routine tests	X	X	X		X		X	X
Pregnancy test	X	X	X					X
Post-menopausal test	X							
Blood for research tests	X		X		X			X
Urine for research tests	X		X		X			X
DCE-MRI	X				X			
Hyperpolarised MRI	If needed				If needed			
CT scan (*your doctor will tell you if needed)	X*				If needed*			X
Tumour biopsy (screening)	X							
Cediranib Drug dispensing		X						
Cediranib Drug compliance check			X	X				
Tissue samples (research)						X		
Full or partial nephrectomy						X		
Total time of visit	5-6 hours over 2-3 visits	2-3 hours over 1 visit	2-3 hours over 1 visit	1-2 hours over 1 visit or 20 min phone call	4-6 hours over 1-2 visits	2-3 hours of surgery	1-2 hours over 1 visit	1-2 hours over 1 visit

Tests & Assessments

You will be monitored carefully by the trial team whilst you are on this trial. Most of the tests in this trial are necessary to give you the trial drugs safely and ensure you are tolerating the trial drugs well. These tests are explained below, including the possible **risks and/or benefits** of each.

Medical history
Your medical history will be reviewed by the trial team during the screening period. This will involve a discussion about your past and present health, lifestyle and any symptoms you may currently have. This is so that the trial team are aware of any pre-existing conditions. The trial team will also record your gender, ethnicity and date of birth. This is to collect demographic data for the trial.

Weight, blood pressure and temperature
The trial team will monitor your blood pressure and temperature in order to properly assess the side effects to your treatment. These indicators will help the doctors to properly diagnose you and to manage your care.

Clinical review
The trial team will regularly discuss and review your general wellbeing, discuss any symptoms and/or side-effects that you may have, and record any medications you may be taking. This is so that the trial team has a full picture regarding your current state of health.

Your trial team may end your participation in this trial if you have severe or unmanageable side-effects to the trial drug/s. If this happens your trial team will follow-up with you regarding your progress until the side effect has stabilised or resolved.

Physical examination
Your trial doctor will perform a physical examination during the screening period and whilst you are receiving the trial drugs. This will ensure that the trial team has a full picture regarding your current state of health.

Drug Compliance Check
It is important that you take the trial drugs as directed by your trial team. We will provide you with a diary to log the medication that you are taking as part of this trial (cediranib). You must also return any tablets or the empty container to the trial team at the end of your treatment and bring your diary with you to each visit.

Electrocardiogram (ECG)
You will be asked to have several ECGs as part of this trial during the screening period and whilst you are receiving the trial drugs. These record an electrical trace of your heart rhythm and are required to confirm that you are fit enough to receive the trial drugs. They will also monitor the health of your heart during the trial. We will ask you to lie down whilst a series of leads are placed on your chest area, wrists and ankles with adhesive pads, these will record a trace of your heart rhythm. This takes a few minutes and is painless. This is to check the health of your heart.

Echocardiogram (ECHO)
This assessment will ONLY be performed at screening if your trial clinician thinks it necessary, and ONLY if you are on one of the cediranib groups of the trial. This is to check the function of your heart. There may be repeat scans during the trial to check on your heart's function.

Performance Status check

Your performance status check will track the impact that your condition or treatment has on your day-to-day activities and on your "usual" level of day-to-day functioning. This is assessed by asking you questions about your level of activity around the house or at work (as applicable).

Pregnancy test/ Non-childbearing status (female participants only)

If you are female and of childbearing potential you will be asked to give a blood sample during the screening period **to confirm that you are not pregnant before you can be enrolled into this trial.** You will also have several pregnancy tests during the trial. If you are of non-childbearing potential you may be asked to provide a sample of blood during the screening period to confirm your non-childbearing status. This is because we do not yet know the effect of the trial drugs on a developing baby (foetus). This is an extra sample but can be taken from the same bottle as the other blood tests. Most female patients would normally have a urine test.

MRI Scans

You will undergo two DCE-MRI scans if you participate in this trial, and may also have two hyperpolarised MRI scans. It is important that you are aware that the MRI scans would not have been required if you were not participating on this trial. You may need an injection of a contrast agent to improve the quality of the scan. The injection is usually given in a vein in your elbow (the same place as a blood test), which may cause discomfort and/or bruising. When you have the scan, you will be asked to lie still on a couch which will slide into a metal tunnel, which some people find this uncomfortable and/or claustrophobic. The scan lasts for about 45-60 minutes. You will also be able to talk to the radiographer whilst you are having your scan, or listen to music if you prefer. If you are anxious in enclosed spaces you may be prescribed a tablet to help you relax before you have the scan.

DCE-MRI scan

We will ask you to have a DCE-MRI scan during the screening period and before your surgery. DCE-MRI is a type of scan that uses strong magnetic fields and radio waves to produce detailed images of the body and organs. We do this to establish how the trial drugs affect blood flow in the tumour and how the cancer is responding to the trial drugs.

It is known that small amounts of these contrast agents may remain in the brain after a scan with these agents, although there is currently no evidence that these small amounts cause any harm and these agents have been used for many decades. These contrast agents are routinely given during a kidney cancer MRI so we can have a clear understanding of the blood supply to the cancer. You may experience some side effects such as nausea, headaches and dizziness which are very common side effects. Other side effects such as vomiting, injection site reaction, laboured breathing, and allergic reactions are less common. You should tell a staff member immediately if you experience any of these symptoms so you can be treated promptly. We will use the lowest dose of the contrast agent required for a clear image and if you have any questions about your scan, please speak to your doctor.

Hyperpolarised MRI

You may also be asked to have hyperpolarised MRI scans during the screening period and before your surgery. These scans will enable researchers to assess the cancer tumours and the effectiveness of trial drugs. Not every participant will receive this scan, depending on the numbers already in the trial and local availability.

The contrast agent to be injected is a breakdown product of sugar called pyruvate. Although hyperpolarized carbon MRI is a new technology, tests to date have demonstrated no significant safety issues. Pyruvate is a naturally-occurring molecule in the body. Rarely, people who have had an injection of pyruvate in previous studies developed: unusual taste within the mouth, headaches, flushing, diarrhoea or dizziness after the injection. These side effects were mild and short lasting. Although it is unlikely that an allergic reaction or other side effect will occur, there are facilities in place within the MRI unit, and within the hospital, to manage these appropriately. If you have any questions about your scan, please speak to your doctor.

Computerised Tomography (CT) scan

You may have a CT scan during the screening period (depending on how recent your last one was) and, if required, approximately 3 days before your surgery (you will be told in advance if this includes you). You will also have one just before your 3 month follow up visit. CT scans are used to take pictures of, measure and assess cancer tumours.

When you have the scan, we will ask you to lie still on a couch which will slide through the scanning machine. The scan lasts about 30 minutes, sometimes longer. You may need to drink or have an injection of a contrast agent into a vein which improves the quality of the scan images. You may however be observed for some time after the CT scan to check for a reaction to the contrast agent, which is very rare. Some common side effects that you may experience is feeling hot. Some uncommon side effects include feeling sick, increased sweating, feeling cold, and dizziness. Allergic reaction symptoms can include mild itching or hives (small bumps on the skin) to shortness of breath and swelling of the throat or other parts of the body. You should tell a staff member immediately if you experience any of these symptoms so you can be treated promptly

Some CT scans are part of standard care and you would have had these anyway, whether you take part in the trial or not. Participation in this trial may mean that you receive 1-3 additional CT scans compared with normal care, depending on your result. These procedures use ionising radiation to form images of your body. Ionising radiation can cause cell damage that may, after many years or decades, turn cancerous. The chances of this happening to you as a consequence of taking part in this trial are approximately 0.3 %. If you are concerned about the level of radiation you are having or have had please talk to your trial team.

Tumour biopsy

You will have a tumour biopsy (removal of a sample of tumour tissue with a special needle) during the screening period. The sample will be around the size of two long grains of rice used to diagnose that you have the type of kidney cancer (clear cell) which may confirm that you are eligible for WIRE. The biopsy will be performed in hospital. A type of imaging (e.g. ultrasound) may be used to help guide the needle and reduce the risk of complications. You will receive a sedative to make you comfortable throughout the procedure. The total duration of the tumour biopsy is 30 minutes, plus an observation period of 6 hours. This period is to manage any side effects appropriately. There may be pain, discomfort and/or bleeding as a result of a biopsy.

This is a research procedure for most participants, performed after your DCE-MRI scan.

Routine Blood and Urine sampling

We will ask you to give blood and urine samples for safety purposes throughout the trial to ensure that you are well enough to receive the trial drugs, and to monitor for any trial drugs

side effects that you may be suffering from. Please see '*Research biosamples*' for a description of the amount of blood and urine that we are taking.

Kidney Surgery (Nephrectomy)
The nephrectomy (surgical removal of a kidney) or partial nephrectomy (removal of the tumour from the kidney) will be performed by your hospital. The kidney surgery is standard of care and should not be affected as a result of taking part in this trial. It is important that you inform the trial team of any side effects as early as possible to ensure they do not affect your surgery should you not suffer serious conditions causing a delay.

Research biosamples
We will ask you to donate extra blood, urine and tissue samples for research related to this trial. These samples will be used to gain more detailed information on how your body and the cancer cells are affected by the trial drugs, and to give further details regarding the biology of the cancer.

The specific details of the research samples taken during this trial are given below.

Research Blood samples
The total amount of extra blood we will take from you for research related to this trial depends on the treatment schedule you are allocated to. We will take a maximum of 31mls of blood at each appointment (roughly 6 teaspoons). Of this 31mls, approximately 13mls will be used for your routine lab tests, and 18mls will be used for research purposes. These blood samples will be taken alongside routine bloods wherever possible. You may experience some discomfort when they are taken. These are timed as follows:

- During the screening period (Routine + Research - 31mls).
- On Day 1 (Routine ONLY - 13mls).
- On Day 15 (Routine + Research - 31mls).
- On the visit that takes place 3 days before surgery (Routine + Research - 31mls).
- At the End of Trial Follow-up visit, 3 months after surgery (Routine + Research - 31mls).

Research Urine samples
We will ask you to give extra urine for research related to this trial, and we may also use these samples to investigate the broader biology of the cancer. You will need to have fasted for these samples- your Research team will advise you regarding the requirements.

Research Tumour Tissue samples
During your nephrectomy, extra small samples of tumour and normal kidney tissue will be taken for research just prior to disconnecting the kidney from its blood supply. This procedure is extra and therefore would not normally be done. It will likely only add around 1 minute to your surgery. Each extra sample we take will be around the size of two long grains of rice put together lengthways, end-to-end. We will aim to take 6 samples. There is an additional risk of bleeding from this biopsy, which is predicted by the Investigators to be very small as your kidney will be removed moments later. This biopsy is important for the research: By looking at this sample (taken from this kidney whilst it still has blood flow), researchers can get the fullest picture of its activity following the drug treatment.

Following the nephrectomy, in the pathology department, the pathologist will take extra tissue from the kidney tumour and normal kidney tissue for research.

10. Are there any additional requirements?

If you agree to take part in this trial, there are a number of things that you must and must not do.

Hospital appointments, tests and assessments

You will need to attend all of the trial hospital appointments arranged by your trial team and agree to the tests needed for this trial.

Other medications

There are certain medications which you cannot take whilst you are receiving the trial drug(s) as the interaction between the medication and the trial drug(s) is not yet known. You must inform your trial doctor of any medications, over the counter medications, supplements, herbal remedies or alternative therapies you are taking or using. Your trial doctor will inform you if any changes are needed. You will also need to check with your trial team before taking any newly prescribed, over the counter medications, supplements, herbal remedies or therapies.

Other clinical trials

Whilst you are receiving the trial drugs you will not be able to participate in any other interventional research studies. It is possible that your participation in this trial may stop you being able to take part in some clinical trials in the future, depending on the nature of that trial.

Contraception, pregnancy and breastfeeding

Please also share this information with your partner if it is appropriate.

Trial medicines can harm an unborn baby or nursing infant. You will not be able to take part in this trial if you are pregnant or breastfeeding. You should also not participate in this trial if you are planning to become pregnant or father a child during the trial or for six (6) months after the completion of trial treatment.

Women of childbearing potential are required to use two highly effective forms of contraception for the duration of the trial (from signing the informed consent form until end of trial treatment) and for three (3) months after the completion of the trial treatment.

Acceptable non-hormonal birth control methods include:

- Intrauterine Device (IUD) PLUS male condom provided coils are copper-banded.
- True abstinence (where the participant refrains from any form of sexual intercourse and is in accordance with the participant’s preferred and usual lifestyle).
- Vasectomised sexual partner PLUS male condom (with participant assurance that the partner has received post-vasectomy confirmation of azoospermia).
- Tubal occlusion PLUS male condom.

Acceptable hormonal birth control methods include:

- Hormonal shot or injection (e.g. Depo-Provera) PLUS male condom.
- Etonogestrel implants (e.g. Implanon, Norplant) PLUS male condom.

- Intrauterine system (IUS) device (e.g. levonorgestrel releasing IUS – Mirena) PLUS male condom.

Men and male partners are also required to use adequate contraception for the entire duration of the trial and for three (3) months after the completion of the trial treatment. This includes:

- Barrier contraception (condom and spermicide) even if female partner(s) are using another method of contraception or are already pregnant (also to protect male partners from exposure to the trial medicines).
- True abstinence (where this is in accordance with the participant's preferred and usual lifestyle).

Male participants should refrain from donating sperm for the duration of the trial and for three (3) months thereafter.

The risks of the trial drugs to the unborn child are currently unknown, so we would like to follow any pregnancies that occur to yourself or partner, during trial treatment or up to three (3) months after you complete trial treatment, up until the birth. This is so that we can learn more about the safety of the trial treatment.

If you or your partner becomes pregnant during the trial or within three (3) months of stopping treatment, you should inform your trial team immediately. Your trial team will discuss all the options available to you. The outcome and progress of any pregnancy will be followed by your trial team, (with your/your partner's consent), and we will ask you questions about the pregnancy and baby, if appropriate.

For Women of Child Bearing Potential: At the bottom of this document is an optional consent section. This is so that you can consent to the collection of data relating to your pregnancy, should a pregnancy occur.

For Male Participants: Should a pregnancy occur to your partner, your partner will be approached separately with information regarding the treatments you have received, and asked whether they would be willing to give consent for their pregnancy to be followed up.

<u>Private insurance policies</u>
You should discuss your participation in this trial with any insurance provider you have (e.g., protection insurance, life insurance, income protection, critical illness cover and private medical insurance) and seek advice if necessary, as failure to notify them may affect or invalidate your cover.

11. What are the side effects of the drug being tested?

Each person's reaction to a new drug is different. Some people have very few side-effects, while others may experience several. You will be closely monitored throughout the trial, but you should inform the trial team if you feel unwell or different in any way. If you have any major concerns or are feeling very unwell please contact your trial doctor immediately using the contact numbers at the end of this information sheet. Informing the trial team about any side-effects will help them manage your care, and they may be able to prescribe medication to ease any side effects. We also need to know so that the potential side-effects of this new combination can be recorded. A break from treatment may be necessary if you are experiencing side effects.

<table>
<tr><th>Side Effect</th><th>Cediranib</th><td rowspan="20">This table displays the side effects noted by the drug or drugs that you will receive.

Very Common side effects typically affect more than 1 in 10 people

Common side effects typically affect less than 1 in 10 people</td></tr>
<tr><td>Bleeding</td><td>Common</td></tr>
<tr><td>Blood clotting problems</td><td>Common</td></tr>
<tr><td>Blood Pressure Changes</td><td>Very Common</td></tr>
<tr><td>Blood Result Abnormalities</td><td>Common</td></tr>
<tr><td>Decreased Appetite</td><td>Very Common</td></tr>
<tr><td>Dehydration</td><td>Common</td></tr>
<tr><td>Diarrhoea</td><td>Very Common</td></tr>
<tr><td>Digestive problems</td><td>Common</td></tr>
<tr><td>Dizziness and/or Sickness</td><td>Very Common</td></tr>
<tr><td>Headache</td><td>Very Common</td></tr>
<tr><td>Hoarse voice</td><td>Very Common</td></tr>
<tr><td>Skin problems (itching, rashes)</td><td>Very Common</td></tr>
<tr><td>Mouth complications (dry or sore)</td><td>Very Common</td></tr>
<tr><td>Pain in fingers & toes</td><td>Common</td></tr>
<tr><td>Thyroid Function Changes</td><td>Very Common</td></tr>
<tr><td>Tiredness and/or Weakness</td><td>Very Common</td></tr>
<tr><td>Urinary Complications (pain or bleeding)</td><td>Common</td></tr>
<tr><td>Urinary Tract Infections</td><td>Common</td></tr>
<tr><td>Protein in Urine</td><td>Very Common</td></tr>
<tr><td>Weight decrease</td><td>Very Common</td><td></td></tr>
</table>

Uncommon side effects

In rarer instances (fewer than 1 in 100 people), other symptoms of taking these drugs have been noted that you should keep in mind before you agree to participate in the WIRE trial. Below these rare side effects are listed for the drug that you may be allocated, should you take part;

Cediranib

- Wound healing complications
- Stroke (arterial thromboembolism- cerebrovascular)

Section 2: Conduct of the trial

12. What if new information becomes available?

Sometimes during the course of a trial, new information becomes available which might affect your decision to continue participating in this trial. Your trial team will contact you to discuss the new information and whether you wish to continue participating in the trial. If you still wish to continue on the trial, we will ask you to sign a new Informed Consent Form.

The trial sponsor, the regulatory authority or the trial doctor may decide to stop the trial at any time. If that happens we will tell you why the trial has been stopped and arrange for appropriate care and treatment for you.

13. What if I decide I no longer wish to participate in the trial?

You can stop participating in this trial at any time without giving a reason and without affecting your future care or medical treatment. If you decide not to participate any further, you will no longer receive the trial drugs. No further tests will be performed on you and no further research samples will be collected. Any data or results already collected will continue to be used in the trial analysis. Your trial team may also choose to withdraw you from the trial if they feel it is in your best interests or if you have been unable to comply with the requirements of the trial. Possible reasons for withdrawing you could include:

- Not taking the trial drugs as required
- Experiencing a serious side effect (your doctor will follow-up regarding your progress until this side effect has stabilised or resolved)
- Your disease gets worse whilst receiving trial treatment
- Finding out that you are not eligible after completing the screening tests
- Being unable to complete the visits or trial documentation as required
- Becoming pregnant or planning to become pregnant
- The trial doctor feels you no longer appear to benefit from the trial drugs.

14. What if there is a problem?

Any complaint about the way you have been dealt with during the trial or any possible harm you might suffer would of course be listened to and addressed wherever appropriate. If you have any concerns about any aspect of this trial you should speak to your trial doctor who will do their best to answer your questions.

In the event that something does go wrong and you are harmed by taking part in the research and this is due to someone's negligence then you may have grounds for a legal action for compensation against Cambridge University Hospitals NHS Foundation Trust or the University of Cambridge. The normal National Health Service complaints mechanisms will still be available to you (if appropriate). The University has obtained insurance which provides no-fault compensation, i.e., for non-negligent harm, you may be entitled to make a claim for this.

If you wish to complain or have any concerns about any aspect of the way you have been approached or treated during this trial, you can do this through the NHS complaints procedure. In the first instance it may be helpful to contact the (to be completed locally as appropriate – in England this will refer to the Patient Advice and Liaison Service (PALS)) at your hospital.

15. Will my taking part in this trial be kept confidential?

Cambridge University Hospitals NHS Foundation Trust (CUH) and The University of Cambridge are the Sponsors for this clinical trial based in the United Kingdom. They will be using information from you and your medical records in order to undertake this trial and will act as the data controller for this trial. This means that they are responsible for looking after your information and using it properly. The Sponsor organisations will keep identifiable information about you for 5 years after the trial has finished to ensure your safety and allow the trial to be reviewed by the authorities after it is finished.

Your rights to access, to change or to move your information are limited, as the Sponsor organisations need to manage your information in specific ways in order for the research to be reliable and accurate. To safeguard your rights, we will use the minimum personally-identifiable information possible.

You can find out more about how the Sponsor (s) use(s) your information using the information below:

- For Cambridge University Hospitals NHS Foundation Trust, please visit: https://www.cuh.nhs.uk/corporate-information/about-us/our-responsibilities/looking-after-your-information

Or email the Data Protection Officer at: gdpr.enquiries@addenbrookes.nhs.uk

- For University of Cambridge, please visit:
https://www.medschl.cam.ac.uk/research/information-governance
Or email the Information Governance team at: researchgovernance@medschl.cam.ac.uk

For participants recruited at CUH (where the Sponsor is also the site):
Cambridge University Hospitals will collect your name, NHS number and contact details to contact you about this trial and make sure that relevant information about the trial is recorded for your care and to oversee the quality of the trial. Individuals from the Sponsors and regulatory organisations may look at your medical and research records to check the accuracy of this trial. Cambridge University Hospitals will pass these details to the Sponsors along with the information collected from you and/or your medical records. The only people in the Sponsor organisations who will have access to information that identifies you will be people who need to contact you in relation to this trial and to audit the data collection process. Cambridge University Hospitals will keep identifiable information about you from this trial for *5* years after the trial has finished.

For participants recruited at other participating sites:
(Add site name) will keep your name, NHS number and contact details to contact you about this trial, and make sure that relevant information about the trial is recorded for your care, and to oversee the quality of the trial. Certain individuals from the Sponsors, and regulatory organisations may look at your medical and research records to check the accuracy of this trial. The Sponsor(s) will only receive information without any identifying information. **(Add site name)** will keep identifiable information about you from this trial for *XX* years after the trial has finished.

All information collected about you as a result of your participation in the trial will be kept strictly confidential. Your personal and medical information will be kept in a secured file and be treated in the strictest confidence.

Once you have agreed to participate in this trial you will be allocated a Trial ID Number. This is a unique trial number which will be used on all your trial documentation along with your date of birth. Your date of birth is considered to be personal information. We collect this personal information on trial documentation to help ensure that the data we receive as part of your trial participation is correctly allocated to you. By cross checking these two unique references we can ensure the integrity of the data.

When you agree to take part in this trial, the information about your health and care may be provided to researchers running other research studies in this organisation and in other organisations. These organisations may be universities, NHS organisations or companies involved in health and care research. Any serious side effects that occur during the trial will also be shared to the manufacturer of the trial drugs (AstraZeneca) using your trial-specific identifiers. The statistics for this trial is being undertaken by the University of Newcastle. All data that is shared with the University of Newcastle will be identified using only your trial ID and date of birth.

Your information will only be used for the purpose of health and care research, and cannot be used to contact you or to affect your care. It will not be used to make decisions about future services available to you, such as insurance.

Your coded trial data may be sent to other country(ies) outside the European Economic Area (EEA) for analyses, where the data protection laws are not the same. Your coded personal data may also be shared with off-site licenced laboratories for the purpose of analysing samples collected from you in this trial. However this information will not identify you and will not be combined with other information in a way that could identify you. The people who analyse the information will not be able to identify you and will not be able to find out your name, NHS number or contact details. Only anonymous trial data, without any personal information will be published at the end of the trial.

We will need to inform your GP of your participation in this trial. This is so that any medical decisions that your GP makes will take into account the fact that you are receiving drugs as part of this trial.

16. What will happen to my samples and scan images?

With your permission, we will analyse the blood, urine, kidney and tumour samples collected from you during this trial. This analysis will take place over a long period of time, so any results obtained will not alter your care pathway.

If you decide to stop taking part in the trial please tell the trial doctor if you want to change your mind about us using your donated sample(s) to look at the molecular and genetic makeup of the cancer.

Routine blood samples

Blood samples taken will be analysed immediately by your hospital and will be destroyed once the laboratory tests are complete. The results will include those you would receive as part of your standard care pathway, plus any results required for the trial. These results will be accessible to your trial team.

Research samples

Research samples taken will be labelled with your Trial ID Number, processed and stored initially by your trial team at your hospital, before being transferred to an approved facility for analyses. The approved facilities are either part of the Cambridge Biomedical Campus or AstraZeneca. These labs may be inside or outside of the UK. The samples will be securely stored and analysed by specialist teams. You will not be identifiable to researchers from any of the laboratories we may use for research analysis.

These samples will be analysed in a way which will tell us whether WIRE trial treatments are eliciting the biological response that we hope and expect to see. There may be other tests that we perform on your research samples to give us additional information regarding the drug activity.

Once the research analysis is complete any remaining samples will either be:

- Stored by the research team in line with the relevant regulatory requirements, in an appropriate facility pending ethical approval for use in a future trial outside of the WIRE trial.
- Will be disposed of in accordance with the UK Human Tissue Authority code of practice.

Archival tumour

You may have previously had a biopsy of your kidney, and therefore have more than one tumour sample. Not all patients will have undergone a previous biopsy. If you have however, we will ask your permission to access this 'Archival' tissue from your local hospital, so that we can compare the changes with your recent biopsy sample. This will also help us in the event that your most recent sample isn't big enough for us to perform our analysis. Granting us permission to access these samples is entirely optional.

Genetic Testing (All samples)

All of our cells in our body get information on how to work and function through a molecule called deoxyribonucleic acid (DNA). DNA is a record of instructions where the instructions are called genes. Genetic testing looks at your genes and could either look at one or some specific genes or all of your genes – your whole DNA. Genes affect how we grow and develop. Nobody else has exactly the same genes as you do, unless you have an identical twin. These differences mean that some people are more likely than others to get certain diseases and they also mean that medicines affect people differently. Some genes can be important in more than one disease.

Genetic testing in this trial is only for research purposes, but is an essential part of the trial. If you do not want to agree it to, you will be unable to take part. These genetic tests will be performed on any samples you donate to the trial, and requires no additional time from you.

The trial team may also undertake laboratory tests using your samples that reenact the environment of natural body conditions in a laboratory setting - so called '*ex vivo* modelling'.

This method of analysis typically provides very meaningful data regarding how cells interact naturally with the drugs received. Tissue cells are grown in a laboratory environment and could be modified to implant in mice with low resistance to disease. The laboratory staff will monitor the growth of the tumour (derived from your own) within the mice. Any laboratory performing this analysis would have the appropriate license for testing on mice. You have the right to take part in the trial without these particular lab procedures taking place. If you give your optional consent for these procedures, you may withdraw your consent for your samples to be used in this way at any point until this laboratory analysis begins.

Cambridge University Hospitals NHS Foundation Trust & the University of Cambridge may do this testing in its own laboratories or it may be done in the laboratories of other organisations (such as universities, collaborators and genetic specialist), or both. Results from this research may be shared and used as described in 'Will my taking part in this trial remain confidential?' In addition Cambridge University Hospitals NHS Foundation Trust & the University of Cambridge, and its designated organisations may conduct future research where they will share summary results (not your individual results) or anonymous datasets with other researchers, from other companies or universities, for example. The results may be combined with the results of other studies, for example as part of scientific databases. The researchers can only use this information for medical and healthcare related research.

Radiological (Scan) Images

The images obtained by way of CT and/or MRI scans will be coded, using only your trial ID number and Date of Birth. These coded images will then be sent on a secure CD, through the post, to the Sponsors (Cambridge University Hospitals and the University of Cambridge). These images will be stored on password-protected computers at Cambridge University for the duration of the trial.

CT scans will be used by your doctor to monitor general size changes to the tumour, and will likely be kept at your local hospital, but the central trial team may request them for checking of the measurements provided by your hospital. The people performing the analysis on your MRI images will not be able to identify you. They will be experienced Radiologists that will be able to identify areas of the cancer to get a general idea of how the cancer is behaving; so called 'Regions of Interest'. By using specialist software, the Radiologists can measure the amount of blood flowing through the cancer. This analysis will be part of the assessment of your MRI scan performed before your treatment, and after your treatment for comparison.

The anonymised images may support future research projects, for example finding new ways of gaining even more insight into renal cancer. Therefore, we ask for your permission to use these images, which do not identify you personally, in other future research projects.

17. What will happen to the results of the trial?

The results of the trial will be anonymous and you will not be able to be identified from any of the data produced. When the results of this trial are available they may be published in peer reviewed medical journals and used for medical presentations and conferences. They will also be published on the EU Clinical Trials Register website, a central registry for all clinical trials conducted in the EU.

Anonymised datasets from the trial will be made available to the funder (AstraZeneca), and may also be made available to other researchers in line with national and international data transparency initiatives.

If you would like to obtain a copy of the published results please contact your trial doctor directly who will be able to arrange this for you.

18. Who is funding the trial?

The trial is being funded by AstraZeneca and the Cancer Research UK Cambridge Centre. AstraZeneca is also supporting this trial by providing the trial drugs free of charge.

The trial is being managed by the Cambridge Clinical Trials Unit – Cancer Theme. The Chief Investigator is Mr Grant Stewart who is a University Lecturer at the University of Cambridge and Honorary Consultant Urological Surgeon at CUH.

19. Who has reviewed this trial?

All research within the NHS is reviewed by an independent group of people called a Research Ethics Committee, to protect your interests. This trial has been reviewed and given favourable opinion by the (name of REC here). The Medicines and Healthcare Products Regulatory Agency (MHRA) who are responsible for regulating medicines in the UK have also reviewed this trial.

20. Further information and contact details

If you have any questions concerning this trial please contact your:

Trial Doctor
Name: [INSERT your hospital contact details here]
Tel: [INSERT your hospital contact details here]
Research Nurse
Name: [INSERT your hospital contact details here]
Tel: [INSERT your hospital contact details here]

In the event of an emergency please contact:

24-hour contact details
Contact: [INSERT your hospital contact details here]
Tel: [INSERT your hospital contact details here]

Outside of an emergency,
You may wish to contact one of the following organisations that are independent of the hospital at which you are being treated:

CancerHelp UK is a registered charity providing information about all aspects of cancer. It can provide useful information on cancer treatments and medical research. You can contact their nurses on freephone 0808 800 4040. You can also access their web site at: www.cancerresearchuk.org

MACMILLAN Cancer Support is a registered charity providing information about all aspects of cancer. They have published several useful booklets on different types of cancer, chemotherapy, radiotherapy, and clinical trials in general. You can contact the nurses on freephone 0808 808 0000. You can also access their website at www.macmillan.org.uk

If you wish to complain or have any concerns about any aspect of the way you have been approached or treated during this trial, you can do this through the NHS complaints procedure. In the first instance it may be helpful to contact the Patient Advice and Liaison Service (PALS) at your hospital.

Patient Advice and Liaison Service (PALS) at:
Hospital Name: [INSERT your hospital PALS contact details here]
Tel: [INSERT your hospital PALS contact details here]

TO BE PRINTED ON HEADED PAPER

UNIVERSITY OF CAMBRIDGE

Cambridge University Hospitals NHS
NHS Foundation Trust

INFORMED CONSENT FORM

Trial Title: WIRE: WIndow-of-opportunity clinical trials platform for evaluation of novel treatments strategies in **RE**nal cell cancer.

Principal Investigator: [INSERT your PI name here]

Participant Number: ___________ (please add once the participant is registered)

**If you agree with each sentence below, please initial the box**		**INITIALS**
1	I have read and understood the WIRE Group 1, Cediranib only, Participant Information Sheet version 3.0, dated 10th February 2020 for the above trial and I confirm that the trial procedures and information have been explained to me. I have had the opportunity to ask questions and I am satisfied with the answers and explanations provided.	
2	I understand that my participation in this trial is voluntary and that I am free to withdraw at any time, without giving a reason and without my medical care or legal rights being affected. I also understand that data or samples already collected will continue to be used in the trial analysis.	
3	I understand that personal information about me will be collected and used in accordance with this information sheet. This information will be kept in the strictest confidence and none of my personal data will be published.	
4	I understand that sections of my medical notes or information related directly to my participation in this trial may be looked at by responsible individuals from the sponsor, regulatory authorities, research personnel where it is relevant to my taking part in research and that they will keep my personal information confidential. I give permission for these individuals to have access to my records.	
5	I understand that my GP will be informed of my participation in this trial and sent details of the WIRE trial.	
6	I have read and understood the compensation arrangements for this trial as specified in the Participant Information Sheet.	
7	I understand that the team/doctors in charge of this trial may close the trial or stop my participation in it at any time without my consent.	
8	I have read and understood my responsibilities for the trial including using appropriate contraception as listed in section 6.	
9	I agree to give blood and urine for research related to this trial.	
10	I agree to give tumour and healthy tissue samples for research related to this trial	
11	I give my permission to allow my kidney tissue and research blood, urine & tumour samples to be sent to specialist teams on the Cambridge Biomedical Campus, or to laboratories contracted and approved by the Sponsor(s) for tests/analysis related to this trial. I understand that only the minimum information will be supplied and that I will not be identifiable to any of the researchers.	
12	I understand that the results from the analysis carried out on the research blood and tumour samples will not be fed back to me.	
13	I understand that the information collected about me will be used to support other research in the future, and may be shared anonymously with other academic and commercial researchers external to the project, within the UK and beyond	

14	I give my permission to allow for DNA testing of my samples		
**OPTIONAL:** Please read each statement below and initial the relevant box		**INITIALS**	
		Yes	No
15	I give my permission to allow for any archival tumour tissue to be collected for tests related to this trial. I understand that only the minimum information will be supplied and that I will not be identifiable to any of the researchers.		
16	I give my consent for my tissue samples to be used to grow cell cultures or be implanted into mice		
17	I give my permission to allow any remaining research blood and tumour samples taken as part of this trial to be retained in an HTA licenced facility pending Ethical Approval for use in another project at the discretion of the Chief Investigator. I understand that these samples will be retained in an approved storage facility, that only the minimum information will be supplied and, that I will not be identifiable.		
18	I give my permission for my anonymised radiological images to be used as part of the data for future research studies		
**FOR WOMEN OF CHILDBEARING POTENTIAL ONLY**<br>**OPTIONAL:** Please read each statement below and initial the relevant box		**INITIALS**	
		Yes	No
19	If I become pregnant during, or in the three (3) months after receiving the trial drugs, I agree to information being collected about me, my pregnancy and my baby.		
20	I understand that sections of my medical notes or information related directly to my pregnancy may be looked at by responsible individuals from the sponsor, regulatory authorities and research personnel where it is relevant to my taking part in research. I give permission for these individuals to have access to my records.		
21	I agree to give my pregnancy information voluntarily and understand that I am free to withdraw at any time without giving a reason and without my medical care or legal rights being affected. I understand that all data collected up to the withdrawal of consent will be kept confidential.		

I agree to participate in this trial:

**Name of participant**	**Signature**	**Date**
**Name of person taking consent**	**Signature**	**Date**

Time of consent (24hr clock): _______:_______

1 copy for the participant, 1 copy for the trial team, 1 copy to be retained for hospital records

TO BE PRINTED ON HEADED PAPER

UNIVERSITY OF CAMBRIDGE

Cambridge University Hospitals NHS Foundation Trust

WIRE

NOVEL TREATMENTS IN RENAL CELL CANCER

Group 2- Cediranib and Olaparib Combination

PARTICIPANT INFORMATION SHEET & INFORMED CONSENT FORM

WIRE: WIndow-of-opportunity clinical trials platform for evaluation of novel treatment strategies in **RE**nal cell cancer.

You are being invited to take part in a research trial. Before deciding whether to take part, you need to understand why this research is being done and what it involves.

Please take time to read the following information carefully and talk to others about the trial if you wish. Please ask your trial team if anything is not clear or if you would like more information. Please take time to decide whether or not you wish to take part. You will be receiving this Participant Information Sheet before any diagnosis has been made i.e. it is not yet confirmed that you have renal cell cancer.

Section 1 tells you the purpose of this trial and how the trial would involve you, including a description of what the various tests are, and some of the risks and restrictions of taking part

Section 2 gives you more detailed information about the trial process, including information about how the trial is run, and how we may use your data.

Section 1: Purpose of the trial and what will happen

1. What is the purpose of the trial?

Kidney cancer is the 7th most common cancer in the UK. Currently many patients with this cancer are offered a kidney removal operation (known as nephrectomy), which cures many patients with no further need for treatment. However, some may receive drug treatment after surgery.

WIRE is a trial that will administer drugs to patients in the time between the decision to operate and the nephrectomy operation. Patients are not usually given anti-cancer drugs during this period, and indeed may never need them, but it could give researchers important information to guide the best combinations of anti-cancer drugs to prioritise for later phase clinical trials. The trial will investigate the effect and safety of 3 different cancer drugs, individually or in combination, in patients with a specific type kidney cancer called clear cell renal cell cancer. The cancer may be localised (within the kidney) or metastatic (it has spread outside of the kidney).

2. What are the drugs being tested?

There are 3 drugs being used in this trial, Cediranib, Olaparib and Durvalumab. All three of these drugs target different parts of cancerous cells which might decrease the size of the tumour over a period of time. However, the duration of treatment in this trial is very short in comparison to how long these drugs would normally be given for. Therefore, it is unlikely that the trial treatment will noticeably reduce the tumour size if it is confirmed that you do have kidney cancer. In this trial, we will primarily be looking at cancer's response to these drugs by taking a closer look at the tumour cells in the laboratory and by using new imaging technology.

Although the trial uses 3 drugs, we will only be giving you one or two of these drugs. The drugs you are given will depend on the group you are allocated. The trial team already know which group you will be allocated. Currently, we are recruiting participants for the **Cediranib and Olaparib** group. Each group has its own specific entry criteria which may involve different tests and assessments; this is discussed further under *Section 4 Do I have to take part?*

Cediranib is a targeted cancer therapy drug called a 'growth factor blocker'. It binds to a specific protein on the surface of the cancer cells which controls blood vessels' development (vascular endothelial growth factor (VEGF)). By blocking the usual action of this protein cediranib may reduce blood supply to the tumour and may slow down the growth and spread of the cancer. Cediranib is being tested in clinical trials, but is not currently licenced in the EU. This drug is an oral tablet (a tablet taken by mouth) which will be taken for at least 14 days in total, and until 36 hours before your surgery.

Olaparib is also a cancer therapy drug called a 'PARP Inhibitor'. PARP is a protein which helps damaged cells to repair themselves. "PARP Inhibitors" block the ability of damaged (cancer) cells to repair themselves. Olaparib is being tested in clinical trials, but is currently only licenced in the EU for use in patients with ovarian and breast cancers. This drug is an oral tablet (a tablet taken by mouth), which will be taken for at least 14 days in total, and until the morning of your surgery.

3. Why have I been invited?

You have been invited to participate in this trial because you may have renal cell cancer (kidney cancer) that may not be cured with surgery alone. The diagnosis of renal cell cancer will be confirmed or disproved with a pre-surgery biopsy. In normal care, usually your treating doctor will recommend proceeding with a nephrectomy or partial nephrectomy (removal or partial removal of the kidney) without the need of a pre-surgery biopsy. However, as part of the WIRE trial, participants will be required to undergo a biopsy during the screening stage to determine whether they have the type of cancer we are investigating.
We plan to include up to 76 patients with kidney cancer from approximately 2 hospitals across the UK.

4. Do I have to take part?

Participating in this trial is completely voluntary. If you decide to participate you will be asked to sign an Informed Consent Form. You are however still free to change your mind and leave the trial at any time and without giving a reason. If you chose not to participate or to leave the trial, your current and future medical treatment will not be affected in any way. Your normal standard care will not be affected in any way.

You should also be aware that entry into this trial requires you to meet some strict entry criteria that your doctor will assess you for over the next few weeks, during a period called 'screening'. At the moment we will not know whether you are eligible for the trial as there are

further tests and assessments to do, but some people who consent to take part in this trial may not meet the entry criteria to receive the trial drugs.

Before proceeding, it is important to understand that you may not benefit from the extra drugs and procedures involved in this trial. Surgery alone may be sufficient to cure the cancer alone, although there is no way to predict this for certain. The possible side effects could potentially delay your surgery, and participation in the trial could make you feel fatigued and nauseous. However, your doctors will be monitoring how you are feeling very closely, making every attempt to minimise any side effects.

It is also important you are aware that based on previous research the average chance of being cured by surgery alone needing no further treatments is 42%, in whom no further treatments would be needed. It is also important that you are aware that you will not have access to the trial drugs after your operation, and you might not be permitted to take part in other clinical trials if you decide to participate in the WIRE trial.

5. Expenses and payment

You will not receive any payment for participating in this trial however we can reimburse reasonable travel and parking costs incurred by your participation in this trial. This is up to a maximum of £180 for the entire duration of your participation in this trial. We will ask you to retain your receipts wherever possible.

6. What are the possible advantages and disadvantages of taking part?

It is unlikely that you will benefit clinically from taking part in this trial, although it is possible that the tumour may shrink very slightly. It is also hoped that taking the medications used in the trial could provide you with a better outcome, but this cannot be directly measured in this trial. You will gain access to drugs that are only available within a clinical trial. You are likely to have more contact and for longer with your care team than if you were not involved in a trial, and some patients gain reassurance from this. It is hoped that information collected as part of your participation in this trial will benefit patients with kidney cancer in the future and you may contribute to their future treatment and care. Your care team will be able to share information with you regarding how the tumour cells responded and/or any tumour size reduction, if you wish to know this.

In addition to the above benefits there are also aspects of your participation you may find difficult. For instance, if you decide to take part:

- You will need to undergo a biopsy at the screening stage which is likely to require a full day at the hospital. It is also likely that this biopsy would not have been required if you were not participating in this trial. This is an extra procedure for about 70% of patients. You can find more information about this under section 9 – Tumour biopsy on page 12.
- There will be an increased number of hospital visits due to participating in the trial. You will be required to come to the hospital an extra 4-5 times during the trial. These visits are to conduct initial assessments and scans (1 extra visit); clinic visits whilst receiving the trial drug(s) (2-3 extra visits); a visit for the scan before surgery (1 extra visit). The average length of time per visit is about 2 or 3 hours, but actual time may vary. The schedule and timing of these visits is available on page 9.
- You will undergo at least an additional 2 MRI scans if you decide to take part in this trial, with potentially another 2 MRI scans using a new type of scan called a 'hyperpolarised MRI' if available at your hospital (see section 9, pages 11 and 12

for more information). An additional 1-2 CT scans may be required as recommended by the doctor based on information from your diagnosis and previous scans. You would not have normally received these scans if you were not participating on the trial.

- You could experience drug side effects such as nausea, fatigue, diarrhoea, dizziness, decreased appetite and blood test abnormalities. A full list of known side effects is listed on pages 15 and 16. It is also possible that some of these side effects might delay your surgery.
- There will be 6 additional tissue biopsies (samples) taken during your surgery, this will likely only add a few minutes to your standard surgical procedure. Whilst these are unlikely to impact you or prolong your stay in hospital, it is important that you are aware these are taken. More information about these tissue samples is mentioned on page 13 and 14.

  The drugs you will be taking as part of this trial will not be given to you after the surgery, and may prevent you from taking part in other clinical trials after your surgery.

7. What are the alternatives for treatment?

The standard care for you is a nephrectomy (removal of the kidney) or partial nephrectomy (removal of the tumour only) without any drug treatment before surgery.

8. What happens when the trial stops?

Following completion of the trial you will not be able to receive any further trial drug treatment and we will refer you back to your primary consultant. You will still be able to contact your trial Nurse or Practitioner, however, with any questions about the trial.

9. What will happen to me if I take part?

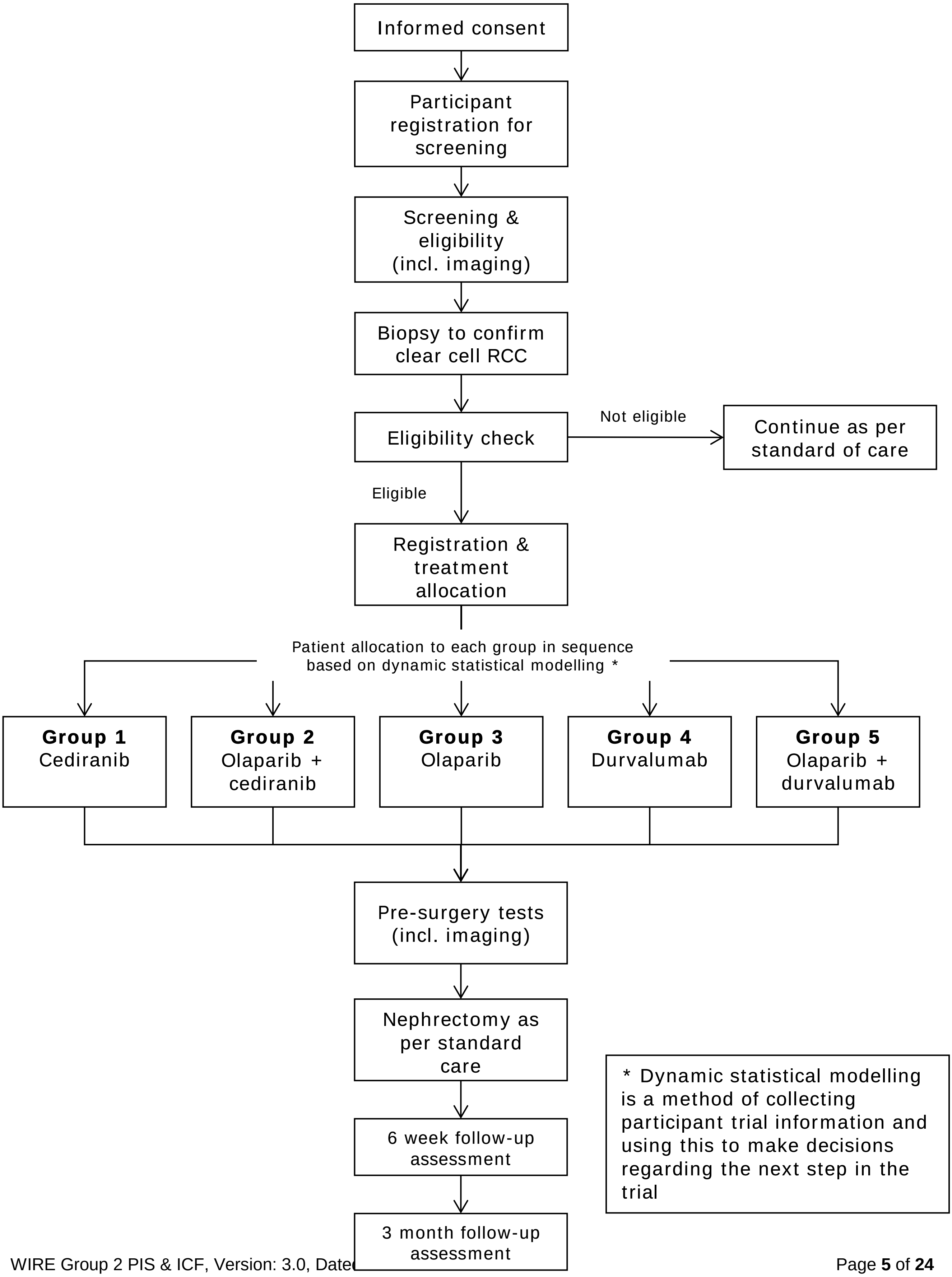

Hospital Appointments

Informed Consent

If you agree to participate in the trial, you will be invited to sign the Informed Consent Form at the end of this document. You will be given a copy of this to take away and refer to later.

Screening and baseline

Standard of care visit plus extra trial assessments 5-6 hours in duration. Screening may require an additional visit to complete all assessments, dependent on hospital capacity.
To check that you meet all of the requirements for entering this trial you will need to have some tests. This is called the “screening period”. You will be given appointments to see the trial team for these tests, which will be carried out within a 28 day period. This may include more than one visit to the hospital. All of the tests listed during this period and the following visits are further explained later in this information sheet (see page 5). All of the tests listed under Screening will collect data for the research trial.

- A check of your weight, blood pressure and temperature
- A full physical examination
- Clinical review
- Performance status check
- Blood and urine sampling
- 3 Electrocardiogram assessments (ECGs), 5 minutes apart.
- A minimum of one type of scan, including a Dynamic Contrast Enhanced Magnetic Resonance Imaging (DCE-MRI) scan at least (a type of scan that uses strong magnetic fields and radio waves to produce detailed images of the body and organs). You may undergo a hyperpolarised MRI scan (a new MRI scanning technique which produces extremely detailed images of the body and organs), depending on the group you are allocated to, and the treatment centre you are attending. You may also undergo a CT scan, depending on how long ago your last CT scan took place (the risks of increased radiation exposure are explained further on in this document, in the ‘Tests and Assessments’ section).
- Collection of your Medical History
- Research blood and urine sampling- we will take these samples before you have the tumour biopsy, on the same day
- Tumour Biopsy
- Pregnancy test (female participants only)

Your weight, blood pressure, temperature, physical examination, performance status check, blood & urine samples, CT and ECGs may be assessed as part of your standard care pathway (these would usually take place without trial participation anyway as part of the usual pre-surgery checks). A tumour biopsy would not normally take place in about 70% of patients, but all participants will have a biopsy in order to be eligible for the trial.

Additional ECGs, a possible repeat CT scan (if you haven’t had one within the last 28 days) and all of the other assessments and scans listed are extra for the trial.

Registration

Some of the tests performed during Screening require the results to fall within certain trial ranges. If any of your results do not fall within these ranges, you will not be able to participate in this trial and will be referred to follow the usual treatment pathway for patients with your condition. However, if the screening tests show that you are suitable to participate in this trial, your clinical team will register you into the trial.

There are 5 different treatment groups in this trial, but each participant will only be allocated to one of these groups, and given the appropriate information sheet for the drug that they might receive. If you decide to participate and meet the entry criteria, you will be allocated to Group 2 and be given Cediranib & Olaparib

.

Clinic Visits

Extra visits for the trial, up to 3 hours per visit in duration

Once your participation in this trial has been confirmed we will ask you to attend regular clinic appointments to see the trial team whilst you are receiving the trial drug/s and we will perform a number of trial related tests to ensure you are tolerating the trial drugs well. Initially these will be performed on the 1st and approximately 15th day of your treatment but will be flexible around weekends and bank holidays. You will need to have fasted for 6 hours before the Research urine tests, and your research/care team will advise you regarding the timescales on this. Both of these visits are extra visits for the trial and all assessments for these visits below are needed just for the trial and are outside of standard care:

- A weight, blood pressure and temperature check
- A physical examination (if your doctor believes it is necessary)
- Performance status check
- Clinical review
- At least 1 electrocardiogram assessment (ECG). Based on your results, your trial team may ask you to perform 2 additional ECGs, all 3 taken 5 minutes apart.
- Blood and urine assessments. Pre-treatment (Day 1)
- Pregnancy test (female participants only)
- Dispensing of your tablets to take home, and a diary to record when you take them (at Day 1 only).

In addition to the assessments above, the following tests will be performed on Day 15 ONLY

- Research blood and urine sampling
- Assessment of any side effects from the medication
- Check that you have enough and are correctly taking, your medication

Your clinical trials team may decide to give you an additional assessment on approximately the 22nd day of treatment, if you are still taking the trial drug. This may be by telephone or in clinic; this day 22 assessment will be an extra assessment for the trial, and the below tests will be extra assessments for the trial, outside of standard of care:

- Performance status check
- Assessment of any side effects from the medication
- Check that you have enough and are correctly taking, your medication
- Clinical review

If you experience unmanageable side effects, or if your trial doctor thinks you should not continue, the trial drugs will be stopped and you will return to the normal standard of care provided by the hospital for the treatment of the cancer.

Pre-surgery

Extra visit, up to 6 hours in duration

Within 3 days of your surgery date, you will undergo at least one scan to assess whether the medication you have been taking is affecting the tumour. These are all extra assessments for the trial, outside of standard care, and the visit could take most of a day or more than one day:

- DCE-MRI
- Hyperpolarised MRI (depending on the group you are allocated to, and the treatment centre you are attending)
- CT scan (if the cancer has spread outside the kidney- your Trial Nurse or Practitioner will advise you)
- Research blood and urine sampling (these are outside of standard care and are required for the trial only)
- Routine blood sampling

Surgery

Standard of care visit. Surgical prep plus 3 hours surgery time.

During your nephrectomy, we will obtain tumour samples, as well as samples of the normal kidney tissue next to the tumour. The surgery is standard care but the amount of samples taken will be higher than in standard care.

Follow-up – 6 weeks after surgery

Standard of care visit, up to 2 hours in duration

You will be given an appointment to see the trial team approximately 6 weeks after surgery to ensure that you have recovered from any side effects. This 6 week check is usual for patients who have undergone nephrectomy, and is standard care. During this visit you will have:

- Performance status check
- Clinical review
- Routine Blood sampling
- Pregnancy Test

End of trial follow-up – 3 months after surgery

Standard of care visit, up to 2 hours in duration

You will be given another appointment to see the trial team approximately 3 months after surgery to ensure that you have tolerated the trial drugs well. A CT scan will be performed prior to this visit. Following this visit, you will not need to attend any further trial related visits. This 3 month check is usual for patients who have undergone nephrectomy, and is standard care. During this visit you will have:

- Performance status check
- Clinical review
- Routine blood sampling
- Research blood and urine sampling (these are outside of standard care and are required for the trial only)
- Pregnancy test

Tests and Assessments	Screening (up to 28 days prior to Day 1 of treatment)	Treatment phase			Pre-surgery	Surgery	Follow-up	
		**Day** 1 (± 5 days)	**Day 15** (± 5 days)	**Day 22 (Optional)** ± 5 days			6 weeks after surgery ± 7 days	3 months after surgery ± 14 days
Consent	X							
Medical history	X							
Vital signs	X	X	X					
Performance status check	X	X	X	X			X	X
Clinical review	X	X	X	X			X	X
Physical examination	X	If clinically necessary	If clinically necessary					
ECG	X	X	X					
Urine sample	X	X	X				X	X
Blood for routine tests	X	X	X		X		X	X
Pregnancy test	X	X	X					X
Post-menopausal test	X							
Blood for research tests	X		X		X			X
Urine for research tests	X		X		X			X
DCE-MRI	X				X			
Hyperpolarised MRI	If needed				If needed			
CT scan (*your doctor will tell you if needed)	X*				If needed*			X
Tumour biopsy (screening)	X							
Cediranib & Olaparib Drug dispensing		X						
Cediranib & Olaparib Drug compliance check			X	X				
Tissue samples (research)						X		
Full or partial nephrectomy						X		
Total time of visit	5-6 hours over 2-3 visits	2-3 hours over 1 visit	2-3 hours over 1 visit	1-2 hours over 1 visit or 20 min phone call	4-6 hours over 1-2 visits	2-3 hours of surgery	1-2 hours over 1 visit	1-2 hours over 1 visit

What will happen and when?

Tests & Assessments

You will be monitored carefully by the trial team whilst you are on this trial. Most of the tests in this trial are necessary to give you the trial drugs safely and ensure you are tolerating the trial drugs well. These tests are explained below, including the possible **risks and/or benefits** of each.

Medical history
Your medical history will be reviewed by the trial team during the screening period. This will involve a discussion about your past and present health, lifestyle and any symptoms you may currently have. This is so that the trial team are aware of any pre-existing conditions. The trial team will also record your gender, ethnicity and date of birth. This is to collect demographic data for the trial.

Weight, blood pressure and temperature
The trial team will monitor your blood pressure and temperature in order to properly assess the side effects to your treatment. These indicators will help the doctors to properly diagnose you and to manage your care.

Clinical review
The trial team will regularly discuss and review your general wellbeing, discuss any symptoms and/or side-effects that you may have, and record any medications you may be taking. This is so that the trial team has a full picture regarding your current state of health.

Your trial team may end your participation in this trial if you have severe or unmanageable side-effects to the trial drug/s. If this happens your trial team will follow-up with you regarding your progress until the side effect has stabilised or resolved.

Physical examination
Your trial doctor will perform a physical examination during the screening period and whilst you are receiving the trial drugs. This will ensure that the trial team has a full picture regarding your current state of health.

Drug Compliance Check
It is important that you take the trial drugs as directed by your trial team. We will provide you with a diary to log the medication that you are taking as part of this trial (cediranib and olaparib). You must also return any tablets or the empty container to the trial team at the end of your treatment and bring your diary with you to each visit.

Electrocardiogram (ECG)
You will be asked to have several ECGs as part of this trial during the screening period and whilst you are receiving the trial drugs. These record an electrical trace of your heart rhythm and are required to confirm that you are fit enough to receive the trial drugs. They will also monitor the health of your heart during the trial. We will ask you to lie down whilst a series of leads are placed on your chest area, wrists and ankles with adhesive pads, these will record a trace of your heart rhythm. This takes a few minutes and is painless. This is to check the health of your heart.

Echocardiogram (ECHO)
This assessment will ONLY be performed at screening if your trial clinician thinks it necessary, and ONLY if you are on one of the cediranib groups of the trial. This is to check the function of your heart. There may be repeat scans during the trial to check on your heart's function.

Performance Status check
Your performance status check will track the impact that your condition or treatment has on your day-to-day activities and on your “usual” level of day-to-day functioning. This is assessed by asking you questions about your level of activity around the house or at work (as applicable).

Pregnancy test/ Non-childbearing status (female participants only)
If you are female and of childbearing potential you will be asked to give a blood sample during the screening period **to confirm that you are not pregnant before you can be enrolled into this trial.** You will also have several pregnancy tests during the trial. If you are of non-childbearing potential you may be asked to provide a sample of blood during the screening period to confirm your non-childbearing status. This is because we do not yet know the effect of the trial drugs on a developing baby (foetus). This is an extra sample but can be taken from the same bottle as the other blood tests. Most female patients would normally have a urine test.

MRI Scans
You will undergo two DCE-MRI scans if you participate in this trial, and may also have two hyperpolarised MRI scans. It is important that you are aware that the MRI scans would not have been required if you were not participating on this trial. You may need an injection of a contrast agent to improve the quality of the scan. The injection is usually given in a vein in your elbow (the same place as a blood test), which may cause discomfort and/or bruising. When you have the scan, you will be asked to lie still on a couch which will slide into a metal tunnel, which some people find this uncomfortable and/or claustrophobic. The scan lasts for about 45-60 minutes. You will also be able to talk to the radiographer whilst you are having your scan, or listen to music if you prefer. If you are anxious in enclosed spaces you may be prescribed a tablet to help you relax before you have the scan.

DCE-MRI scan
We will ask you to have a DCE-MRI scan during the screening period and before your surgery. DCE-MRI is a type of scan that uses strong magnetic fields and radio waves to produce detailed images of the body and organs. We do this to establish how the trial drugs affect blood flow in the tumour and how the cancer is responding to the trial drugs.
It is known that small amounts of these contrast agents may remain in the brain after a scan with these agents, although there is currently no evidence that these small amounts cause any harm and these agents have been used for many decades. These contrast agents are routinely given during a kidney cancer MRI so we can have a clear understanding of the blood supply to the cancer. You may experience some side effects such as nausea, headaches and dizziness which are very common side effects. Other side effects such as vomiting, injection site reaction, laboured breathing, and allergic reactions are less common. You should tell a staff member immediately if you experience any of these symptoms so you can be treated promptly. We will use the lowest dose of the contrast agent required for a clear image and if you have any questions about your scan, please speak to your doctor.

Hyperpolarised MRI
You may also be asked to have hyperpolarised MRI scans during the screening period and before your surgery. These scans will enable researchers to assess the cancer tumours and the effectiveness of trial drugs. Not every participant will receive this scan, depending on the numbers already in the trial and local availability.

The contrast agent to be injected is a breakdown product of sugar called pyruvate. Although hyperpolarized carbon MRI is a new technology, tests to date have demonstrated no significant safety issues. Pyruvate is a naturally-occurring molecule in the body. Rarely, people who have had an injection of pyruvate in previous studies developed: unusual taste within the mouth, headaches, flushing, diarrhoea or dizziness after the injection. These side effects were mild and short lasting. Although it is unlikely that an allergic reaction or other side effect will occur, there are facilities in place within the MRI unit, and within the hospital, to manage these appropriately. If you have any questions about your scan, please speak to your doctor.

Computerised Tomography (CT) scan

You may have a CT scan during the screening period (depending on how recent your last one was) and, if required, approximately 3 days before your surgery (you will be told in advance if this includes you). You will also have one just before your 3 month follow up visit. CT scans are used to take pictures of, measure and assess cancer tumours.

When you have the scan, we will ask you to lie still on a couch which will slide through the scanning machine. The scan lasts about 30 minutes, sometimes longer. You may need to drink or have an injection of a contrast agent into a vein which improves the quality of the scan images. You may however be observed for some time after the CT scan to check for a reaction to the contrast agent, which is very rare. Some common side effects that you may experience is feeling hot. Some uncommon side effects include feeling sick, increased sweating, feeling cold, and dizziness. Allergic reaction symptoms can include mild itching or hives (small bumps on the skin) to shortness of breath and swelling of the throat or other parts of the body. You should tell a staff member immediately if you experience any of these symptoms so you can be treated promptly

Some CT scans are part of standard care and you would have had these anyway, whether you take part in the trial or not. Participation in this trial may mean that you receive 1-3 additional CT scans compared with normal care, depending on your result. These procedures use ionising radiation to form images of your body. Ionising radiation can cause cell damage that may, after many years or decades, turn cancerous. The chances of this happening to you as a consequence of taking part in this trial are approximately 0.3 %. If you are concerned about the level of radiation you are having or have had please talk to your trial team.

Tumour biopsy

You will have a tumour biopsy (removal of a sample of tumour tissue with a special needle) during the screening period. The sample will be around the size of two long grains of rice used to diagnose that you have the type of kidney cancer (clear cell) which may confirm that you are eligible for WIRE. The biopsy will be performed in hospital. A type of imaging (e.g. ultrasound) may be used to help guide the needle and reduce the risk of complications. You will receive a sedative to make you comfortable throughout the procedure. The total duration of the tumour biopsy is 30 minutes, plus an observation period of 6 hours. This period is to manage any side effects appropriately. There may be pain, discomfort and/or bleeding as a result of a biopsy.

This is a research procedure for most participants, performed after your DCE-MRI scan.

Routine Blood and Urine sampling

We will ask you to give blood and urine samples for safety purposes throughout the trial to ensure that you are well enough to receive the trial drugs, and to monitor for any trial drugs

side effects that you may be suffering from. Please see '*Research biosamples*' for a description of the amount of blood and urine that we are taking.

Kidney Surgery (Nephrectomy)
The nephrectomy (surgical removal of a kidney) or partial nephrectomy (removal of the tumour from the kidney) will be performed by your hospital. The kidney surgery is standard of care and should not be affected as a result of taking part in this trial. It is important that you inform the trial team of any side effects as early as possible to ensure they do not affect your surgery should you not suffer serious conditions causing a delay.

Research biosamples
We will ask you to donate extra blood, urine and tissue samples for research related to this trial. These samples will be used to gain more detailed information on how your body and the cancer cells are affected by the trial drugs, and to give further details regarding the biology of the cancer.

The specific details of the research samples taken during this trial are given below.

Research Blood samples
The total amount of extra blood we will take from you for research related to this trial depends on the treatment schedule you are allocated to. We will take a maximum of 31mls of blood at each appointment (roughly 6 teaspoons). Of this 31mls, approximately 13mls will be used for your routine lab tests, and 18mls will be used for research purposes. These blood samples will be taken alongside routine bloods wherever possible. You may experience some discomfort when they are taken. These are timed as follows:

- During the screening period (Routine + Research - 31mls).
- On Day 1 (Routine ONLY - 13mls).
- On Day 15 (Routine + Research - 31mls).
- On the visit that takes place 3 days before surgery (Routine + Research - 31mls).
- At the End of Trial Follow-up visit, 3 months after surgery (Routine + Research - 31mls).

Research Urine samples
We will ask you to give extra urine for research related to this trial, and we may also use these samples to investigate the broader biology of the cancer. You will need to have fasted for these samples- your Research team will advise you regarding the requirements.

Research Tumour Tissue samples
During your nephrectomy, extra small samples of tumour and normal kidney tissue will be taken for research just prior to disconnecting the kidney from its blood supply. This procedure is extra and therefore would not normally be done. It will likely only add around 1 minute to your surgery. Each extra sample we take will be around the size of two long grains of rice put together lengthways, end-to-end. We will aim to take 6 samples. There is an additional risk of bleeding from this biopsy, which is predicted by the Investigators to be very small as your kidney will be removed moments later. This biopsy is important for the research: By looking at this sample (taken from this kidney whilst it still has blood flow), researchers can get the fullest picture of its activity following the drug treatment.

Following the nephrectomy, in the pathology department, the pathologist will take extra tissue from the kidney tumour and normal kidney tissue for research.

10. Are there any additional requirements?

If you agree to take part in this trial, there are a number of things that you must and must not do.

Hospital appointments, tests and assessments

You will need to attend all of the trial hospital appointments arranged by your trial team and agree to the tests needed for this trial.

Other medications

There are certain medications which you cannot take whilst you are receiving the trial drug(s) as the interaction between the medication and the trial drug(s) is not yet known. You must inform your trial doctor of any medications, over the counter medications, supplements, herbal remedies or alternative therapies you are taking or using. Your trial doctor will inform you if any changes are needed. You will also need to check with your trial team before taking any newly prescribed, over the counter medications, supplements, herbal remedies or therapies.

Other clinical trials

Whilst you are receiving the trial drugs you will not be able to participate in any other interventional research studies. It is possible that your participation in this trial may stop you being able to take part in some clinical trials in the future, depending on the nature of that trial.

Dietary restrictions

It is prohibited to consume grapefruit juice or Seville oranges if you are taking olaparib.

Contraception, pregnancy and breastfeeding

Please also share this information with your partner if it is appropriate.

Trial medicines can harm an unborn baby or nursing infant. You will not be able to take part in this trial if you are pregnant or breastfeeding. You should also not participate in this trial if you are planning to become pregnant or father a child during the trial or for six (6) months after the completion of trial treatment.

Women of childbearing potential are required to use two highly effective forms of contraception for the duration of the trial (from signing the informed consent form until end of trial treatment) and for three (3) months after the completion of the trial treatment.

Acceptable non-hormonal birth control methods include:

- Intrauterine Device (IUD) PLUS male condom provided coils are copper-banded.
- True abstinence (where the participant refrains from any form of sexual intercourse and is in accordance with the participant’s preferred and usual lifestyle).
- Vasectomised sexual partner PLUS male condom (with participant assurance that the partner has received post-vasectomy confirmation of azoospermia).
- Tubal occlusion PLUS male condom.

Acceptable hormonal birth control methods include:

- Hormonal shot or injection (e.g. Depo-Provera) PLUS male condom.
- Etonogestrel implants (e.g. Implanon, Norplant) PLUS male condom.
- Intrauterine system (IUS) device (e.g. levonorgestrel releasing IUS – Mirena) PLUS male condom.

Men and male partners are also required to use adequate contraception for the entire duration of the trial and for three (3) months after the completion of the trial treatment. This includes:

- Barrier contraception (condom and spermicide) even if female partner(s) are using another method of contraception or are already pregnant (also to protect male partners from exposure to the trial medicines).
- True abstinence (where this is in accordance with the participant's preferred and usual lifestyle).

Male participants should refrain from donating sperm for the duration of the trial and for three (3) months thereafter.

The risks of the trial drugs to the unborn child are currently unknown, so we would like to follow any pregnancies that occur to yourself or partner, during trial treatment or up to three (3) months after you complete trial treatment, up until the birth. This is so that we can learn more about the safety of the trial treatment.

If you or your partner becomes pregnant during the trial or within three (3) months of stopping treatment, you should inform your trial team immediately. Your trial team will discuss all the options available to you. The outcome and progress of any pregnancy will be followed by your trial team, (with your/your partner's consent), and we will ask you questions about the pregnancy and baby, if appropriate.

For Women of Child Bearing Potential: At the bottom of this document is an optional consent section. This is so that you can consent to the collection of data relating to your pregnancy, should a pregnancy occur.

For Male Participants: Should a pregnancy occur to your partner, your partner will be approached separately with information regarding the treatments you have received, and asked whether they would be willing to give consent for their pregnancy to be followed up.

<u>Private insurance policies</u>
You should discuss your participation in this trial with any insurance provider you have (e.g., protection insurance, life insurance, income protection, critical illness cover and private medical insurance) and seek advice if necessary, as failure to notify them may affect or invalidate your cover.

11. What are the side effects of the drug being tested?

Each person's reaction to a new drug is different. Some people have very few side-effects, while others may experience several. You will be closely monitored throughout the trial, but you should inform the trial team if you feel unwell or different in any way. If you have any major concerns or are feeling very unwell please contact your trial doctor immediately using the contact numbers at the end of this information sheet. Informing the trial team about any side-effects will help them manage your care, and they may be able to prescribe medication

to ease any side effects. We also need to know so that the potential side-effects of this new combination can be recorded. A break from treatment may be necessary if you are experiencing side effects.

Side Effect	***Cediranib***	***Olaparib***
Abdominal Pain		**Very Common**
Bleeding	Common*	
Blood clotting problems	Common	
Blood Pressure Changes*	**Very Common***	
Blood Result Abnormalities	Common	**Very Common**
Cough		**Very Common**
Decreased Appetite	**Very Common**	**Very Common**
Dehydration	Common	
Diarrhoea*	**Very Common***	**Very Common***
Digestive problems	Common	**Very Common**
Dizziness and/or Sickness	**Very Common**	**Very Common**
Headache	**Very Common**	**Very Common**
Hoarse voice	**Very Common**	
Skin problems (itching, rashes)	**Very Common**	Common
Kidney function changes		Common
Mouth complications (dry or sore)	**Very Common**	Common
Pain in fingers & toes	Common	
Shortness of breath		**Very Common**
Thyroid Function Changes	**Very Common**	
Tiredness and/or Weakness*	**Very Common***	**Very Common***
Urinary Complications (pain or bleeding)	Common	
Urinary Tract Infections	Common	
Protein in Urine	**Very Common**	
Weight decrease	**Very Common**	

This table displays the side effects noted by the drug or drugs that you will receive.

Very Common side effects typically affect more than 1 in 10 people

Common side effects typically affect less than 1 in 10 people

Combinations of drugs
Some data has been collected for combinations of drugs that will be taken in this trial. Some side effects were found to be more common when two of the drugs were taken together, than when taken on their own. Such side effects are indicated with an asterisk '*'

Uncommon side effects
In rarer instances (fewer than 1 in 100 people), other symptoms of taking these drugs have been noted that you should keep in mind before you agree to participate in the WIRE trial. Below these rare side effects are listed for the drug that you may be allocated, should you take part;

Cediranib

- Wound healing complications
- Stroke (arterial thromboembolism- cerebrovascular)

Olaparib

- Hypersensitivity reaction
- Itching and redness of the skin with or without a rash (dermatitis/eczema)
- Increase in volume of red blood cells (mean cell volume (MCV) elevation)

Section 2: Conduct of the trial

12. What if new information becomes available?

Sometimes during the course of a trial, new information becomes available which might affect your decision to continue participating in this trial. Your trial team will contact you to discuss the new information and whether you wish to continue participating in the trial. If you still wish to continue on the trial, we will ask you to sign a new Informed Consent Form.

The trial sponsor, the regulatory authority or the trial doctor may decide to stop the trial at any time. If that happens we will tell you why the trial has been stopped and arrange for appropriate care and treatment for you.

13. What if I decide I no longer wish to participate in the trial?

You can stop participating in this trial at any time without giving a reason and without affecting your future care or medical treatment. If you decide not to participate any further, you will no longer receive the trial drugs. No further tests will be performed on you and no further research samples will be collected. Any data or results already collected will continue to be used in the trial analysis. Your trial team may also choose to withdraw you from the trial if they feel it is in your best interests or if you have been unable to comply with the requirements of the trial. Possible reasons for withdrawing you could include:

- Not taking the trial drugs as required
- Experiencing a serious side effect (your doctor will follow-up regarding your progress until this side effect has stabilised or resolved)
- Your disease gets worse whilst receiving trial treatment
- Finding out that you are not eligible after completing the screening tests
- Being unable to complete the visits or trial documentation as required
- Becoming pregnant or planning to become pregnant
- The trial doctor feels you no longer appear to benefit from the trial drugs.

14. What if there is a problem?

Any complaint about the way you have been dealt with during the trial or any possible harm you might suffer would of course be listened to and addressed wherever appropriate. If you have any concerns about any aspect of this trial you should speak to your trial doctor who will do their best to answer your questions.

In the event that something does go wrong and you are harmed by taking part in the research and this is due to someone's negligence then you may have grounds for a legal action for compensation against Cambridge University Hospitals NHS Foundation Trust or the University of Cambridge. The normal National Health Service complaints mechanisms will still be available to you (if appropriate). The University has obtained insurance which provides no-fault compensation, i.e., for non-negligent harm, you may be entitled to make a claim for this.

If you wish to complain or have any concerns about any aspect of the way you have been approached or treated during this trial, you can do this through the NHS complaints procedure. In the first instance it may be helpful to contact the (to be completed locally as appropriate – in England this will refer to the Patient Advice and Liaison Service (PALS)) at your hospital.

15. Will my taking part in this trial be kept confidential?

Cambridge University Hospitals NHS Foundation Trust (CUH) and The University of Cambridge are the Sponsors for this clinical trial based in the United Kingdom. They will be using information from you and your medical records in order to undertake this trial and will act as the data controller for this trial. This means that they are responsible for looking after your information and using it properly. The Sponsor organisations will keep identifiable information about you for 5 years after the trial has finished to ensure your safety and allow the trial to be reviewed by the authorities after it is finished.

Your rights to access, to change or to move your information are limited, as the Sponsor organisations need to manage your information in specific ways in order for the research to be reliable and accurate. To safeguard your rights, we will use the minimum personally-identifiable information possible.

You can find out more about how the Sponsor (s) use(s) your information using the information below:

- For Cambridge University Hospitals NHS Foundation Trust, please visit: https://www.cuh.nhs.uk/corporate-information/about-us/our-responsibilities/looking-after-your-information

Or email the Data Protection Officer at: gdpr.enquiries@addenbrookes.nhs.uk

- For University of Cambridge, please visit:
https://www.medschl.cam.ac.uk/research/information-governance
Or email the Information Governance team at: researchgovernance@medschl.cam.ac.uk

For participants recruited at CUH (where the Sponsor is also the site):

Cambridge University Hospitals will collect your name, NHS number and contact details to contact you about this trial and make sure that relevant information about the trial is recorded for your care and to oversee the quality of the trial. Individuals from the Sponsors and regulatory organisations may look at your medical and research records to check the accuracy of this trial. Cambridge University Hospitals will pass these details to the Sponsors along with the information collected from you and/or your medical records. The only people in the Sponsor organisations who will have access to information that identifies you will be people who need to contact you in relation to this trial and to audit the data collection process. Cambridge University Hospitals will keep identifiable information about you from this trial for *5* years after the trial has finished.

For participants recruited at other participating sites:

(Add site name) will keep your name, NHS number and contact details to contact you about this trial, and make sure that relevant information about the trial is recorded for your care, and to oversee the quality of the trial. Certain individuals from the Sponsors, and regulatory organisations may look at your medical and research records to check the accuracy of this trial. The Sponsor(s) will only receive information without any identifying information. **(Add site name)** will keep identifiable information about you from this trial for *XX* years after the trial has finished.

All information collected about you as a result of your participation in the trial will be kept strictly confidential. Your personal and medical information will be kept in a secured file and be treated in the strictest confidence.

Once you have agreed to participate in this trial you will be allocated a Trial ID Number. This is a unique trial number which will be used on all your trial documentation along with your date of birth. Your date of birth is considered to be personal information. We collect this personal information on trial documentation to help ensure that the data we receive as part of your trial participation is correctly allocated to you. By cross checking these two unique references we can ensure the integrity of the data.

When you agree to take part in this trial, the information about your health and care may be provided to researchers running other research studies in this organisation and in other organisations. These organisations may be universities, NHS organisations or companies involved in health and care research. Any serious side effects that occur during the trial will also be shared to the manufacturer of the trial drugs (AstraZeneca) using your trial-specific identifiers. The statistics for this trial is being undertaken by the University of Newcastle. All data that is shared with the University of Newcastle will be identified using only your trial ID and date of birth.

Your information will only be used for the purpose of health and care research, and cannot be used to contact you or to affect your care. It will not be used to make decisions about future services available to you, such as insurance.

Your coded trial data may be sent to other country(ies) outside the European Economic Area (EEA) for analyses, where the data protection laws are not the same. Your coded personal data may also be shared with off-site licenced laboratories for the purpose of analysing samples collected from you in this trial. However this information will not identify you and will not be combined with other information in a way that could identify you. The people who analyse the information will not be able to identify you and will not be able to find out your name, NHS number or contact details. Only anonymous trial data, without any personal information will be published at the end of the trial.
We will need to inform your GP of your participation in this trial. This is so that any medical decisions that your GP makes will take into account the fact that you are receiving drugs as part of this trial.

16. What will happen to my samples and scan images?

With your permission, we will analyse the blood, urine, kidney and tumour samples collected from you during this trial. This analysis will take place over a long period of time, so any results obtained will not alter your care pathway.

If you decide to stop taking part in the trial please tell the trial doctor if you want to change your mind about us using your donated sample(s) to look at the molecular and genetic makeup of the cancer.

<u>Routine blood samples</u>
Blood samples taken will be analysed immediately by your hospital and will be destroyed once the laboratory tests are complete. The results will include those you would receive as part of your standard care pathway, plus any results required for the trial. These results will be accessible to your trial team.

<u>Research samples</u>
Research samples taken will be labelled with your Trial ID Number, processed and stored initially by your trial team at your hospital, before being transferred to an approved facility for analyses. The approved facilities are either part of the Cambridge Biomedical Campus or

AstraZeneca. These labs may be inside or outside of the UK. The samples will be securely stored and analysed by specialist teams. You will not be identifiable to researchers from any of the laboratories we may use for research analysis.

These samples will be analysed in a way which will tell us whether WIRE trial treatments are eliciting the biological response that we hope and expect to see. There may be other tests that we perform on your research samples to give us additional information regarding the drug activity.

Once the research analysis is complete any remaining samples will either be:

- Stored by the research team in line with the relevant regulatory requirements, in an appropriate facility pending ethical approval for use in a future trial outside of the WIRE trial.
- Will be disposed of in accordance with the UK Human Tissue Authority code of practice.

<u>Archival tumour</u>

You may have previously had a biopsy of your kidney, and therefore have more than one tumour sample. Not all patients will have undergone a previous biopsy. If you have however, we will ask your permission to access this 'Archival' tissue from your local hospital, so that we can compare the changes with your recent biopsy sample. This will also help us in the event that your most recent sample isn't big enough for us to perform our analysis. Granting us permission to access these samples is entirely optional.

<u>Genetic Testing (All samples)</u>

All of our cells in our body get information on how to work and function through a molecule called deoxyribonucleic acid (DNA). DNA is a record of instructions where the instructions are called genes. Genetic testing looks at your genes and could either look at one or some specific genes or all of your genes – your whole DNA. Genes affect how we grow and develop. Nobody else has exactly the same genes as you do, unless you have an identical twin. These differences mean that some people are more likely than others to get certain diseases and they also mean that medicines affect people differently. Some genes can be important in more than one disease.

Genetic testing in this trial is only for research purposes, but is an essential part of the trial. If you do not want to agree it to, you will be unable to take part. These genetic tests will be performed on any samples you donate to the trial, and requires no additional time from you.

The trial team may also undertake laboratory tests using your samples that reenact the environment of natural body conditions in a laboratory setting - so called '*ex vivo* modelling'.

This method of analysis typically provides very meaningful data regarding how cells interact naturally with the drugs received. Tissue cells are grown in a laboratory environment and could be modified to implant in mice with low resistance to disease. The laboratory staff will monitor the growth of the tumour (derived from your own) within the mice. Any laboratory performing this analysis would have the appropriate license for testing on mice. You have the right to take part in the trial without these particular lab procedures taking place. If you give your optional consent for these procedures, you may withdraw your consent for your samples to be used in this way at any point until this laboratory analysis begins.

Cambridge University Hospitals NHS Foundation Trust & the University of Cambridge may do this testing in its own laboratories or it may be done in the laboratories of other organisations (such as universities, collaborators and genetic specialist), or both. Results

from this research may be shared and used as described in 'Will my taking part in this trial remain confidential?' In addition Cambridge University Hospitals NHS Foundation Trust & the University of Cambridge, and its designated organisations may conduct future research where they will share summary results (not your individual results) or anonymous datasets with other researchers, from other companies or universities, for example. The results may be combined with the results of other studies, for example as part of scientific databases. The researchers can only use this information for medical and healthcare related research.

Radiological (Scan) Images
The images obtained by way of CT and/or MRI scans will be coded, using only your trial ID number and Date of Birth. These coded images will then be sent on a secure CD, through the post, to the Sponsors (Cambridge University Hospitals and the University of Cambridge). These images will be stored on password-protected computers at Cambridge University for the duration of the trial.
CT scans will be used by your doctor to monitor general size changes to the tumour, and will likely be kept at your local hospital, but the central trial team may request them for checking of the measurements provided by your hospital. The people performing the analysis on your MRI images will not be able to identify you. They will be experienced Radiologists that will be able to identify areas of the cancer to get a general idea of how the cancer is behaving; so called 'Regions of Interest'. By using specialist software, the Radiologists can measure the amount of blood flowing through the cancer. This analysis will be part of the assessment of your MRI scan performed before your treatment, and after your treatment for comparison.

The anonymised images may support future research projects, for example finding new ways of gaining even more insight into renal cancer. Therefore, we ask for your permission to use these images, which do not identify you personally, in other future research projects.

17. What will happen to the results of the trial?

The results of the trial will be anonymous and you will not be able to be identified from any of the data produced. When the results of this trial are available they may be published in peer reviewed medical journals and used for medical presentations and conferences. They will also be published on the EU Clinical Trials Register website, a central registry for all clinical trials conducted in the EU.

Anonymised datasets from the trial will be made available to the funder (AstraZeneca), and may also be made available to other researchers in line with national and international data transparency initiatives.

If you would like to obtain a copy of the published results please contact your trial doctor directly who will be able to arrange this for you.

18. Who is funding the trial?

The trial is being funded by AstraZeneca and the Cancer Research UK Cambridge Centre. AstraZeneca is also supporting this trial by providing the trial drugs free of charge.

The trial is being managed by the Cambridge Clinical Trials Unit – Cancer Theme. The Chief Investigator is Mr Grant Stewart who is a University Lecturer at the University of Cambridge and Honorary Consultant Urological Surgeon at CUH.

19. Who has reviewed this trial?

All research within the NHS is reviewed by an independent group of people called a Research Ethics Committee, to protect your interests. This trial has been reviewed and given

favourable opinion by the (name of REC here). The Medicines and Healthcare Products Regulatory Agency (MHRA) who are responsible for regulating medicines in the UK have also reviewed this trial.

20. Further information and contact details
If you have any questions concerning this trial please contact your:

Trial Doctor
Name: [INSERT your hospital contact details here]
Tel: [INSERT your hospital contact details here]
Research Nurse
Name: [INSERT your hospital contact details here]
Tel: [INSERT your hospital contact details here]

In the event of an emergency please contact:

24-hour contact details
Contact: [INSERT your hospital contact details here]
Tel: [INSERT your hospital contact details here]

Outside of an emergency,
You may wish to contact one of the following organisations that are independent of the hospital at which you are being treated:

CancerHelp UK is a registered charity providing information about all aspects of cancer. It can provide useful information on cancer treatments and medical research. You can contact their nurses on freephone 0808 800 4040. You can also access their web site at: www.cancerresearchuk.org

MACMILLAN Cancer Support is a registered charity providing information about all aspects of cancer. They have published several useful booklets on different types of cancer, chemotherapy, radiotherapy, and clinical trials in general. You can contact the nurses on freephone 0808 808 0000. You can also access their website at www.macmillan.org.uk

If you wish to complain or have any concerns about any aspect of the way you have been approached or treated during this trial, you can do this through the NHS complaints procedure. In the first instance it may be helpful to contact the Patient Advice and Liaison Service (PALS) at your hospital.

Patient Advice and Liaison Service (PALS) at:
Hospital Name: [INSERT your hospital PALS contact details here]
Tel: [INSERT your hospital PALS contact details here]

TO BE PRINTED ON HEADED PAPER

UNIVERSITY OF CAMBRIDGE   Cambridge University Hospitals NHS Foundation Trust

INFORMED CONSENT FORM

Trial Title: WIRE: WIndow-of-opportunity clinical trials platform for evaluation of novel treatments strategies in **RE**nal cell cancer.

Principal Investigator: [INSERT your PI name here]

Participant Number: __________ (please add once the participant is registered)

**If you agree with each sentence below, please initial the box**		**INITIALS**
1	I have read and understood the Group 2, Cediranib & Olaparib Combination, Participant Information Sheet version 3.0, dated 10th February 2020 for the above trial and I confirm that the trial procedures and information have been explained to me. I have had the opportunity to ask questions and I am satisfied with the answers and explanations provided.	
2	I understand that my participation in this trial is voluntary and that I am free to withdraw at any time, without giving a reason and without my medical care or legal rights being affected. I also understand that data or samples already collected will continue to be used in the trial analysis.	
3	I understand that personal information about me will be collected and used in accordance with this information sheet. This information will be kept in the strictest confidence and none of my personal data will be published.	
4	I understand that sections of my medical notes or information related directly to my participation in this trial may be looked at by responsible individuals from the sponsor, regulatory authorities, research personnel where it is relevant to my taking part in research and that they will keep my personal information confidential. I give permission for these individuals to have access to my records.	
5	I understand that my GP will be informed of my participation in this trial and sent details of the WIRE trial.	
6	I have read and understood the compensation arrangements for this trial as specified in the Participant Information Sheet.	
7	I understand that the team/doctors in charge of this trial may close the trial or stop my participation in it at any time without my consent.	
8	I have read and understood my responsibilities for the trial including using appropriate contraception as listed in section 6.	
9	I agree to give blood and urine for research related to this trial.	
10	I agree to give tumour and healthy tissue samples for research related to this trial	
11	I give my permission to allow my kidney tissue and research blood, urine & tumour samples to be sent to specialist teams on the Cambridge Biomedical Campus, or to laboratories contracted and approved by the Sponsor(s) for tests/analysis related to this trial. I understand that only the minimum information will be supplied and that I will not be identifiable to any of the researchers.	
12	I understand that the results from the analysis carried out on the research blood and tumour samples will not be fed back to me.	
13	I understand that the information collected about me will be used to support other research in the future, and may be shared anonymously with other academic and commercial researchers external to the	

TO BE PRINTED ON HEADED PAPER

UNIVERSITY OF CAMBRIDGE   Cambridge University Hospitals NHS Foundation Trust

	project, within the UK and beyond		
14	I give my permission to allow for DNA testing of my samples		
	**OPTIONAL:** Please read each statement below and initial the relevant box	**INITIALS**	
		Yes	No
15	I give my permission to allow for any archival tumour tissue to be collected for tests related to this trial. I understand that only the minimum information will be supplied and that I will not be identifiable to any of the researchers.		
16	I give my consent for my tissue samples to be used to grow cell cultures or be implanted into mice		
17	I give my permission to allow any remaining research blood and tumour samples taken as part of this trial to be retained in an HTA licenced facility pending Ethical Approval for use in another project at the discretion of the Chief Investigator. I understand that these samples will be retained in an approved storage facility, that only the minimum information will be supplied and, that I will not be identifiable.		
18	I give my permission for my anonymised radiological images to be used as part of the data for future research studies		
	**FOR WOMEN OF CHILDBEARING POTENTIAL ONLY**<br>**OPTIONAL:** Please read each statement below and initial the relevant box	**INITIALS**	
		Yes	No
19	If I become pregnant during, or in the three (3) months after receiving the trial drugs, I agree to information being collected about me, my pregnancy and my baby.		
20	I understand that sections of my medical notes or information related directly to my pregnancy may be looked at by responsible individuals from the sponsor, regulatory authorities and research personnel where it is relevant to my taking part in research. I give permission for these individuals to have access to my records.		
21	I agree to give my pregnancy information voluntarily and understand that I am free to withdraw at any time without giving a reason and without my medical care or legal rights being affected. I understand that all data collected up to the withdrawal of consent will be kept confidential.		

I agree to participate in this trial:

______________________ ______________________ ______________________

Name of participant **Signature** **Date**

______________________ ______________________ ______________________

Name of person taking consent **Signature** **Date**

Time of consent (24hr clock): _______:_______

1 copy for the participant, 1 copy for the trial team, 1 copy to be retained for hospital records

TO BE PRINTED ON HEADED PAPER

UNIVERSITY OF CAMBRIDGE

Cambridge University Hospitals NHS Foundation Trust

WIRE

NOVEL TREATMENTS IN RENAL CELL CANCER

Group 3 - Olaparib Only

PARTICIPANT INFORMATION SHEET & INFORMED CONSENT FORM

WIRE: WIndow-of-opportunity clinical trials platform for evaluation of novel treatment strategies in **RE**nal cell cancer.

You are being invited to take part in a research trial. Before deciding whether to take part, you need to understand why this research is being done and what it involves.

Please take time to read the following information carefully and talk to others about the trial if you wish. Please ask your trial team if anything is not clear or if you would like more information. Please take time to decide whether or not you wish to take part. You will be receiving this Participant Information Sheet before any diagnosis has been made i.e. it is not yet confirmed that you have renal cell cancer.

Section 1 tells you the purpose of this trial and how the trial would involve you, including a description of what the various tests are, and some of the risks and restrictions of taking part

Section 2 gives you more detailed information about the trial process, including information about how the trial is run, and how we may use your data.

Section 1: Purpose of the trial and what will happen

1. What is the purpose of the trial?

Kidney cancer is the 7th most common cancer in the UK. Currently many patients with this cancer are offered a kidney removal operation (known as nephrectomy), which cures many patients with no further need for treatment. However, some may receive drug treatment after surgery.

WIRE is a trial that will administer drugs to patients in the time between the decision to operate and the nephrectomy operation. Patients are not usually given anti-cancer drugs during this period, and indeed may never need them, but it could give researchers important information to guide the best combinations of anti-cancer drugs to prioritise for later phase clinical trials. The trial will investigate the effect and safety of 3 different cancer drugs, individually or in combination, in patients with a specific type kidney cancer called clear cell renal cell cancer. The cancer may be localised (within the kidney) or metastatic (it has spread outside of the kidney).

2. **What are the drugs being tested?**

There are 3 drugs being used in this trial, Cediranib, Olaparib and Durvalumab. All three of these drugs target different parts of cancerous cells which might decrease the size of the tumour over a period of time. However, the duration of treatment in this trial is very short in comparison to how long these drugs would normally be given for. Therefore, it is unlikely that the trial treatment will noticeably reduce the tumour size if it is confirmed that you do have kidney cancer. . In this trial, we will primarily be looking at cancer's response to these drugs by taking a closer look at the tumour cells in the laboratory and by using new imaging technology.

Although the trial uses 3 drugs, we will only be giving you one or two of these drugs. The drugs you are given will depend on the group you are allocated. The trial team already know which group you will be allocated. Currently, we are recruiting participants for the **Olaparib** group. Each group has its own specific entry criteria which may involve different tests and assessments; this is discussed further under *Section 4 Do I have to take part?*

Olaparib is also a cancer therapy drug called a 'PARP Inhibitor'. PARP is a protein which helps damaged cells to repair themselves. "PARP Inhibitors" block the ability of damaged (cancer) cells to repair themselves. Olaparib is being tested in clinical trials, but is currently only licenced in the EU for use in patients with ovarian and breast cancers. This drug is an oral tablet (a tablet taken by mouth), which will be taken for at least 14 days in total, and until the morning of your surgery.

3. **Why have I been invited?**

You have been invited to participate in this trial because you may have renal cell cancer (kidney cancer) that may not be cured with surgery alone. The diagnosis of renal cell cancer will be confirmed or disproved with a pre-surgery biopsy. In normal care, usually your treating doctor will recommend proceeding with a nephrectomy or partial nephrectomy (removal or partial removal of the kidney) without the need of a pre-surgery biopsy. However, as part of the WIRE trial, participants will be required to undergo a biopsy during the screening stage to determine whether they have the type of cancer we are investigating.
We plan to include up to 76 patients with kidney cancer from approximately 2 hospitals across the UK.

4. **Do I have to take part?**

Participating in this trial is completely voluntary. If you decide to participate you will be asked to sign an Informed Consent Form. You are however still free to change your mind and leave the trial at any time and without giving a reason. If you chose not to participate or to leave the trial, your current and future medical treatment will not be affected in any way. Your normal standard care will not be affected in any way.

You should also be aware that entry into this trial requires you to meet some strict entry criteria that your doctor will assess you for over the next few weeks, during a period called 'screening'. At the moment we will not know whether you are eligible for the trial as there are further tests and assessments to do, but some people who consent to take part in this trial may not meet the entry criteria to receive the trial drugs.

Before proceeding, it is important to understand that you may not benefit from the extra drugs and procedures involved in this trial. Surgery alone may be sufficient to cure the cancer alone, although there is no way to predict this for certain. The possible side effects could potentially delay your surgery, and participation in the trial could make you feel

fatigued and nauseous. However, your doctors will be monitoring how you are feeling very closely, making every attempt to minimise any side effects.

It is also important you are aware that based on previous research the average chance of being cured by surgery alone needing no further treatment is 42%, in whom no further treatments would be needed. It is also important that you are aware that you will not have access to the trial drugs after your operation, and you might not be permitted to take part in other clinical trials if you decide to participate in the WIRE trial.

5. Expenses and payment

You will not receive any payment for participating in this trial however we can reimburse reasonable travel and parking costs incurred by your participation in this trial. This is up to a maximum of £180 for the entire duration of your participation in this trial. We will ask you to retain your receipts wherever possible.

6. What are the possible advantages and disadvantages of taking part?

It is unlikely that you will benefit clinically from taking part in this trial, although it is possible that the tumour may shrink very slightly. It is also hoped that taking the medications used in the trial could provide you with a better outcome, but this cannot be directly measured in this trial. You will gain access to drugs that are only available within a clinical trial. You are likely to have more contact and for longer with your care team than if you were not involved in a trial, and some patients gain reassurance from this. It is hoped that information collected as part of your participation in this trial will benefit patients with kidney cancer in the future and you may contribute to their future treatment and care. Your care team will be able to share information with you regarding how the tumour cells responded and/or any tumour size reduction, if you wish to know this.

In addition to the above benefits there are also aspects of your participation you may find difficult. For instance, if you decide to take part:

- You will need to undergo a biopsy at the screening stage which is likely to require a full day at the hospital. It is also likely that this biopsy would not have been required if you were not participating in this trial. This is an extra procedure for about 70% of patients. You can find more information about this under section 9 – Tumour biopsy on page 12.
- There will be an increased number of hospital visits due to participating in the trial. You will be required to come to the hospital an extra 4-5 times during the trial. These visits are to conduct initial assessments and scans (1 extra visit); clinic visits whilst receiving the trial drug(s) (2-3 extra visits); a visit for the scan before surgery (1 extra visit). The average length of time per visit is about 2 or 3 hours, but actual time may vary. The schedule and timing of these visits is available on page 9.
- You will undergo at least an additional 2 MRI scans if you decide to take part in this trial, with potentially another 2 MRI scans using a new type of scan called a ‘hyperpolarised MRI’ if available at your hospital (see section 9, pages 11 and 12 for more information). An additional 1-2 CT scans may be required as recommended by the doctor based on information from your diagnosis and previous scans. You would not have normally received these scans if you were not participating on the trial.

- You could experience drug side effects such as nausea, fatigue, diarrhoea, dizziness, decreased appetite and blood test abnormalities. A full list of known side effects is listed on page 15 and 16. It is also possible that some of these side effects might delay your surgery.
- There will be 6 additional tissue biopsies (samples) taken during your surgery, this will likely only add a few minutes to your standard surgical procedure. Whilst these are unlikely to impact you or prolong your stay in hospital, it is important that you are aware these are taken. More information about these tissue samples is mentioned on page 13.

  The drugs you will be taking as part of this trial will not be given to you after the surgery, and may prevent you from taking part in other clinical trials after your surgery.

7. What are the alternatives for treatment?

The standard care for you is a nephrectomy (removal of the kidney) or partial nephrectomy (removal of the tumour only) without any drug treatment before surgery.

8. What happens when the trial stops?

Following completion of the trial you will not be able to receive any further trial drug treatment and we will refer you back to your primary consultant. You will still be able to contact your trial Nurse or Practitioner, however, with any questions about the trial.

9. What will happen to me if I take part?

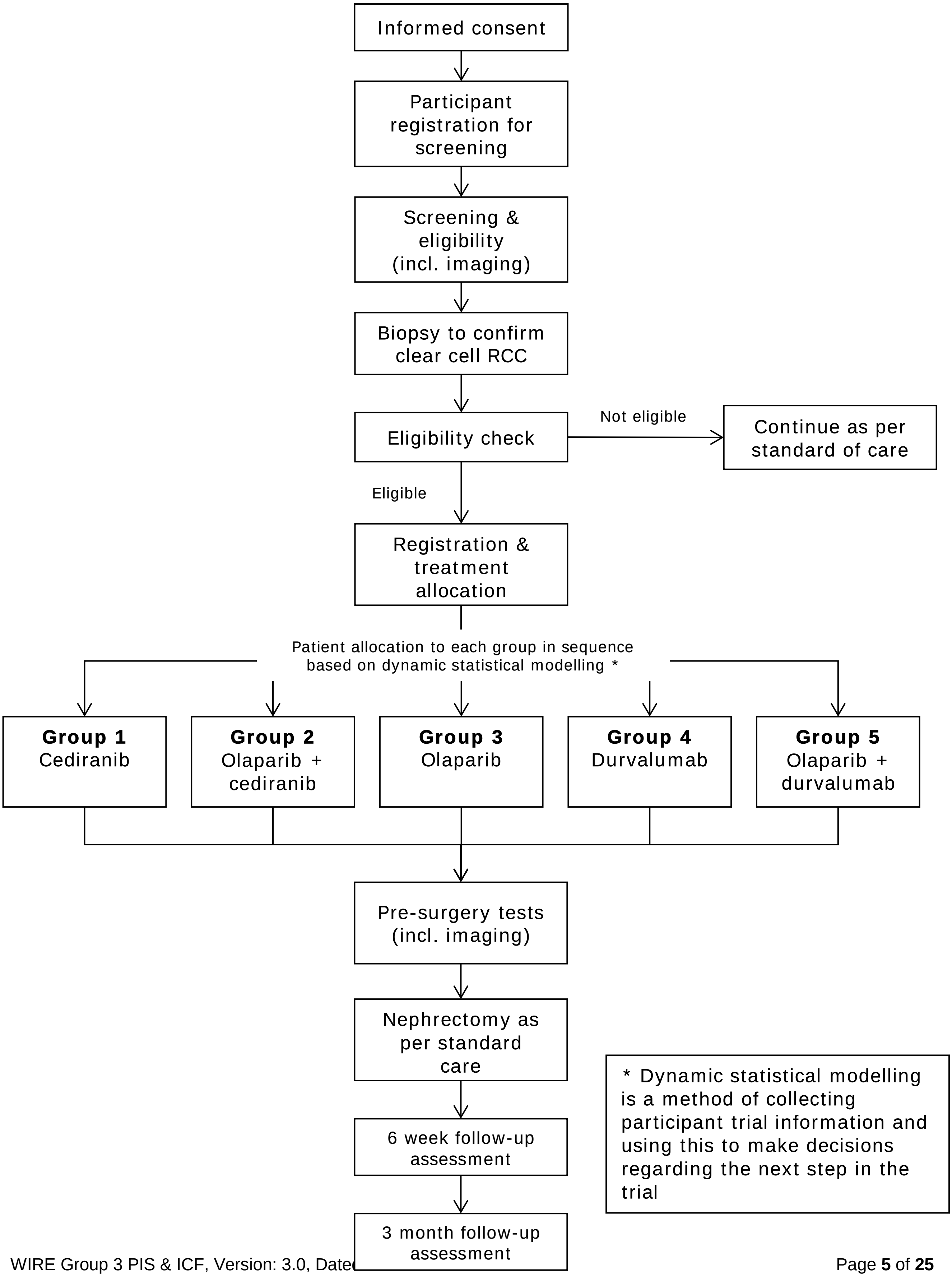

Hospital Appointments

Informed Consent
If you agree to participate in the trial, you will be invited to sign the Informed Consent Form at the end of this document. You will be given a copy of this to take away and refer to later.

Screening and baseline
Standard of care visit plus extra trial assessments 5-6 hours in duration. Screening may require an additional visit to complete all assessments, dependent on hospital capacity.
To check that you meet all of the requirements for entering this trial you will need to have some tests. This is called the “screening period”. You will be given appointments to see the trial team for these tests, which will be carried out within a 28 day period. This may include more than one visit to the hospital. All of the tests listed during this period and the following visits are further explained later in this information sheet (see page 5). All of the tests listed under Screening will collect data for the research trial.

- A check of your weight, blood pressure and temperature
- A full physical examination
- Clinical review
- Performance status check
- Blood and urine sampling
- 3 Electrocardiogram assessments (ECGs), 5 minutes apart.
- A minimum of one type of scan, including a Dynamic Contrast Enhanced Magnetic Resonance Imaging (DCE-MRI) scan at least (a type of scan that uses strong magnetic fields and radio waves to produce detailed images of the body and organs). You may undergo a hyperpolarised MRI scan (a new MRI scanning technique which produces extremely detailed images of the body and organs), depending on the group you are allocated to, and the treatment centre you are attending. You may also undergo a CT scan, depending on how long ago your last CT scan took place (the risks of increased radiation exposure are explained further on in this document, in the ‘Tests and Assessments’ section).
- Collection of your Medical History
- Research blood and urine sampling- we will take these samples before you have the tumour biopsy, on the same day
- Tumour Biopsy
- Pregnancy test (female participants only)

Your weight, blood pressure, temperature, physical examination, performance status check, blood & urine samples, CT and ECGs may be assessed as part of your standard care pathway (these would usually take place without trial participation anyway as part of the usual pre-surgery checks). A tumour biopsy would not normally take place in about 70% of patients , but all participants will have a biopsy in order to be eligible for the trial.

Additional ECGs, a possible repeat CT scan (if you haven’t had one within the last 28 days) and all of the other assessments and scans listed are extra for the trial.

Registration
Some of the tests performed during Screening require the results to fall within certain trial ranges. If any of your results do not fall within these ranges, you will not be able to participate in this trial and will be referred to follow the usual treatment pathway for patients with your condition. However, if the screening tests show that you are suitable to participate in this trial, your clinical team will register you into the trial.

There are 5 different treatment groups in this trial, but each participant will only be allocated to one of these groups, and given the appropriate information sheet for the drug that they might receive. If you decide to participate and meet the entry criteria, you will be allocated to Group 3 and be given Olaparib.

Clinic Visits

Extra visits for the trial, up to 3 hours per visit in duration

Once your participation in this trial has been confirmed we will ask you to attend regular clinic appointments to see the trial team whilst you are receiving the trial drug/s and we will perform a number of trial related tests to ensure you are tolerating the trial drugs well. Initially these will be performed on the 1st and approximately 15th day of your treatment but will be flexible around weekends and bank holidays. You will need to have fasted for 6 hours before the Research urine tests, and your research/care team will advise you regarding the timescales on this. Both of these visits are extra visits for the trial and all assessments for these visits below are needed just for the trial and are outside of standard care:

- A weight, blood pressure and temperature check
- A physical examination (if your doctor believes it is necessary)
- Performance status check
- Clinical review
- At least 1 electrocardiogram assessment (ECG). Based on your results, your trial team may ask you to perform 2 additional ECGs, all 3 taken 5 minutes apart.
- Blood and urine assessments. Pre-treatment (Day 1)
- Pregnancy test (female participants only)
- Dispensing of your tablets to take home, and a diary to record when you take them (at Day 1 only).

In addition to the assessments above, the following tests will be performed on Day 15 ONLY

- Research blood and urine sampling
- Assessment of any side effects from the medication
- Check that you have enough and are correctly taking, your medication

Your clinical trials team may decide to give you an additional assessment on approximately the 22nd day of treatment, if you are still taking the trial drug. This may be by telephone or in clinic; this day 22 assessment will be an extra assessment for the trial, and the below tests will be extra assessments for the trial, outside of standard of care:

- Performance status check
- Assessment of any side effects from the medication
- Check that you have enough and are correctly taking, your medication
- Clinical review

If you experience unmanageable side effects, or if your trial doctor thinks you should not continue, the trial drugs will be stopped and you will return to the normal standard of care provided by the hospital for the treatment of the cancer.

Pre-surgery

Extra visit, up to 6 hours in duration

Within 3 days of your surgery date, you will undergo at least one scan to assess whether the medication you have been taking is affecting the tumour. These are all extra assessments for the trial, outside of standard care, and the visit could take most of a day or more than one day:

- DCE-MRI

- Hyperpolarised MRI (depending on the group you are allocated to, and the treatment centre you are attending)
- CT scan (if the cancer has spread outside the kidney- your Trial Nurse or Practitioner will advise you)
- Research blood and urine sampling (these are outside of standard care and are required for the trial only)
- Routine blood sampling

Surgery

Standard of care visit. Surgical prep plus 3 hours surgery time.

During your nephrectomy, we will obtain tumour samples, as well as samples of the normal kidney tissue next to the tumour. The surgery is standard care but the amount of samples taken will be higher than in standard care.

Follow-up – 6 weeks after surgery

Standard of care visit, up to 2 hours in duration

You will be given an appointment to see the trial team approximately 6 weeks after surgery to ensure that you have recovered from any side effects. This 6 week check is usual for patients who have undergone nephrectomy, and is standard care. During this visit you will have:

- Performance status check
- Clinical review
- Routine Blood sampling
- Pregnancy Test

End of trial follow-up – 3 months after surgery

Standard of care visit, up to 2 hours in duration

You will be given another appointment to see the trial team approximately 3 months after surgery to ensure that you have tolerated the trial drugs well. A CT scan will be performed prior to this visit. Following this visit, you will not need to attend any further trial related visits. This 3 month check is usual for patients who have undergone nephrectomy, and is standard care. During this visit you will have:

- Performance status check
- Clinical review
- Routine blood sampling
- Research blood and urine sampling (these are outside of standard care and are required for the trial only)
- Pregnancy test

TO BE PRINTED ON HEADED PAPER

UNIVERSITY OF CAMBRIDGE

Cambridge University Hospitals NHS
NHS Foundation Trust

<u>What will happen and when?</u>

Tests and Assessments	Screening (up to 28 days prior to Day 1 of treatment)	Treatment phase			Pre-surgery	Surgery	Follow-up	
		**Day** 1 (± 5 days)	**Day 15** (± 5 days)	**Day 22 (Optional)** ± 5 days			6 weeks after surgery ± 7 days	3 months after surgery ± 14 days
Consent	X							
Medical history	X							
Vital signs	X	X	X					
Performance status check	X	X	X	X			X	X
Clinical review	X	X	X	X			X	X
Physical examination	X	If clinically necessary	If clinically necessary					
ECG	X	X	X					
Urine sample	X	X	X				X	X
Blood for routine tests	X	X	X		X		X	X
Pregnancy test	X	X	X					X
Post-menopausal test	X							
Blood for research tests	X		X		X			X
Urine for research tests	X		X		X			X
DCE-MRI	X				X			
Hyperpolarised MRI	If needed				If needed			
CT scan (*your doctor will tell you if needed)	X*				If needed*			X
Tumour biopsy (screening)	X							
Olaparib Drug dispensing		X						
Olaparib Drug compliance check			X	X				
Tissue samples (research)						X		
Full or partial nephrectomy						X		
Total time of visit	5-6 hours over 2-3 visits	2-3 hours over 1 visit	2-3 hours over 1 visit	1-2 hours over 1 visit or 20 min phone call	4-6 hours over 1-2 visits	2-3 hours of surgery	1-2 hours over 1 visit	1-2 hours over 1 visit

Tests & Assessments

You will be monitored carefully by the trial team whilst you are on this trial. Most of the tests in this trial are necessary to give you the trial drugs safely and ensure you are tolerating the trial drugs well. These tests are explained below, including the possible **risks and/or benefits** of each.

Medical history
Your medical history will be reviewed by the trial team during the screening period. This will involve a discussion about your past and present health, lifestyle and any symptoms you may currently have. This is so that the trial team are aware of any pre-existing conditions. The trial team will also record your gender, ethnicity and date of birth. This is to collect demographic data for the trial.

Weight, blood pressure and temperature
The trial team will monitor your blood pressure and temperature in order to properly assess the side effects to your treatment. These indicators will help the doctors to properly diagnose you and to manage your care.

Clinical review
The trial team will regularly discuss and review your general wellbeing, discuss any symptoms and/or side-effects that you may have, and record any medications you may be taking. This is so that the trial team has a full picture regarding your current state of health.

Your trial team may end your participation in this trial if you have severe or unmanageable side-effects to the trial drug/s. If this happens your trial team will follow-up with you regarding your progress until the side effect has stabilised or resolved.

Physical examination
Your trial doctor will perform a physical examination during the screening period and whilst you are receiving the trial drugs. This will ensure that the trial team has a full picture regarding your current state of health.

Drug Compliance Check
It is important that you take the trial drugs as directed by your trial team. We will provide you with a diary to log the medication that you are taking as part of this trial (olaparib). You must also return any tablets or the empty container to the trial team at the end of your treatment and bring your diary with you to each visit.

Electrocardiogram (ECG)
You will be asked to have several ECGs as part of this trial during the screening period and whilst you are receiving the trial drugs. These record an electrical trace of your heart rhythm and are required to confirm that you are fit enough to receive the trial drugs. They will also monitor the health of your heart during the trial. We will ask you to lie down whilst a series of leads are placed on your chest area, wrists and ankles with adhesive pads, these will record a trace of your heart rhythm. This takes a few minutes and is painless. This is to check the health of your heart.

Performance Status check
Your performance status check will track the impact that your condition or treatment has on your day-to-day activities and on your “usual” level of day-to-day functioning. This is

assessed by asking you questions about your level of activity around the house or at work (as applicable).

Pregnancy test/ Non-childbearing status (female participants only)

If you are female and of childbearing potential you will be asked to give a blood sample during the screening period **to confirm that you are not pregnant before you can be enrolled into this trial.** You will also have several pregnancy tests during the trial. If you are of non-childbearing potential you may be asked to provide a sample of blood during the screening period to confirm your non-childbearing status. This is because we do not yet know the effect of the trial drugs on a developing baby (foetus). This is an extra sample but can be taken from the same bottle as the other blood tests. Most female patients would normally have a urine test.

MRI Scans

You will undergo two DCE-MRI scans if you participate in this trial, and may also have two hyperpolarised MRI scans. It is important that you are aware that the MRI scans would not have been required if you were not participating on this trial. You may need an injection of a contrast agent to improve the quality of the scan. The injection is usually given in a vein in your elbow (the same place as a blood test), which may cause discomfort and/or bruising. When you have the scan, you will be asked to lie still on a couch which will slide into a metal tunnel, which some people find this uncomfortable and/or claustrophobic. The scan lasts for about 45-60 minutes. You will also be able to talk to the radiographer whilst you are having your scan, or listen to music if you prefer. If you are anxious in enclosed spaces you may be prescribed a tablet to help you relax before you have the scan.

DCE-MRI scan

We will ask you to have a DCE-MRI scan during the screening period and before your surgery. DCE-MRI is a type of scan that uses strong magnetic fields and radio waves to produce detailed images of the body and organs. We do this to establish how the trial drugs affect blood flow in the tumour and how the cancer is responding to the trial drugs.

It is known that small amounts of these contrast agents may remain in the brain after a scan with these agents, although there is currently no evidence that these small amounts cause any harm and these agents have been used for many decades. These contrast agents are routinely given during a kidney cancer MRI so we can have a clear understanding of the blood supply to the cancer. You may experience some side effects such as nausea, headaches and dizziness which are very common side effects. Other side effects such as vomiting, injection site reaction, laboured breathing, and allergic reactions are less common. You should tell a staff member immediately if you experience any of these symptoms so you can be treated promptly. We will use the lowest dose of the contrast agent required for a clear image and if you have any questions about your scan, please speak to your doctor.

Hyperpolarised MRI

You may also be asked to have hyperpolarised MRI scans during the screening period and before your surgery. These scans will enable researchers to assess the cancer tumours and the effectiveness of trial drugs. Not every participant will receive this scan, depending on the numbers already in the trial and local availability.

The contrast agent to be injected is a breakdown product of sugar called pyruvate. Although hyperpolarized carbon MRI is a new technology, tests to date have demonstrated no significant safety issues. Pyruvate is a naturally-occurring molecule in the body. Rarely, people who have had an injection of pyruvate in previous studies

developed: unusual taste within the mouth, headaches, flushing, diarrhoea or dizziness after the injection. These side effects were mild and short lasting. Although it is unlikely that an allergic reaction or other side effect will occur, there are facilities in place within the MRI unit, and within the hospital, to manage these appropriately. If you have any questions about your scan, please speak to your doctor.

Computerised Tomography (CT) scan

You may have a CT scan during the screening period (depending on how recent your last one was) and, if required, approximately 3 days before your surgery (you will be told in advance if this includes you). You will also have one just before your 3 month follow up visit. CT scans are used to take pictures of, measure and assess cancer tumours.

When you have the scan, we will ask you to lie still on a couch which will slide through the scanning machine. The scan lasts about 30 minutes, sometimes longer. You may need to drink or have an injection of a contrast agent into a vein which improves the quality of the scan images. You may however be observed for some time after the CT scan to check for a reaction to the contrast agent, which is very rare. Some common side effects that you may experience is feeling hot. Some uncommon side effects include feeling sick, increased sweating, feeling cold, and dizziness. Allergic reaction symptoms can include mild itching or hives (small bumps on the skin) to shortness of breath and swelling of the throat or other parts of the body. You should tell a staff member immediately if you experience any of these symptoms so you can be treated promptly

Some CT scans are part of standard care and you would have had these anyway, whether you take part in the trial or not. Participation in this trial may mean that you receive 1-3 additional CT scans compared with normal care, depending on your result. These procedures use ionising radiation to form images of your body. Ionising radiation can cause cell damage that may, after many years or decades, turn cancerous. The chances of this happening to you as a consequence of taking part in this trial are approximately 0.3 %. If you are concerned about the level of radiation you are having or have had please talk to your trial team.

Tumour biopsy

You will have a tumour biopsy (removal of a sample of tumour tissue with a special needle) during the screening period. The sample will be around the size of two long grains of rice used to diagnose that you have the type of kidney cancer (clear cell) which may confirm that you are eligible for WIRE. The biopsy will be performed in hospital. A type of imaging (e.g. ultrasound) may be used to help guide the needle and reduce the risk of complications. You will receive a sedative to make you comfortable throughout the procedure. The total duration of the tumour biopsy is 30 minutes, plus an observation period of 6 hours. This period is to manage any side effects appropriately. There may be pain, discomfort and/or bleeding as a result of a biopsy.

This is a research procedure for most participants, performed after your DCE-MRI scan.

Routine Blood and Urine sampling

We will ask you to give blood and urine samples for safety purposes throughout the trial to ensure that you are well enough to receive the trial drugs, and to monitor for any trial drugs side effects that you may be suffering from. Please see '*Research biosamples*' for a description of the amount of blood and urine that we are taking.

Kidney Surgery (Nephrectomy)
The nephrectomy (surgical removal of a kidney) or partial nephrectomy (removal of the tumour from the kidney) will be performed by your hospital. The kidney surgery is standard of care and should not be affected as a result of taking part in this trial. It is important that you inform the trial team of any side effects as early as possible to ensure they do not affect your surgery should you not suffer serious conditions causing a delay.

Research biosamples
We will ask you to donate extra blood, urine and tissue samples for research related to this trial. These samples will be used to gain more detailed information on how your body and the cancer cells are affected by the trial drugs, and to give further details regarding the biology of the cancer.

The specific details of the research samples taken during this trial are given below.

Research Blood samples
The total amount of extra blood we will take from you for research related to this trial depends on the treatment schedule you are allocated to. We will take a maximum of 31mls of blood at each appointment (roughly 6 teaspoons). Of this 31mls, approximately 13mls will be used for your routine lab tests, and 18mls will be used for research purposes. These blood samples will be taken alongside routine bloods wherever possible. You may experience some discomfort when they are taken. These are timed as follows:

- During the screening period (Routine + Research - 31mls).
- On Day 1 (Routine ONLY - 13mls).
- On Day 15 (Routine + Research - 31mls).
- On the visit that takes place 3 days before surgery (Routine + Research - 31mls).
- At the End of Trial Follow-up visit, 3 months after surgery (Routine + Research - 31mls).

Research Urine samples
We will ask you to give extra urine for research related to this trial, and we may also use these samples to investigate the broader biology of the cancer. You will need to have fasted for these samples- your Research team will advise you regarding the requirements.

Research Tumour Tissue samples
During your nephrectomy, extra small samples of tumour and normal kidney tissue will be taken for research just prior to disconnecting the kidney from its blood supply. This procedure is extra and therefore would not normally be done. It will likely only add around 1 minute to your surgery. Each extra sample we take will be around the size of two long grains of rice put together lengthways, end-to-end. We will aim to take 6 samples. There is an additional risk of bleeding from this biopsy, which is predicted by the Investigators to be very small as your kidney will be removed moments later. This biopsy is important for the research: By looking at this sample (taken from this kidney whilst it still has blood flow), researchers can get the fullest picture of its activity following the drug treatment.

Following the nephrectomy, in the pathology department, the pathologist will take extra tissue from the kidney tumour and normal kidney tissue for research.

10. Are there any additional requirements?

If you agree to take part in this trial, there are a number of things that you must and must not do.

Hospital appointments, tests and assessments

You will need to attend all of the trial hospital appointments arranged by your trial team and agree to the tests needed for this trial.

Other medications

There are certain medications which you cannot take whilst you are receiving the trial drug(s) as the interaction between the medication and the trial drug(s) is not yet known. You must inform your trial doctor of any medications, over the counter medications, supplements, herbal remedies or alternative therapies you are taking or using. Your trial doctor will inform you if any changes are needed. You will also need to check with your trial team before taking any newly prescribed, over the counter medications, supplements, herbal remedies or therapies.

Other clinical trials

Whilst you are receiving the trial drugs you will not be able to participate in any other interventional research studies. It is possible that your participation in this trial may stop you being able to take part in some clinical trials in the future, depending on the nature of that trial.

Dietary restrictions

It is prohibited to consume grapefruit juice or Seville oranges if you are taking olaparib.

Contraception, pregnancy and breastfeeding

Please also share this information with your partner if it is appropriate.

Trial medicines can harm an unborn baby or nursing infant. You will not be able to take part in this trial if you are pregnant or breastfeeding. You should also not participate in this trial if you are planning to become pregnant or father a child during the trial or for six (6) months after the completion of trial treatment.

Women of childbearing potential are required to use two highly effective forms of contraception for the duration of the trial (from signing the informed consent form until end of trial treatment) and for three (3) months after the completion of the trial treatment.

Acceptable non-hormonal birth control methods include:

- Intrauterine Device (IUD) PLUS male condom provided coils are copper-banded.
- True abstinence (where the participant refrains from any form of sexual intercourse and is in accordance with the participant’s preferred and usual lifestyle).
- Vasectomised sexual partner PLUS male condom (with participant assurance that the partner has received post-vasectomy confirmation of azoospermia).
- Tubal occlusion PLUS male condom.

Acceptable hormonal birth control methods include:

- Hormonal shot or injection (e.g. Depo-Provera) PLUS male condom.
- Etonogestrel implants (e.g. Implanon, Norplant) PLUS male condom.

- Intrauterine system (IUS) device (e.g. levonorgestrel releasing IUS – Mirena) PLUS male condom.

Men and male partners are also required to use adequate contraception for the entire duration of the trial and for three (3) months after the completion of the trial treatment. This includes:

- Barrier contraception (condom and spermicide) even if female partner(s) are using another method of contraception or are already pregnant (also to protect male partners from exposure to the trial medicines).
- True abstinence (where this is in accordance with the participant's preferred and usual lifestyle).

Male participants should refrain from donating sperm for the duration of the trial and for three (3) months thereafter.

The risks of the trial drugs to the unborn child are currently unknown, so we would like to follow any pregnancies that occur to yourself or partner, during trial treatment or up to three (3) months after you complete trial treatment, up until the birth. This is so that we can learn more about the safety of the trial treatment.

If you or your partner becomes pregnant during the trial or within three (3) months of stopping treatment, you should inform your trial team immediately. Your trial team will discuss all the options available to you. The outcome and progress of any pregnancy will be followed by your trial team, (with your/your partner's consent), and we will ask you questions about the pregnancy and baby, if appropriate.

For Women of Child Bearing Potential: At the bottom of this document is an optional consent section. This is so that you can consent to the collection of data relating to your pregnancy, should a pregnancy occur.

For Male Participants: Should a pregnancy occur to your partner, your partner will be approached separately with information regarding the treatments you have received, and asked whether they would be willing to give consent for their pregnancy to be followed up.

<u>Private insurance policies</u>
You should discuss your participation in this trial with any insurance provider you have (e.g., protection insurance, life insurance, income protection, critical illness cover and private medical insurance) and seek advice if necessary, as failure to notify them may affect or invalidate your cover.

11. What are the side effects of the drug being tested?

Each person's reaction to a new drug is different. Some people have very few side-effects, while others may experience several. You will be closely monitored throughout the trial, but you should inform the trial team if you feel unwell or different in any way. If you have any major concerns or are feeling very unwell please contact your trial doctor immediately using the contact numbers at the end of this information sheet. Informing the trial team about any side-effects will help them manage your care, and they may be able to prescribe medication to ease any side effects. We also need to know so that the potential side-effects of this new combination can be recorded. A break from treatment may be necessary if you are experiencing side effects.

<table>
<tr><th>Side Effect</th><th>Olaparib</th><td rowspan="14">This table displays the side effects noted by the drug or drugs that you will receive.

Very Common side effects typically affect more than 1 in 10 people

Common side effects typically affect less than 1 in 10 people</td></tr>
<tr><td>Abdominal Pain</td><td>Very Common</td></tr>
<tr><td>Blood Result Abnormalities</td><td>Very Common</td></tr>
<tr><td>Cough</td><td>Very Common</td></tr>
<tr><td>Decreased Appetite</td><td>Very Common</td></tr>
<tr><td>Diarrhoea</td><td>Very Common</td></tr>
<tr><td>Digestive problems</td><td>Very Common</td></tr>
<tr><td>Dizziness and/or Sickness</td><td>Very Common</td></tr>
<tr><td>Headache</td><td>Very Common</td></tr>
<tr><td>Skin problems (itching, rashes)</td><td>Common</td></tr>
<tr><td>Kidney function changes</td><td>Common</td></tr>
<tr><td>Mouth complications (dry or sore)</td><td>Common</td></tr>
<tr><td>Shortness of breath</td><td>Very Common</td></tr>
<tr><td>Tiredness and/or Weakness</td><td>Very Common</td></tr>
</table>

Uncommon side effects

In rarer instances (fewer than 1 in 100 people), other symptoms of taking these drugs have been noted that you should keep in mind before you agree to participate in the WIRE trial. Below these rare side effects are listed for the drug that you may be allocated, should you take part;

Olaparib

- Hypersensitivity reaction
- Itching and redness of the skin with or without a rash (dermatitis/eczema)
- Increase in volume of red blood cells (mean cell volume (MCV) elevation)

Section 2: Conduct of the trial

12. What if new information becomes available?

Sometimes during the course of a trial, new information becomes available which might affect your decision to continue participating in this trial. Your trial team will contact you to discuss the new information and whether you wish to continue participating in the trial. If you still wish to continue on the trial, we will ask you to sign a new Informed Consent Form.

The trial sponsor, the regulatory authority or the trial doctor may decide to stop the trial at any time. If that happens we will tell you why the trial has been stopped and arrange for appropriate care and treatment for you.

13. What if I decide I no longer wish to participate in the trial?

You can stop participating in this trial at any time without giving a reason and without affecting your future care or medical treatment. If you decide not to participate any further, you will no longer receive the trial drugs. No further tests will be performed on you and no further research samples will be collected. Any data or results already collected will continue to be used in the trial analysis. Your trial team may also choose to withdraw you from the trial if they feel it is in your best interests or if you have been unable to comply with the requirements of the trial. Possible reasons for withdrawing you could include:

- Not taking the trial drugs as required
- Experiencing a serious side effect (your doctor will follow-up regarding your progress until this side effect has stabilised or resolved)
- Your disease gets worse whilst receiving trial treatment
- Finding out that you are not eligible after completing the screening tests
- Being unable to complete the visits or trial documentation as required
- Becoming pregnant or planning to become pregnant
- The trial doctor feels you no longer appear to benefit from the trial drugs.

14. What if there is a problem?

Any complaint about the way you have been dealt with during the trial or any possible harm you might suffer would of course be listened to and addressed wherever appropriate. If you have any concerns about any aspect of this trial you should speak to your trial doctor who will do their best to answer your questions.

In the event that something does go wrong and you are harmed by taking part in the research and this is due to someone’s negligence then you may have grounds for a legal action for compensation against Cambridge University Hospitals NHS Foundation Trust or the University of Cambridge. The normal National Health Service complaints mechanisms will still be available to you (if appropriate). The University has obtained insurance which provides no-fault compensation, i.e., for non-negligent harm, you may be entitled to make a claim for this.

If you wish to complain or have any concerns about any aspect of the way you have been approached or treated during this trial, you can do this through the NHS complaints procedure. In the first instance it may be helpful to contact the (to be completed locally as appropriate – in England this will refer to the Patient Advice and Liaison Service (PALS)) at your hospital.

15. Will my taking part in this trial be kept confidential?

Cambridge University Hospitals NHS Foundation Trust (CUH) and The University of Cambridge are the Sponsors for this clinical trial based in the United Kingdom. They will be using information from you and your medical records in order to undertake this trial and will act as the data controller for this trial. This means that they are responsible for looking after your information and using it properly. The Sponsor organisations will keep identifiable information about you for 5 years after the trial has finished to ensure your safety and allow the trial to be reviewed by the authorities after it is finished.

Your rights to access, to change or to move your information are limited, as the Sponsor organisations need to manage your information in specific ways in order for the research to be reliable and accurate. To safeguard your rights, we will use the minimum personally-identifiable information possible.

You can find out more about how the Sponsor (s) use(s) your information using the information below:

- For Cambridge University Hospitals NHS Foundation Trust, please visit: https://www.cuh.nhs.uk/corporate-information/about-us/our-responsibilities/looking-after-your-information

Or email the Data Protection Officer at: gdpr.enquiries@addenbrookes.nhs.uk

- For University of Cambridge, please visit:
https://www.medschl.cam.ac.uk/research/information-governance
Or email the Information Governance team at: researchgovernance@medschl.cam.ac.uk

For participants recruited at CUH (where the Sponsor is also the site):
Cambridge University Hospitals will collect your name, NHS number and contact details to contact you about this trial and make sure that relevant information about the trial is recorded for your care and to oversee the quality of the trial. Individuals from the Sponsors and regulatory organisations may look at your medical and research records to check the accuracy of this trial. Cambridge University Hospitals will pass these details to the Sponsors along with the information collected from you and/or your medical records. The only people in the Sponsor organisations who will have access to information that identifies you will be people who need to contact you in relation to this trial and to audit the data collection process. Cambridge University Hospitals will keep identifiable information about you from this trial for *5* years after the trial has finished.

For participants recruited at other participating sites:
(Add site name) will keep your name, NHS number and contact details to contact you about this trial, and make sure that relevant information about the trial is recorded for your care, and to oversee the quality of the trial. Certain individuals from the Sponsors, and regulatory organisations may look at your medical and research records to check the accuracy of this trial. The Sponsor(s) will only receive information without any identifying information. **(Add site name)** will keep identifiable information about you from this trial for *XX* years after the trial has finished.

All information collected about you as a result of your participation in the trial will be kept strictly confidential. Your personal and medical information will be kept in a secured file and be treated in the strictest confidence.

Once you have agreed to participate in this trial you will be allocated a Trial ID Number. This is a unique trial number which will be used on all your trial documentation along with your date of birth. Your date of birth is considered to be personal information. We collect this personal information on trial documentation to help ensure that the data we receive as part of your trial participation is correctly allocated to you. By cross checking these two unique references we can ensure the integrity of the data.

When you agree to take part in this trial, the information about your health and care may be provided to researchers running other research studies in this organisation and in other organisations. These organisations may be universities, NHS organisations or companies involved in health and care research. Any serious side effects that occur during the trial will also be shared to the manufacturer of the trial drugs (AstraZeneca) using your trial-specific identifiers. The statistics for this trial is being undertaken by the University of Newcastle. All data that is shared with the University of Newcastle will be identified using only your trial ID and date of birth.

Your information will only be used for the purpose of health and care research, and cannot be used to contact you or to affect your care. It will not be used to make decisions about future services available to you, such as insurance.

Your coded trial data may be sent to other country(ies) outside the European Economic Area (EEA) for analyses, where the data protection laws are not the same. Your coded personal data may also be shared with off-site licenced laboratories for the purpose of analysing samples collected from you in this trial. However this information will not identify you and will not be combined with other information in a way that could identify you. The people who analyse the information will not be able to identify you and will not be able to find out your name, NHS number or contact details. Only anonymous trial data, without any personal information will be published at the end of the trial.
We will need to inform your GP of your participation in this trial. This is so that any medical decisions that your GP makes will take into account the fact that you are receiving drugs as part of this trial.

16. What will happen to my samples and scan images?

With your permission, we will analyse the blood, urine, kidney and tumour samples collected from you during this trial. This analysis will take place over a long period of time, so any results obtained will not alter your care pathway.

If you decide to stop taking part in the trial please tell the trial doctor if you want to change your mind about us using your donated sample(s) to look at the molecular and genetic makeup of the cancer.

<u>Routine blood samples</u>
Blood samples taken will be analysed immediately by your hospital and will be destroyed once the laboratory tests are complete. The results will include those you would receive as part of your standard care pathway, plus any results required for the trial. These results will be accessible to your trial team.

<u>Research samples</u>
Research samples taken will be labelled with your Trial ID Number, processed and stored initially by your trial team at your hospital, before being transferred to an approved facility for analyses. The approved facilities are either part of the Cambridge Biomedical Campus or

AstraZeneca. These labs may be inside or outside of the UK. The samples will be securely stored and analysed by specialist teams. You will not be identifiable to researchers from any of the laboratories we may use for research analysis.

These samples will be analysed in a way which will tell us whether WIRE trial treatments are eliciting the biological response that we hope and expect to see. There may be other tests that we perform on your research samples to give us additional information regarding the drug activity.

Once the research analysis is complete any remaining samples will either be:

- Stored by the research team in line with the relevant regulatory requirements, in an appropriate facility pending ethical approval for use in a future trial outside of the WIRE trial.
- Will be disposed of in accordance with the UK Human Tissue Authority code of practice.

<u>Archival tumour</u>
You may have previously had a biopsy of your kidney, and therefore have more than one tumour sample. Not all patients will have undergone a previous biopsy. If you have however, we will ask your permission to access this 'Archival' tissue from your local hospital, so that we can compare the changes with your recent biopsy sample. This will also help us in the event that your most recent sample isn't big enough for us to perform our analysis. Granting us permission to access these samples is entirely optional.

<u>Genetic Testing (All samples)</u>
All of our cells in our body get information on how to work and function through a molecule called deoxyribonucleic acid (DNA). DNA is a record of instructions where the instructions are called genes. Genetic testing looks at your genes and could either look at one or some specific genes or all of your genes – your whole DNA. Genes affect how we grow and develop. Nobody else has exactly the same genes as you do, unless you have an identical twin. These differences mean that some people are more likely than others to get certain diseases and they also mean that medicines affect people differently. Some genes can be important in more than one disease.

Genetic testing in this trial is only for research purposes, but is an essential part of the trial. If you do not want to agree it to, you will be unable to take part. These genetic tests will be performed on any samples you donate to the trial, and requires no additional time from you.

The trial team may also undertake laboratory tests using your samples that reenact the environment of natural body conditions in a laboratory setting - so called '*ex vivo* modelling'.

This method of analysis typically provides very meaningful data regarding how cells interact naturally with the drugs received. Tissue cells are grown in a laboratory environment and could be modified to implant in mice with low resistance to disease. The laboratory staff will monitor the growth of the tumour (derived from your own) within the mice. Any laboratory performing this analysis would have the appropriate license for testing on mice. You have the right to take part in the trial without these particular lab procedures taking place. If you give your optional consent for these procedures, you may withdraw your consent for your samples to be used in this way at any point until this laboratory analysis begins.

Cambridge University Hospitals NHS Foundation Trust & the University of Cambridge may do this testing in its own laboratories or it may be done in the laboratories of other organisations (such as universities, collaborators and genetic specialist), or both. Results

from this research may be shared and used as described in 'Will my taking part in this trial remain confidential?' In addition Cambridge University Hospitals NHS Foundation Trust & the University of Cambridge, and its designated organisations may conduct future research where they will share summary results (not your individual results) or anonymous datasets with other researchers, from other companies or universities, for example. The results may be combined with the results of other studies, for example as part of scientific databases. The researchers can only use this information for medical and healthcare related research.

Radiological (Scan) Images

The images obtained by way of CT and/or MRI scans will be coded, using only your trial ID number and Date of Birth. These coded images will then be sent on a secure CD, through the post, to the Sponsors (Cambridge University Hospitals and the University of Cambridge). These images will be stored on password-protected computers at Cambridge University for the duration of the trial.

CT scans will be used by your doctor to monitor general size changes to the tumour, and will likely be kept at your local hospital, but the central trial team may request them for checking of the measurements provided by your hospital. The people performing the analysis on your MRI images will not be able to identify you. They will be experienced Radiologists that will be able to identify areas of the cancer to get a general idea of how the cancer is behaving; so called 'Regions of Interest'. By using specialist software, the Radiologists can measure the amount of blood flowing through the cancer. This analysis will be part of the assessment of your MRI scan performed before your treatment, and after your treatment for comparison.

The anonymised images may support future research projects, for example finding new ways of gaining even more insight into renal cancer. Therefore, we ask for your permission to use these images, which do not identify you personally, in other future research projects.

17. What will happen to the results of the trial?

The results of the trial will be anonymous and you will not be able to be identified from any of the data produced. When the results of this trial are available they may be published in peer reviewed medical journals and used for medical presentations and conferences. They will also be published on the EU Clinical Trials Register website, a central registry for all clinical trials conducted in the EU.

Anonymised datasets from the trial will be made available to the funder (AstraZeneca), and may also be made available to other researchers in line with national and international data transparency initiatives.

If you would like to obtain a copy of the published results please contact your trial doctor directly who will be able to arrange this for you.

18. Who is funding the trial?

The trial is being funded by AstraZeneca and the Cancer Research UK Cambridge Centre. AstraZeneca is also supporting this trial by providing the trial drugs free of charge.

The trial is being managed by the Cambridge Clinical Trials Unit – Cancer Theme. The Chief Investigator is Mr Grant Stewart who is a University Lecturer at the University of Cambridge and Honorary Consultant Urological Surgeon at CUH.

19. Who has reviewed this trial?

All research within the NHS is reviewed by an independent group of people called a Research Ethics Committee, to protect your interests. This trial has been reviewed and given

favourable opinion by the (name of REC here). The Medicines and Healthcare Products Regulatory Agency (MHRA) who are responsible for regulating medicines in the UK have also reviewed this trial.

20. Further information and contact details

If you have any questions concerning this trial please contact your:

Trial Doctor
Name: [INSERT your hospital contact details here]
Tel: [INSERT your hospital contact details here]
Research Nurse
Name: [INSERT your hospital contact details here]
Tel: [INSERT your hospital contact details here]

In the event of an emergency please contact:

24-hour contact details
Contact: [INSERT your hospital contact details here]
Tel: [INSERT your hospital contact details here]

Outside of an emergency,
You may wish to contact one of the following organisations that are independent of the hospital at which you are being treated:

CancerHelp UK is a registered charity providing information about all aspects of cancer. It can provide useful information on cancer treatments and medical research. You can contact their nurses on freephone 0808 800 4040. You can also access their web site at: www.cancerresearchuk.org

MACMILLAN Cancer Support is a registered charity providing information about all aspects of cancer. They have published several useful booklets on different types of cancer, chemotherapy, radiotherapy, and clinical trials in general. You can contact the nurses on freephone 0808 808 0000. You can also access their website at www.macmillan.org.uk

If you wish to complain or have any concerns about any aspect of the way you have been approached or treated during this trial, you can do this through the NHS complaints procedure. In the first instance it may be helpful to contact the Patient Advice and Liaison Service (PALS) at your hospital.

Patient Advice and Liaison Service (PALS) at:
Hospital Name: [INSERT your hospital PALS contact details here]
Tel: [INSERT your hospital PALS contact details here]

TO BE PRINTED ON HEADED PAPER

UNIVERSITY OF CAMBRIDGE

Cambridge University Hospitals NHS
NHS Foundation Trust

INFORMED CONSENT FORM

Trial Title: WIRE: WIndow-of-opportunity clinical trials platform for evaluation of novel treatments strategies in **RE**nal cell cancer.

Principal Investigator: [INSERT your PI name here]

Participant Number: ___________ (please add once the participant is registered)

**If you agree with each sentence below, please initial the box**		**INITIALS**
1	I have read and understood the Group 3, Olaparib only, Participant Information Sheet version 3.0, dated 10th February 2020 for the above trial and I confirm that the trial procedures and information have been explained to me. I have had the opportunity to ask questions and I am satisfied with the answers and explanations provided.	
2	I understand that my participation in this trial is voluntary and that I am free to withdraw at any time, without giving a reason and without my medical care or legal rights being affected. I also understand that data or samples already collected will continue to be used in the trial analysis.	
3	I understand that personal information about me will be collected and used in accordance with this information sheet. This information will be kept in the strictest confidence and none of my personal data will be published.	
4	I understand that sections of my medical notes or information related directly to my participation in this trial may be looked at by responsible individuals from the sponsor, regulatory authorities, research personnel where it is relevant to my taking part in research and that they will keep my personal information confidential. I give permission for these individuals to have access to my records.	
5	I understand that my GP will be informed of my participation in this trial and sent details of the WIRE trial.	
6	I have read and understood the compensation arrangements for this trial as specified in the Participant Information Sheet.	
7	I understand that the team/doctors in charge of this trial may close the trial or stop my participation in it at any time without my consent.	
8	I have read and understood my responsibilities for the trial including using appropriate contraception as listed in section 6.	
9	I agree to give blood and urine for research related to this trial.	
10	I agree to give tumour and healthy tissue samples for research related to this trial	
11	I give my permission to allow my kidney tissue and research blood, urine & tumour samples to be sent to specialist teams on the Cambridge Biomedical Campus, or to laboratories contracted and approved by the Sponsor(s) for tests/analysis related to this trial. I understand that only the minimum information will be supplied and that I will not be identifiable to any of the researchers.	
12	I understand that the results from the analysis carried out on the research blood and tumour samples will not be fed back to me.	
13	I understand that the information collected about me will be used to support other research in the future, and may be shared anonymously with other academic and commercial researchers external to the project, within the UK and beyond	

14	I give my permission to allow for DNA testing of my samples		
	**OPTIONAL:** Please read each statement below and initial the relevant box	**INITIALS**	
		Yes	No
15	I give my permission to allow for any archival tumour tissue to be collected for tests related to this trial. I understand that only the minimum information will be supplied and that I will not be identifiable to any of the researchers.		
16	I give my consent for my tissue samples to be used to grow cell cultures or be implanted into mice		
17	I give my permission to allow any remaining research blood and tumour samples taken as part of this trial to be retained in an HTA licenced facility pending Ethical Approval for use in another project at the discretion of the Chief Investigator. I understand that these samples will be retained in an approved storage facility, that only the minimum information will be supplied and, that I will not be identifiable.		
18	I give my permission for my anonymised radiological images to be used as part of the data for future research studies		
	**FOR WOMEN OF CHILDBEARING POTENTIAL ONLY**<br>**OPTIONAL:** Please read each statement below and initial the relevant box	**INITIALS**	
		Yes	No
19	If I become pregnant during, or in the three (3) months after receiving the trial drugs, I agree to information being collected about me, my pregnancy and my baby.		
20	I understand that sections of my medical notes or information related directly to my pregnancy may be looked at by responsible individuals from the sponsor, regulatory authorities and research personnel where it is relevant to my taking part in research. I give permission for these individuals to have access to my records.		
21	I agree to give my pregnancy information voluntarily and understand that I am free to withdraw at any time without giving a reason and without my medical care or legal rights being affected. I understand that all data collected up to the withdrawal of consent will be kept confidential.		

I agree to participate in this trial:

Name of participant	Signature	Date
**Name of person taking consent**	**Signature**	**Date**

Time of consent (24hr clock): _______:_______

1 copy for the participant, 1 copy for the trial team, 1 copy to be retained for hospital records

TO BE PRINTED ON HEADED PAPER

TO BE PRINTED ON HEADED PAPER

Group 4 – Durvalumab Only

PARTICIPANT INFORMATION SHEET & INFORMED CONSENT FORM

WIRE: WIndow-of-opportunity clinical trials platform for evaluation of novel treatment strategies in **RE**nal cell cancer.

You are being invited to take part in a research trial. Before deciding whether to take part, you need to understand why this research is being done and what it involves.

Please take time to read the following information carefully and talk to others about the trial if you wish. Please ask your trial team if anything is not clear or if you would like more information. Please take time to decide whether or not you wish to take part. You will be receiving this Participant Information Sheet before any diagnosis has been made i.e. it is not yet confirmed that you have renal cell cancer.

Section 1 tells you the purpose of this trial and how the trial would involve you, including a description of what the various tests are, and some of the risks and restrictions of taking part

Section 2 gives you more detailed information about the trial process, including information about how the trial is run, and how we may use your data.

Section 1: Purpose of the trial and what will happen

1. What is the purpose of the trial?

Kidney cancer is the 7th most common cancer in the UK. Currently many patients with this cancer are offered a kidney removal operation (known as nephrectomy), which cures many patients with no further need for treatment. However, some may receive drug treatment after surgery.

WIRE is a trial that will administer drugs to patients in the time between the decision to operate and the nephrectomy operation. Patients are not usually given anti-cancer drugs during this period, and indeed may never need them, but it could give researchers important information to guide the best combinations of anti-cancer drugs to prioritise for later phase clinical trials. The trial will investigate the effect and safety of 3 different cancer drugs, individually or in combination, in patients with a specific type kidney cancer called clear cell renal cell cancer. The cancer may be localised (within the kidney) or metastatic (it has spread outside of the kidney).

2. What are the drugs being tested?

There are 3 drugs being used in this trial, Cediranib, Olaparib and Durvalumab. All three of these drugs target different parts of cancerous cells which might decrease the size of the tumour over a period of time. However, the duration of treatment in this trial is very short in comparison to how long these drugs would normally be given for. Therefore, it is unlikely that the trial treatment will noticeably reduce the tumour size if it is confirmed that you do have kidney cancer. . In this trial, we will primarily be looking at cancer's response to these drugs by taking a closer look at the tumour cells in the laboratory and by using new imaging technology.

Although the trial uses 3 drugs, we will only be giving you one or two of these drugs. The drugs you are given will depend on the group you are allocated. The trial team already know which group you will be allocated. Currently, we are recruiting participants for the **Durvalumab** group. Each group has its own specific entry criteria which may involve different tests and assessments; this is discussed further under *Section 4 Do I have to take part?*

Durvalumab is an immunotherapy drug which enables a person's immune system to work better against cancer cells. It is a type of targeted anti-cancer drug called a monoclonal antibody (mAb) which binds to certain cells or proteins. Durvalumab seeks cancer cells by looking for a particular protein and attaching to it. By doing this durvalumab may help the individual's immune system attack the cancer and stop it from growing. Durvalumab is being tested in clinical trials, but is currently only licenced in the EU for use in patients with a type of lung cancer. This drug is given as a once-only, hour-long infusion in hospital via a cannula (a needle with a small plastic tube) placed into a vein in your hand or arm. This infusion will take place at least 14 days before your surgery.

3. Why have I been invited?

You have been invited to participate in this trial because you may have renal cell cancer (kidney cancer) that may not be cured with surgery alone. The diagnosis of renal cell cancer will be confirmed or disproved with a pre-surgery biopsy. In normal care, usually your treating doctor will recommend proceeding with a nephrectomy or partial nephrectomy (removal or partial removal of the kidney) without the need of a pre-surgery biopsy. However, as part of the WIRE trial, participants will be required to undergo a biopsy during the screening stage to determine whether they have the type of cancer we are investigating.
We plan to include up to 76 patients with kidney cancer from approximately 2 hospitals across the UK.

4. Do I have to take part?

Participating in this trial is completely voluntary. If you decide to participate you will be asked to sign an Informed Consent Form. You are however still free to change your mind and leave the trial at any time and without giving a reason. If you chose not to participate or to leave the trial, your current and future medical treatment will not be affected in any way. Your normal standard care will not be affected in any way.

You should also be aware that entry into this trial requires you to meet some strict entry criteria that your doctor will assess you for over the next few weeks, during a period called 'screening'. At the moment we will not know whether you are eligible for the trial as there are further tests and assessments to do, but some people who consent to take part in this trial may not meet the entry criteria to receive the trial drugs.

Before proceeding, it is important to understand that you may not benefit from the extra drugs and procedures involved in this trial. Surgery alone may be sufficient to cure the cancer alone, although there is no way to predict this for certain. The possible side effects could potentially delay your surgery, and participation in the trial could make you feel

fatigued and nauseous. However, your doctors will be monitoring how you are feeling very closely, making every attempt to minimise any side effects.

It is also important you are aware that based on previous research the average chance of being cured by surgery alone needing no further treatments is 42%, in whom no further treatments would be needed. It is also important that you are aware that you will not have access to the trial drugs after your operation, and you might not be permitted to take part in other clinical trials if you decide to participate in the WIRE trial.

5. Expenses and payment

You will not receive any payment for participating in this trial however we can reimburse reasonable travel and parking costs incurred by your participation in this trial. This is up to a maximum of £180 for the entire duration of your participation in this trial. We will ask you to retain your receipts wherever possible.

6. What are the possible advantages and disadvantages of taking part?

It is unlikely that you will benefit clinically from taking part in this trial, although it is possible that the tumour may shrink very slightly. It is also hoped that taking the medications used in the trial could provide you with a better outcome, but this cannot be directly measured in this trial. You will gain access to drugs that are only available within a clinical trial. You are likely to have more contact and for longer with your care team than if you were not involved in a trial, and some patients gain reassurance from this. It is hoped that information collected as part of your participation in this trial will benefit patients with kidney cancer in the future and you may contribute to their future treatment and care. Your care team will be able to share information with you regarding how the tumour cells responded and/or any tumour size reduction, if you wish to know this.

In addition to the above benefits there are also aspects of your participation you may find difficult. For instance, if you decide to take part:

- You will need to undergo a biopsy at the screening stage which is likely to require a full day at the hospital. It is also likely that this biopsy would not have been required if you were not participating in this trial. This is an extra procedure for about 70% of patients. You can find more information about this under section 9 – Tumour biopsy on page 12.
- There will be an increased number of hospital visits due to participating in the trial. You will be required to come to the hospital an extra 4-5 times during the trial. These visits are to conduct initial assessments and scans (1 extra visit); clinic visits whilst receiving the trial drug(s) (2-3 extra visits); a visit for the scan before surgery (1 extra visit). The average length of time per visit is about 2 or 3 hours, but actual time may vary. The schedule and timing of these visits is available on page 9.
- You will undergo at least an additional 2 MRI scans if you decide to take part in this trial, with potentially another 2 MRI scans using a new type of scan called a ‘hyperpolarised MRI’ if available at your hospital (see section 9, pages 11 and 12 for more information). An additional 1-2 CT scans may be required as recommended by the doctor based on information from your diagnosis and previous scans. You would not have normally received these scans if you were not participating on the trial.
- You could experience drug side effects such as nausea, fatigue, diarrhoea, dizziness, decreased appetite and blood test abnormalities. A full list of known

side effects is listed on pages 15 to 16. It is also possible that some of these side effects might delay your surgery.

- There will be 6 additional tissue biopsies (samples) taken during your surgery, this will likely only add a few minutes to your standard surgical procedure. Whilst these are unlikely to impact you or prolong your stay in hospital, it is important that you are aware these are taken. More information about these tissue samples is mentioned on page 13.

  The drugs you will be taking as part of this trial will not be given to you after the surgery, and may prevent you from taking part in other clinical trials after your surgery.

7. What are the alternatives for treatment?

The standard care for you is a nephrectomy (removal of the kidney) or partial nephrectomy (removal of the tumour only) without any drug treatment before surgery.

8. What happens when the trial stops?

Following completion of the trial you will not be able to receive any further trial drug treatment and we will refer you back to your primary consultant. You will still be able to contact your trial Nurse or Practitioner, however, with any questions about the trial.

9. What will happen to me if I take part?

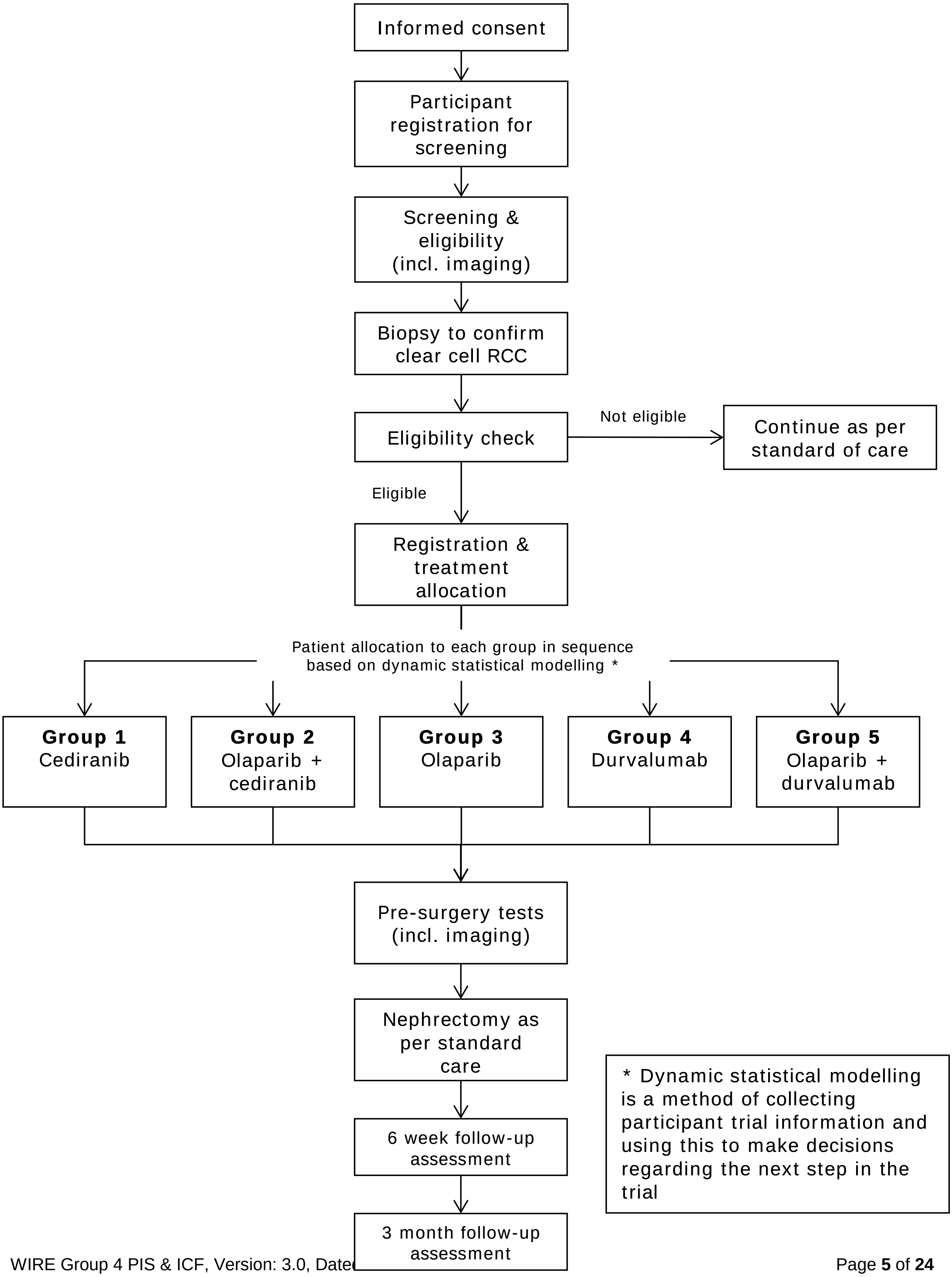

Hospital Appointments

Informed Consent

If you agree to participate in the trial, you will be invited to sign the Informed Consent Form at the end of this document. You will be given a copy of this to take away and refer to later.

Screening and baseline

Standard of care visit plus extra trial assessments 5-6 hours in duration. Screening may require an additional visit to complete all assessments, dependent on hospital capacity.

To check that you meet all of the requirements for entering this trial you will need to have some tests. This is called the "screening period". You will be given appointments to see the trial team for these tests, which will be carried out within a 28 day period. This may include more than one visit to the hospital. All of the tests listed during this period and the following visits are further explained later in this information sheet (see page 5). All of the tests listed under Screening will collect data for the research trial.

- A check of your weight, blood pressure and temperature
- A full physical examination
- Clinical review
- Performance status check
- Blood and urine sampling
- 3 Electrocardiogram assessments (ECGs), 5 minutes apart.
- A minimum of one type of scan, including a Dynamic Contrast Enhanced Magnetic Resonance Imaging (DCE-MRI) scan at least (a type of scan that uses strong magnetic fields and radio waves to produce detailed images of the body and organs). You may undergo a hyperpolarised MRI scan (a new MRI scanning technique which produces extremely detailed images of the body and organs), depending on the group you are allocated to, and the treatment centre you are attending. You may also undergo a CT scan, depending on how long ago your last CT scan took place (the risks of increased radiation exposure are explained further on in this document, in the 'Tests and Assessments' section).
- Collection of your Medical History
- Research blood and urine sampling- we will take these samples before you have the tumour biopsy, on the same day
- Tumour Biopsy
- Pregnancy test (female participants only)

Your weight, blood pressure, temperature, physical examination, performance status check, blood & urine samples, CT and ECGs may be assessed as part of your standard care pathway (these would usually take place without trial participation anyway as part of the usual pre-surgery checks). A tumour biopsy would not normally take place in about 70% of patients, but all participants will have a biopsy in order to be eligible for the trial.

Additional ECGs, a possible repeat CT scan (if you haven't had one within the last 28 days) and all of the other assessments and scans listed are extra for the trial.

Registration

Some of the tests performed during Screening require the results to fall within certain trial ranges. If any of your results do not fall within these ranges, you will not be able to participate in this trial and will be referred to follow the usual treatment pathway for patients with your condition. However, if the screening tests show that you are suitable to participate in this trial, your clinical team will register you into the trial.

There are 5 different treatment groups in this trial, but each participant will only be allocated to one of these groups, and given the appropriate information sheet for the drug that they might receive. If you decide to participate and meet the entry criteria, you will be allocated to Group 4 and be given Durvalumab.

Clinic Visits

Extra visits for the trial, up to 3 hours per visit in duration

Once your participation in this trial has been confirmed we will ask you to attend regular clinic appointments to see the trial team whilst you are receiving the trial drug/s and we will perform a number of trial related tests to ensure you are tolerating the trial drugs well. Initially these will be performed on the 1st and approximately 15th day of your treatment but will be flexible around weekends and bank holidays. You will need to have fasted for 6 hours before the Research urine tests, and your research/care team will advise you regarding the timescales on this. Both of these visits are extra visits for the trial and all assessments for these visits below are needed just for the trial and are outside of standard care:

- A weight, blood pressure and temperature check
- A physical examination (if your doctor believes it is necessary)
- Performance status check
- Clinical review
- At least 1 electrocardiogram assessment (ECG). Based on your results, your trial team may ask you to perform 2 additional ECGs, all 3 taken 5 minutes apart.
- Blood and urine assessments. Pre-treatment (Day 1)
- Pregnancy test (female participants only)
- You will be administered your Durvalumab on your Day 1 visit.

In addition to the assessments above, the following tests will be performed on Day 15 ONLY

- Research blood and urine sampling
- Assessment of any side effects from the medication

Your clinical trials team may decide to give you an additional assessment on approximately the 22nd day of treatment, if you are still taking the trial drug. This may be by telephone or in clinic; this day 22 assessment will be an extra assessment for the trial, and the below tests will be extra assessments for the trial, outside of standard of care:

- Performance status check
- Assessment of any side effects from the medication
- Clinical review

If you experience unmanageable side effects, or if your trial doctor thinks you should not continue, the trial drugs will be stopped and you will return to the normal standard of care provided by the hospital for the treatment of the cancer.

Pre-surgery

Extra visit, up to 6 hours in duration

Within 3 days of your surgery date, you will undergo at least one scan to assess whether the medication you have been taking is affecting the tumour. These are all extra assessments for the trial, outside of standard care, and the visit could take most of a day or more than one day:

- DCE-MRI
- Hyperpolarised MRI (depending on the group you are allocated to, and the treatment centre you are attending)

- CT scan (if the cancer has spread outside the kidney- your Trial Nurse or Practitioner will advise you)
- Research blood and urine sampling (these are outside of standard care and are required for the trial only)
- Routine blood sampling

Surgery
Standard of care visit. Surgical prep plus 3 hours surgery time.
During your nephrectomy, we will obtain tumour samples, as well as samples of the normal kidney tissue next to the tumour. The surgery is standard care but the amount of samples taken will be higher than in standard care.

Follow-up – 6 weeks after surgery
Standard of care visit, up to 2 hours in duration
You will be given an appointment to see the trial team approximately 6 weeks after surgery to ensure that you have recovered from any side effects. This 6 week check is usual for patients who have undergone nephrectomy, and is standard care. During this visit you will have:

- Performance status check
- Clinical review
- Routine Blood sampling
- Pregnancy Test

End of trial follow-up – 3 months after surgery
Standard of care visit, up to 2 hours in duration
You will be given another appointment to see the trial team approximately 3 months after surgery to ensure that you have tolerated the trial drugs well. A CT scan will be performed prior to this visit. Following this visit, you will not need to attend any further trial related visits. This 3 month check is usual for patients who have undergone nephrectomy, and is standard care. During this visit you will have:

- Performance status check
- Clinical review
- Routine blood sampling
- Research blood and urine sampling (these are outside of standard care and are required for the trial only)
- Pregnancy test

What will happen and when?

Tests and Assessments	Screening (up to 28 days prior to Day 1 of treatment)	Treatment phase			Pre-surgery	Surgery	Follow-up	
		**Day** 1 (± 5 days)	**Day 15** (± 5 days)	**Day 22 (Optional)** ± 5 days			6 weeks after surgery ± 7 days	3 months after surgery ± 14 days
Consent	X							
Medical history	X							
Vital signs	X	X	X					
Performance status check	X	X	X	X			X	X
Clinical review	X	X	X	X			X	X
Physical examination	X	If clinically necessary	If clinically necessary					
ECG	X	X	X					
Urine sample	X	X	X				X	X
Blood for routine tests	X	X	X		X		X	X
Pregnancy test	X	X	X					X
Post-menopausal test	X							
Blood for research tests	X		X		X			X
Urine for research tests	X		X		X			X
DCE-MRI	X				X			
Hyperpolarised MRI	If needed				If needed			
CT scan (*your doctor will tell you if needed)	X*				If needed*			X
Tumour biopsy (screening)	X							
Durvalumab Administration		X						
Tissue samples (research)						X		
Full or partial nephrectomy						X		
Total time of visit	5-6 hours over 2-3 visits	2-3 hours over 1 visit	2-3 hours over 1 visit	1-2 hours over 1 visit or 20 min phone call	4-6 hours over 1-2 visits	2-3 hours of surgery	1-2 hours over 1 visit	1-2 hours over 1 visit

Tests & Assessments

You will be monitored carefully by the trial team whilst you are on this trial. Most of the tests in this trial are necessary to give you the trial drugs safely and ensure you are tolerating the trial drugs well. These tests are explained below, including the possible **risks and/or benefits** of each.

Medical history
Your medical history will be reviewed by the trial team during the screening period. This will involve a discussion about your past and present health, lifestyle and any symptoms you may currently have. This is so that the trial team are aware of any pre-existing conditions. The trial team will also record your gender, ethnicity and date of birth. This is to collect demographic data for the trial.

Weight, blood pressure and temperature
The trial team will monitor your blood pressure and temperature in order to properly assess the side effects to your treatment. These indicators will help the doctors to properly diagnose you and to manage your care.

Clinical review
The trial team will regularly discuss and review your general wellbeing, discuss any symptoms and/or side-effects that you may have, and record any medications you may be taking. This is so that the trial team has a full picture regarding your current state of health.

Your trial team may end your participation in this trial if you have severe or unmanageable side-effects to the trial drug/s. If this happens your trial team will follow-up with you regarding your progress until the side effect has stabilised or resolved.

Physical examination
Your trial doctor will perform a physical examination during the screening period and whilst you are receiving the trial drugs. This will ensure that the trial team has a full picture regarding your current state of health.

Electrocardiogram (ECG)
You will be asked to have several ECGs as part of this trial during the screening period and whilst you are receiving the trial drugs. These record an electrical trace of your heart rhythm and are required to confirm that you are fit enough to receive the trial drugs. They will also monitor the health of your heart during the trial. We will ask you to lie down whilst a series of leads are placed on your chest area, wrists and ankles with adhesive pads, these will record a trace of your heart rhythm. This takes a few minutes and is painless. This is to check the health of your heart.

Performance Status check
Your performance status check will track the impact that your condition or treatment has on your day-to-day activities and on your “usual” level of day-to-day functioning. This is assessed by asking you questions about your level of activity around the house or at work (as applicable).

Pregnancy test/ Non-childbearing status (female participants only)
If you are female and of childbearing potential you will be asked to give a blood sample during the screening period **to confirm that you are not pregnant before you can be enrolled into this trial.** You will also have several pregnancy tests during the trial. If you are of non-childbearing potential you may be asked to provide a sample of blood during the

screening period to confirm your non-childbearing status. This is because we do not yet know the effect of the trial drugs on a developing baby (foetus). This is an extra sample but can be taken from the same bottle as the other blood tests. Most female patients would normally have a urine test.

<u>MRI Scans</u>
You will undergo two DCE-MRI scans if you participate in this trial, and may also have two hyperpolarised MRI scans. It is important that you are aware that the MRI scans would not have been required if you were not participating on this trial. You may need an injection of a contrast agent to improve the quality of the scan. The injection is usually given in a vein in your elbow (the same place as a blood test), which may cause discomfort and/or bruising. When you have the scan, you will be asked to lie still on a couch which will slide into a metal tunnel, which some people find this uncomfortable and/or claustrophobic. The scan lasts for about 45-60 minutes. You will also be able to talk to the radiographer whilst you are having your scan, or listen to music if you prefer. If you are anxious in enclosed spaces you may be prescribed a tablet to help you relax before you have the scan.

<u>DCE-MRI scan</u>
We will ask you to have a DCE-MRI scan during the screening period and before your surgery. DCE-MRI is a type of scan that uses strong magnetic fields and radio waves to produce detailed images of the body and organs. We do this to establish how the trial drugs affect blood flow in the tumour and how the cancer is responding to the trial drugs.
It is known that small amounts of these contrast agents may remain in the brain after a scan with these agents, although there is currently no evidence that these small amounts cause any harm and these agents have been used for many decades. These contrast agents are routinely given during a kidney cancer MRI so we can have a clear understanding of the blood supply to the cancer. You may experience some side effects such as nausea, headaches and dizziness which are very common side effects. Other side effects such as vomiting, injection site reaction, laboured breathing, and allergic reactions are less common. You should tell a staff member immediately if you experience any of these symptoms so you can be treated promptly. We will use the lowest dose of the contrast agent required for a clear image and if you have any questions about your scan, please speak to your doctor.

<u>Hyperpolarised MRI</u>
You may also be asked to have hyperpolarised MRI scans during the screening period and before your surgery. These scans will enable researchers to assess the cancer tumours and the effectiveness of trial drugs. Not every participant will receive this scan, depending on the numbers already in the trial and local availability.
The contrast agent to be injected is a breakdown product of sugar called pyruvate. Although hyperpolarized carbon MRI is a new technology, tests to date have demonstrated no significant safety issues. Pyruvate is a naturally-occurring molecule in the body. Rarely, people who have had an injection of pyruvate in previous studies developed: unusual taste within the mouth, headaches, flushing, diarrhoea or dizziness after the injection. These side effects were mild and short lasting. Although it is unlikely that an allergic reaction or other side effect will occur, there are facilities in place within the MRI unit, and within the hospital, to manage these appropriately. If you have any questions about your scan, please speak to your doctor.

Computerised Tomography (CT) scan

You may have a CT scan during the screening period (depending on how recent your last one was) and, if required, approximately 3 days before your surgery (you will be told in advance if this includes you). You will also have one just before your 3 month follow up visit. CT scans are used to take pictures of, measure and assess cancer tumours.

When you have the scan, we will ask you to lie still on a couch which will slide through the scanning machine. The scan lasts about 30 minutes, sometimes longer. You may need to drink or have an injection of a contrast agent into a vein which improves the quality of the scan images. You may however be observed for some time after the CT scan to check for a reaction to the contrast agent, which is very rare. Some common side effects that you may experience is feeling hot. Some uncommon side effects include feeling sick, increased sweating, feeling cold, and dizziness. Allergic reaction symptoms can include mild itching or hives (small bumps on the skin) to shortness of breath and swelling of the throat or other parts of the body. You should tell a staff member immediately if you experience any of these symptoms so you can be treated promptly

Some CT scans are part of standard care and you would have had these anyway, whether you take part in the trial or not. Participation in this trial may mean that you receive 1-3 additional CT scans compared with normal care, depending on your result. These procedures use ionising radiation to form images of your body. Ionising radiation can cause cell damage that may, after many years or decades, turn cancerous. The chances of this happening to you as a consequence of taking part in this trial are approximately 0.3 %. If you are concerned about the level of radiation you are having or have had please talk to your trial team.

Tumour biopsy

You will have a tumour biopsy (removal of a sample of tumour tissue with a special needle) during the screening period. The sample will be around the size of two long grains of rice used to diagnose that you have the type of kidney cancer (clear cell) which may confirm that you are eligible for WIRE. The biopsy will be performed in hospital. A type of imaging (e.g. ultrasound) may be used to help guide the needle and reduce the risk of complications. You will receive a sedative to make you comfortable throughout the procedure. The total duration of the tumour biopsy is 30 minutes, plus an observation period of 6 hours. This period is to manage any side effects appropriately. There may be pain, discomfort and/or bleeding as a result of a biopsy.

This is a research procedure for most participants, performed after your DCE-MRI scan.

Routine Blood and Urine sampling

We will ask you to give blood and urine samples for safety purposes throughout the trial to ensure that you are well enough to receive the trial drugs, and to monitor for any trial drugs side effects that you may be suffering from. Please see '*Research biosamples*' for a description of the amount of blood and urine that we are taking.

Kidney Surgery (Nephrectomy)

The nephrectomy (surgical removal of a kidney) or partial nephrectomy (removal of the tumour from the kidney) will be performed by your hospital. The kidney surgery is standard of care and should not be affected as a result of taking part in this trial. It is important that you inform the trial team of any side effects as early as possible to ensure they do not affect your surgery should you not suffer serious conditions causing a delay.

Research biosamples
We will ask you to donate extra blood, urine and tissue samples for research related to this trial. These samples will be used to gain more detailed information on how your body and the cancer cells are affected by the trial drugs, and to give further details regarding the biology of the cancer.

The specific details of the research samples taken during this trial are given below.

Research Blood samples
The total amount of extra blood we will take from you for research related to this trial depends on the treatment schedule you are allocated to. We will take a maximum of 31mls of blood at each appointment (roughly 6 teaspoons). Of this 31mls, approximately 13mls will be used for your routine lab tests, and 18mls will be used for research purposes. These blood samples will be taken alongside routine bloods wherever possible. You may experience some discomfort when they are taken. These are timed as follows:

- During the screening period (Routine + Research - 31mls).
- On Day 1 (Routine ONLY - 13mls).
- On Day 15 (Routine + Research - 31mls).
- On the visit that takes place 3 days before surgery (Routine + Research - 31mls).
- At the End of Trial Follow-up visit, 3 months after surgery (Routine + Research - 31mls).

Research Urine samples
We will ask you to give extra urine for research related to this trial, and we may also use these samples to investigate the broader biology of the cancer. You will need to have fasted for these samples- your Research team will advise you regarding the requirements.

Research Tumour Tissue samples
During your nephrectomy, extra small samples of tumour and normal kidney tissue will be taken for research just prior to disconnecting the kidney from its blood supply. This procedure is extra and therefore would not normally be done. It will likely only add around 1 minute to your surgery. Each extra sample we take will be around the size of two long grains of rice put together lengthways, end-to-end. We will aim to take 6 samples. There is an additional risk of bleeding from this biopsy, which is predicted by the Investigators to be very small as your kidney will be removed moments later. This biopsy is important for the research: By looking at this sample (taken from this kidney whilst it still has blood flow), researchers can get the fullest picture of its activity following the drug treatment.

Following the nephrectomy, in the pathology department, the pathologist will take extra tissue from the kidney tumour and normal kidney tissue for research.

10. Are there any additional requirements?
If you agree to take part in this trial, there are a number of things that you must and must not do.

Hospital appointments, tests and assessments
You will need to attend all of the trial hospital appointments arranged by your trial team and agree to the tests needed for this trial.

Other medications
There are certain medications which you cannot take whilst you are receiving the trial drug(s) as the interaction between the medication and the trial drug(s) is not yet known. You must inform your trial doctor of any medications, over the counter medications, supplements, herbal remedies or alternative therapies you are taking or using. Your trial doctor will inform you if any changes are needed. You will also need to check with your trial team before taking any newly prescribed, over the counter medications, supplements, herbal remedies or therapies.

Other clinical trials
Whilst you are receiving the trial drugs you will not be able to participate in any other interventional research studies. It is possible that your participation in this trial may stop you being able to take part in some clinical trials in the future, depending on the nature of that trial.

Contraception, pregnancy and breastfeeding
Please also share this information with your partner if it is appropriate.

Trial medicines can harm an unborn baby or nursing infant. You will not be able to take part in this trial if you are pregnant or breastfeeding. You should also not participate in this trial if you are planning to become pregnant or father a child during the trial or for six (6) months after the completion of trial treatment.

Women of childbearing potential are required to use two highly effective forms of contraception for the duration of the trial (from signing the informed consent form until end of trial treatment) and for three (3) months after the completion of the trial treatment.

Acceptable non-hormonal birth control methods include:

- Intrauterine Device (IUD) PLUS male condom provided coils are copper-banded.
- True abstinence (where the participant refrains from any form of sexual intercourse and is in accordance with the participant's preferred and usual lifestyle).
- Vasectomised sexual partner PLUS male condom (with participant assurance that the partner has received post-vasectomy confirmation of azoospermia).
- Tubal occlusion PLUS male condom.

Acceptable hormonal birth control methods include:

- Hormonal shot or injection (e.g. Depo-Provera) PLUS male condom.
- Etonogestrel implants (e.g. Implanon, Norplant) PLUS male condom.
- Intrauterine system (IUS) device (e.g. levonorgestrel releasing IUS – Mirena) PLUS male condom.

Men and male partners are also required to use adequate contraception for the entire duration of the trial and for three (3) months after the completion of the trial treatment. This includes:

- Barrier contraception (condom and spermicide) even if female partner(s) are using another method of contraception or are already pregnant (also to protect male partners from exposure to the trial medicines).

- True abstinence (where this is in accordance with the participant's preferred and usual lifestyle).

Male participants should refrain from donating sperm for the duration of the trial and for three (3) months thereafter.

The risks of the trial drugs to the unborn child are currently unknown, so we would like to follow any pregnancies that occur to yourself or partner, during trial treatment or up to three (3) months after you complete trial treatment, up until the birth. This is so that we can learn more about the safety of the trial treatment.

If you or your partner becomes pregnant during the trial or within three (3) months of stopping treatment, you should inform your trial team immediately. Your trial team will discuss all the options available to you. The outcome and progress of any pregnancy will be followed by your trial team, (with your/your partner's consent), and we will ask you questions about the pregnancy and baby, if appropriate.

For Women of Child Bearing Potential: At the bottom of this document is an optional consent section. This is so that you can consent to the collection of data relating to your pregnancy, should a pregnancy occur.

For Male Participants: Should a pregnancy occur to your partner, your partner will be approached separately with information regarding the treatments you have received, and asked whether they would be willing to give consent for their pregnancy to be followed up.

Private insurance policies

You should discuss your participation in this trial with any insurance provider you have (e.g., protection insurance, life insurance, income protection, critical illness cover and private medical insurance) and seek advice if necessary, as failure to notify them may affect or invalidate your cover.

11. What are the side effects of the drug being tested?

Each person's reaction to a new drug is different. Some people have very few side-effects, while others may experience several. You will be closely monitored throughout the trial, but you should inform the trial team if you feel unwell or different in any way. If you have any major concerns or are feeling very unwell please contact your trial doctor immediately using the contact numbers at the end of this information sheet. Informing the trial team about any side-effects will help them manage your care, and they may be able to prescribe medication to ease any side effects. We also need to know so that the potential side-effects of this new combination can be recorded. A break from treatment may be necessary if you are experiencing side effects.

Side Effect	***Durvalumab***	**This table displays the side effects noted by the drug or drugs you will receive.**<br><br>**Very Common** side effects typically affect more than 1 in 10 people<br><br>**Common** side effects typically affect less than 1 in 10 people
Abdominal Pain	**Very Common**	
Blood Result Abnormalities	**Very Common**	
Bruising and/or pain from injection site	**Very Common**	
Cough	**Very Common**	
Decreased Appetite	**Very Common**	
Diarrhoea	**Very Common**	
Digestive problems	Common	
Dizziness and/or Sickness	**Very Common**	
Flu- like symptoms	Common	
High Temperature	**Very Common**	
Hoarse voice	Common	
Skin problems (itching, rashes)	**Very Common**	
Lung Infections	**Very Common**	
Kidney function changes	Common	
Mouth complications (dry or sore)	Common	
Night Sweats	Common	
Pancreatic Problems	Common	
Shortness of breath	**Very Common**	
Thyroid Function Changes	Common	
Tiredness and/or Weakness	**Very Common**	
Urinary Complications (pain or bleeding)	Common	
Water Retention (in limbs)	**Very Common**	

Uncommon side effects

In rarer instances (fewer than 1 in 100 people), other symptoms of taking these drugs have been noted that you should keep in mind before you agree to participate in the WIRE trial. Below these rare side effects are listed for the drug that you may be allocated, should you take part;

Durvalumab

- Long lasting autoimmune disease e.g.:
    - Immune related dermatitis (skin rash)
    - Thyroid problems
    - Underactivity of the adrenal glands (adrenal insufficiency)
    - Diabetes Mellitus Type 1 (where insulin is required)
    - Inflammation of the Pituitary Gland (hypophysitis)

Section 2: Conduct of the trial

12. What if new information becomes available?

Sometimes during the course of a trial, new information becomes available which might affect your decision to continue participating in this trial. Your trial team will contact you to discuss the new information and whether you wish to continue participating in the trial. If you still wish to continue on the trial, we will ask you to sign a new Informed Consent Form.

The trial sponsor, the regulatory authority or the trial doctor may decide to stop the trial at any time. If that happens we will tell you why the trial has been stopped and arrange for appropriate care and treatment for you.

13. What if I decide I no longer wish to participate in the trial?

You can stop participating in this trial at any time without giving a reason and without affecting your future care or medical treatment. If you decide not to participate any further, you will no longer receive the trial drugs. No further tests will be performed on you and no further research samples will be collected. Any data or results already collected will continue to be used in the trial analysis. Your trial team may also choose to withdraw you from the trial if they feel it is in your best interests or if you have been unable to comply with the requirements of the trial. Possible reasons for withdrawing you could include:

- Not taking the trial drugs as required
- Experiencing a serious side effect (your doctor will follow-up regarding your progress until this side effect has stabilised or resolved)
- Your disease gets worse whilst receiving trial treatment
- Finding out that you are not eligible after completing the screening tests
- Being unable to complete the visits or trial documentation as required
- Becoming pregnant or planning to become pregnant
- The trial doctor feels you no longer appear to benefit from the trial drugs.

14. What if there is a problem?

Any complaint about the way you have been dealt with during the trial or any possible harm you might suffer would of course be listened to and addressed wherever appropriate. If you have any concerns about any aspect of this trial you should speak to your trial doctor who will do their best to answer your questions.

In the event that something does go wrong and you are harmed by taking part in the research and this is due to someone's negligence then you may have grounds for a legal action for compensation against Cambridge University Hospitals NHS Foundation Trust or the University of Cambridge. The normal National Health Service complaints mechanisms will still be available to you (if appropriate). The University has obtained insurance which provides no-fault compensation, i.e., for non-negligent harm, you may be entitled to make a claim for this.

If you wish to complain or have any concerns about any aspect of the way you have been approached or treated during this trial, you can do this through the NHS complaints procedure. In the first instance it may be helpful to contact the (to be completed locally as appropriate – in England this will refer to the Patient Advice and Liaison Service (PALS)) at your hospital.

15. Will my taking part in this trial be kept confidential?

Cambridge University Hospitals NHS Foundation Trust (CUH) and The University of Cambridge are the Sponsors for this clinical trial based in the United Kingdom. They will be using information from you and your medical records in order to undertake this trial and will act as the data controller for this trial. This means that they are responsible for looking after your information and using it properly. The Sponsor organisations will keep identifiable information about you for 5 years after the trial has finished to ensure your safety and allow the trial to be reviewed by the authorities after it is finished.

Your rights to access, to change or to move your information are limited, as the Sponsor organisations need to manage your information in specific ways in order for the research to be reliable and accurate. To safeguard your rights, we will use the minimum personally-identifiable information possible.

You can find out more about how the Sponsor (s) use(s) your information using the information below:

- For Cambridge University Hospitals NHS Foundation Trust, please visit: https://www.cuh.nhs.uk/corporate-information/about-us/our-responsibilities/looking-after-your-information

Or email the Data Protection Officer at: gdpr.enquiries@addenbrookes.nhs.uk

- For University of Cambridge, please visit:
https://www.medschl.cam.ac.uk/research/information-governance
Or email the Information Governance team at: researchgovernance@medschl.cam.ac.uk

For participants recruited at CUH (where the Sponsor is also the site):

Cambridge University Hospitals will collect your name, NHS number and contact details to contact you about this trial and make sure that relevant information about the trial is recorded for your care and to oversee the quality of the trial. Individuals from the Sponsors and regulatory organisations may look at your medical and research records to check the accuracy of this trial. Cambridge University Hospitals will pass these details to the Sponsors along with the information collected from you and/or your medical records. The only people in the Sponsor organisations who will have access to information that identifies you will be people who need to contact you in relation to this trial and to audit the data collection process. Cambridge University Hospitals will keep identifiable information about you from this trial for *5* years after the trial has finished.

For participants recruited at other participating sites:

(Add site name) will keep your name, NHS number and contact details to contact you about this trial, and make sure that relevant information about the trial is recorded for your care, and to oversee the quality of the trial. Certain individuals from the Sponsors, and regulatory organisations may look at your medical and research records to check the accuracy of this trial. The Sponsor(s) will only receive information without any identifying information. **(Add site name)** will keep identifiable information about you from this trial for *XX* years after the trial has finished.

All information collected about you as a result of your participation in the trial will be kept strictly confidential. Your personal and medical information will be kept in a secured file and be treated in the strictest confidence.

Once you have agreed to participate in this trial you will be allocated a Trial ID Number. This is a unique trial number which will be used on all your trial documentation along with your date of birth. Your date of birth is considered to be personal information. We collect this personal information on trial documentation to help ensure that the data we receive as part of your trial participation is correctly allocated to you. By cross checking these two unique references we can ensure the integrity of the data.

When you agree to take part in this trial, the information about your health and care may be provided to researchers running other research studies in this organisation and in other organisations. These organisations may be universities, NHS organisations or companies involved in health and care research. Any serious side effects that occur during the trial will also be shared to the manufacturer of the trial drugs (AstraZeneca) using your trial-specific identifiers. The statistics for this trial is being undertaken by the University of Newcastle. All data that is shared with the University of Newcastle will be identified using only your trial ID and date of birth.

Your information will only be used for the purpose of health and care research, and cannot be used to contact you or to affect your care. It will not be used to make decisions about future services available to you, such as insurance.

Your coded trial data may be sent to other country(ies) outside the European Economic Area (EEA) for analyses, where the data protection laws are not the same. Your coded personal data may also be shared with off-site licenced laboratories for the purpose of analysing samples collected from you in this trial. However this information will not identify you and will not be combined with other information in a way that could identify you. The people who analyse the information will not be able to identify you and will not be able to find out your name, NHS number or contact details. Only anonymous trial data, without any personal information will be published at the end of the trial.
We will need to inform your GP of your participation in this trial. This is so that any medical decisions that your GP makes will take into account the fact that you are receiving drugs as part of this trial.

16. What will happen to my samples and scan images?

With your permission, we will analyse the blood, urine, kidney and tumour samples collected from you during this trial. This analysis will take place over a long period of time, so any results obtained will not alter your care pathway.

If you decide to stop taking part in the trial please tell the trial doctor if you want to change your mind about us using your donated sample(s) to look at the molecular and genetic makeup of the cancer.

<u>Routine blood samples</u>
Blood samples taken will be analysed immediately by your hospital and will be destroyed once the laboratory tests are complete. The results will include those you would receive as part of your standard care pathway, plus any results required for the trial. These results will be accessible to your trial team.

<u>Research samples</u>
Research samples taken will be labelled with your Trial ID Number, processed and stored initially by your trial team at your hospital, before being transferred to an approved facility for analyses. The approved facilities are either part of the Cambridge Biomedical Campus or

AstraZeneca These labs may be inside or outside of the UK. The samples will be securely stored and analysed by specialist teams. You will not be identifiable to researchers from any of the laboratories we may use for research analysis.

These samples will be analysed in a way which will tell us whether WIRE trial treatments are eliciting the biological response that we hope and expect to see. We will be for instance, assessing the tumour for a specific type of cell (CD8 positive T cells) that is indicative of your body fighting the cancer, which we believe should be more prevalent should you receive the Durvalumab drug. There may be other tests that we perform on your research samples to give us additional information regarding the drug activity.

Once the research analysis is complete any remaining samples will either be:

- Stored by the research team in line with the relevant regulatory requirements, in an appropriate facility pending ethical approval for use in a future trial outside of the WIRE trial.
- Will be disposed of in accordance with the UK Human Tissue Authority code of practice.

Archival tumour

You may have previously had a biopsy of your kidney, and therefore have more than one tumour sample. Not all patients will have undergone a previous biopsy. If you have however, we will ask your permission to access this 'Archival' tissue from your local hospital, so that we can compare the changes with your recent biopsy sample. This will also help us in the event that your most recent sample isn't big enough for us to perform our analysis. Granting us permission to access these samples is entirely optional.

Genetic Testing (All samples)

All of our cells in our body get information on how to work and function through a molecule called deoxyribonucleic acid (DNA). DNA is a record of instructions where the instructions are called genes. Genetic testing looks at your genes and could either look at one or some specific genes or all of your genes – your whole DNA. Genes affect how we grow and develop. Nobody else has exactly the same genes as you do, unless you have an identical twin. These differences mean that some people are more likely than others to get certain diseases and they also mean that medicines affect people differently. Some genes can be important in more than one disease.

Genetic testing in this trial is only for research purposes, but is an essential part of the trial. If you do not want to agree it to, you will be unable to take part. These genetic tests will be performed on any samples you donate to the trial, and requires no additional time from you.

The trial team may also undertake laboratory tests using your samples that reenact the environment of natural body conditions in a laboratory setting - so called '*ex vivo* modelling'.

This method of analysis typically provides very meaningful data regarding how cells interact naturally with the drugs received. Tissue cells are grown in a laboratory environment and could be modified to implant in mice with low resistance to disease. The laboratory staff will monitor the growth of the tumour (derived from your own) within the mice. Any laboratory performing this analysis would have the appropriate license for testing on mice. You have the right to take part in the trial without these particular lab procedures taking place. If you give your optional consent for these procedures, you may withdraw your consent for your samples to be used in this way at any point until this laboratory analysis begins.

Cambridge University Hospitals NHS Foundation Trust & the University of Cambridge may do this testing in its own laboratories or it may be done in the laboratories of other organisations (such as universities, collaborators and genetic specialist), or both. Results from this research may be shared and used as described in 'Will my taking part in this trial remain confidential?' In addition Cambridge University Hospitals NHS Foundation Trust & the University of Cambridge, and its designated organisations may conduct future research where they will share summary results (not your individual results) or anonymous datasets with other researchers, from other companies or universities, for example. The results may be combined with the results of other studies, for example as part of scientific databases. The researchers can only use this information for medical and healthcare related research.

Radiological (Scan) Images

The images obtained by way of CT and/or MRI scans will be coded, using only your trial ID number and Date of Birth. These coded images will then be sent on a secure CD, through the post, to the Sponsors (Cambridge University Hospitals and the University of Cambridge). These images will be stored on password-protected computers at Cambridge University for the duration of the trial.

CT scans will be used by your doctor to monitor general size changes to the tumour, and will likely be kept at your local hospital, but the central trial team may request them for checking of the measurements provided by your hospital. The people performing the analysis on your MRI images will not be able to identify you. They will be experienced Radiologists that will be able to identify areas of the cancer to get a general idea of how the cancer is behaving; so called 'Regions of Interest'. By using specialist software, the Radiologists can measure the amount of blood flowing through the cancer. This analysis will be part of the assessment of your MRI scan performed before your treatment, and after your treatment for comparison.

The anonymised images may support future research projects, for example finding new ways of gaining even more insight into renal cancer. Therefore, we ask for your permission to use these images, which do not identify you personally, in other future research projects.

17. What will happen to the results of the trial?

The results of the trial will be anonymous and you will not be able to be identified from any of the data produced. When the results of this trial are available they may be published in peer reviewed medical journals and used for medical presentations and conferences. They will also be published on the EU Clinical Trials Register website, a central registry for all clinical trials conducted in the EU.

Anonymised datasets from the trial will be made available to the funder (AstraZeneca), and may also be made available to other researchers in line with national and international data transparency initiatives.

If you would like to obtain a copy of the published results please contact your trial doctor directly who will be able to arrange this for you.

18. Who is funding the trial?

The trial is being funded by AstraZeneca and the Cancer Research UK Cambridge Centre. AstraZeneca is also supporting this trial by providing the trial drugs free of charge.

The trial is being managed by the Cambridge Clinical Trials Unit – Cancer Theme. The Chief Investigator is Mr Grant Stewart who is a University Lecturer at the University of Cambridge and Honorary Consultant Urological Surgeon at CUH.

19. Who has reviewed this trial?

All research within the NHS is reviewed by an independent group of people called a Research Ethics Committee, to protect your interests. This trial has been reviewed and given favourable opinion by the (name of REC here). The Medicines and Healthcare Products Regulatory Agency (MHRA) who are responsible for regulating medicines in the UK have also reviewed this trial.

20. Further information and contact details

If you have any questions concerning this trial please contact your:

Trial Doctor
Name: [INSERT your hospital contact details here]
Tel: [INSERT your hospital contact details here]
Research Nurse
Name: [INSERT your hospital contact details here]
Tel: [INSERT your hospital contact details here]

In the event of an emergency please contact:

24-hour contact details
Contact: [INSERT your hospital contact details here]
Tel: [INSERT your hospital contact details here]

Outside of an emergency,
You may wish to contact one of the following organisations that are independent of the hospital at which you are being treated:

CancerHelp UK is a registered charity providing information about all aspects of cancer. It can provide useful information on cancer treatments and medical research. You can contact their nurses on freephone 0808 800 4040. You can also access their web site at: www.cancerresearchuk.org

MACMILLAN Cancer Support is a registered charity providing information about all aspects of cancer. They have published several useful booklets on different types of cancer, chemotherapy, radiotherapy, and clinical trials in general. You can contact the nurses on freephone 0808 808 0000. You can also access their website at www.macmillan.org.uk

If you wish to complain or have any concerns about any aspect of the way you have been approached or treated during this trial, you can do this through the NHS complaints procedure. In the first instance it may be helpful to contact the Patient Advice and Liaison Service (PALS) at your hospital.

Patient Advice and Liaison Service (PALS) at:
Hospital Name: [INSERT your hospital PALS contact details here]
Tel: [INSERT your hospital PALS contact details here]

TO BE PRINTED ON HEADED PAPER

UNIVERSITY OF CAMBRIDGE

Cambridge University Hospitals NHS
NHS Foundation Trust

INFORMED CONSENT FORM

Trial Title: WIRE: WIndow-of-opportunity clinical trials platform for evaluation of novel treatments strategies in **RE**nal cell cancer.

Principal Investigator: [INSERT your PI name here]

Participant Number: ___________ (please add once the participant is registered)

**If you agree with each sentence below, please initial the box**		**INITIALS**
1	I have read and understood the Group 4, Durvalumab only, Participant Information Sheet version 3.0, dated 10th February 2020 for the above trial and I confirm that the trial procedures and information have been explained to me. I have had the opportunity to ask questions and I am satisfied with the answers and explanations provided.	
2	I understand that my participation in this trial is voluntary and that I am free to withdraw at any time, without giving a reason and without my medical care or legal rights being affected. I also understand that data or samples already collected will continue to be used in the trial analysis.	
3	I understand that personal information about me will be collected and used in accordance with this information sheet. This information will be kept in the strictest confidence and none of my personal data will be published.	
4	I understand that sections of my medical notes or information related directly to my participation in this trial may be looked at by responsible individuals from the sponsor, regulatory authorities, research personnel where it is relevant to my taking part in research and that they will keep my personal information confidential. I give permission for these individuals to have access to my records.	
5	I understand that my GP will be informed of my participation in this trial and sent details of the WIRE trial.	
6	I have read and understood the compensation arrangements for this trial as specified in the Participant Information Sheet.	
7	I understand that the team/doctors in charge of this trial may close the trial or stop my participation in it at any time without my consent.	
8	I have read and understood my responsibilities for the trial including using appropriate contraception as listed in section 6.	
9	I agree to give blood and urine for research related to this trial.	
10	I agree to give tumour and healthy tissue samples for research related to this trial	
11	I give my permission to allow my kidney tissue and research blood, urine & tumour samples to be sent to specialist teams on the Cambridge Biomedical Campus, or to laboratories contracted and approved by the Sponsor(s) for tests/analysis related to this trial. I understand that only the minimum information will be supplied and that I will not be identifiable to any of the researchers.	
12	I understand that the results from the analysis carried out on the research blood and tumour samples will not be fed back to me.	
13	I understand that the information collected about me will be used to support other research in the future, and may be shared anonymously with other academic and commercial researchers external to the project, within the UK and beyond	

14	I give my permission to allow for DNA testing of my samples	

		INITIALS	
**OPTIONAL:** Please read each statement below and initial the relevant box		Yes	No
15	I give my permission to allow for any archival tumour tissue to be collected for tests related to this trial. I understand that only the minimum information will be supplied and that I will not be identifiable to any of the researchers.		
16	I give my consent for my tissue samples to be used to grow cell cultures or be implanted into mice		
17	I give my permission to allow any remaining research blood and tumour samples taken as part of this trial to be retained in an HTA licenced facility pending Ethical Approval for use in another project at the discretion of the Chief Investigator. I understand that these samples will be retained in an approved storage facility, that only the minimum information will be supplied and, that I will not be identifiable.		
18	I give my permission for my anonymised radiological images to be used as part of the data for future research studies		

		INITIALS	
**FOR WOMEN OF CHILDBEARING POTENTIAL ONLY**<br>**OPTIONAL:** Please read each statement below and initial the relevant box		Yes	No
19	If I become pregnant during, or in the three (3) months after receiving the trial drugs, I agree to information being collected about me, my pregnancy and my baby.		
20	I understand that sections of my medical notes or information related directly to my pregnancy may be looked at by responsible individuals from the sponsor, regulatory authorities and research personnel where it is relevant to my taking part in research. I give permission for these individuals to have access to my records.		
21	I agree to give my pregnancy information voluntarily and understand that I am free to withdraw at any time without giving a reason and without my medical care or legal rights being affected. I understand that all data collected up to the withdrawal of consent will be kept confidential.		

I agree to participate in this trial:

______________________ ______________________ ______________________

Name of participant **Signature** **Date**

______________________ ______________________ ______________________

Name of person taking consent **Signature** **Date**

Time of consent (24hr clock): _______:_______

1 copy for the participant, 1 copy for the trial team, 1 copy to be retained for hospital records

CANCER RESEARCH UK | CAMBRIDGE CENTRE

UNIVERSITY OF CAMBRIDGE Cambridge University Hospitals NHS NHS Foundation Trust

CCTU Cambridge Clinical Trials Unit

TO BE PRINTED ON HEADED PAPER

UNIVERSITY OF CAMBRIDGE

Cambridge University Hospitals NHS
NHS Foundation Trust

WIRE
NOVEL TREATMENTS IN RENAL CELL CANCER

Group 5 - Olaparib & Durvalumab Combination

PARTICIPANT INFORMATION SHEET & INFORMED CONSENT FORM

WIRE: WIndow-of-opportunity clinical trials platform for evaluation of novel treatment strategies in **RE**nal cell cancer.

You are being invited to take part in a research trial. Before deciding whether to take part, you need to understand why this research is being done and what it involves.

Please take time to read the following information carefully and talk to others about the trial if you wish. Please ask your trial team if anything is not clear or if you would like more information. Please take time to decide whether or not you wish to take part. You will be receiving this Participant Information Sheet before any diagnosis has been made i.e. it is not yet confirmed that you have renal cell cancer.

Section 1 tells you the purpose of this trial and how the trial would involve you, including a description of what the various tests are, and some of the risks and restrictions of taking part

Section 2 gives you more detailed information about the trial process, including information about how the trial is run, and how we may use your data.

Section 1: Purpose of the trial and what will happen

1. What is the purpose of the trial?

Kidney cancer is the 7th most common cancer in the UK. Currently many patients with this cancer are offered a kidney removal operation (known as nephrectomy), which cures many patients with no further need for treatment. However, some may receive drug treatment after surgery.

WIRE is a trial that will administer drugs to patients in the time between the decision to operate and the nephrectomy operation. Patients are not usually given anti-cancer drugs during this period, and indeed may never need them, but it could give researchers important information to guide the best combinations of anti-cancer drugs to prioritise for later phase clinical trials. The trial will investigate the effect and safety of 3 different cancer drugs, individually or in combination, in patients with a specific type kidney cancer called clear cell renal cell cancer. The cancer may be localised (within the kidney) or metastatic (it has spread outside of the kidney).

2. What are the drugs being tested?

There are 3 drugs being used in this trial, Cediranib, Olaparib and Durvalumab. All three of these drugs target different parts of cancerous cells which might decrease the size of the tumour over a period of time. However, the duration of treatment in this trial is very short in comparison to how long these drugs would normally be given for. Therefore, it is unlikely that the trial treatment will noticeably reduce the tumour size if it is confirmed that you do have kidney cancer. . In this trial, we will primarily be looking at cancer’s response to these drugs by taking a closer look at the tumour cells in the laboratory and by using new imaging technology.

Although the trial uses 3 drugs, we will only be giving you one or two of these drugs. The drugs you are given will depend on the group you are allocated. The trial team already know which group you will be allocated. Currently, we are recruiting participants for the **Olaparib and Durvalumab** group. Each group has its own specific entry criteria which may involve different tests and assessments; this is discussed further under *Section 4 Do I have to take part?*

Olaparib is also a cancer therapy drug called a ‘PARP Inhibitor’. PARP is a protein which helps damaged cells to repair themselves. “PARP Inhibitors” block the ability of damaged (cancer) cells to repair themselves. Olaparib is being tested in clinical trials, but is currently only licenced in the EU for use in patients with ovarian and breast cancers. This drug is an oral tablet (a tablet taken by mouth), which will be taken for at least 14 days in total, and until the morning of your surgery.

Durvalumab is an immunotherapy drug which enables a person’s immune system to work better against cancer cells. It is a type of targeted anti-cancer drug called a monoclonal antibody (mAb) which binds to certain cells or proteins. Durvalumab seeks cancer cells by looking for a particular protein and attaching to it. By doing this durvalumab may help the individual’s immune system attack the cancer and stop it from growing. Durvalumab is being tested in clinical trials, but is currently only licenced in the EU for use in patients with a type of lung cancer. This drug is given as a once-only, hour-long infusion in hospital via a cannula (a needle with a small plastic tube) placed into a vein in your hand or arm. This infusion will take place at least 14 days before your surgery.

3. Why have I been invited?

You have been invited to participate in this trial because you may have renal cell cancer (kidney cancer) that may not be cured with surgery alone. The diagnosis of renal cell cancer will be confirmed or disproved with a pre-surgery biopsy. In normal care, usually your treating doctor will recommend proceeding with a nephrectomy or partial nephrectomy (removal or partial removal of the kidney) without the need of a pre-surgery biopsy. However, as part of the WIRE trial, participants will be required to undergo a biopsy during the screening stage to determine whether they have the type of cancer we are investigating.
We plan to include up to 76 patients with kidney cancer from approximately 2 hospitals across the UK.

4. Do I have to take part?

Participating in this trial is completely voluntary. If you decide to participate you will be asked to sign an Informed Consent Form. You are however still free to change your mind and leave the trial at any time and without giving a reason. If you chose not to participate or to leave the trial, your current and future medical treatment will not be affected in any way. Your normal standard care will not be affected in any way.

You should also be aware that entry into this trial requires you to meet some strict entry criteria that your doctor will assess you for over the next few weeks, during a period called

'screening'. At the moment we will not know whether you are eligible for the trial as there are further tests and assessments to do, but some people who consent to take part in this trial may not meet the entry criteria to receive the trial drugs.

Before proceeding, it is important to understand that you may not benefit from the extra drugs and procedures involved in this trial. Surgery alone may be sufficient to cure the cancer alone, although there is no way to predict this for certain. The possible side effects could potentially delay your surgery, and participation in the trial could make you feel fatigued and nauseous. However, your doctors will be monitoring how you are feeling very closely, making every attempt to minimise any side effects.

It is also important you are aware that based on previous research the average chance of being cured by surgery alone needing no further treatments is 42%, in whom no further treatments would be needed. It is also important that you are aware that you will not have access to the trial drugs after your operation, and you might not be permitted to take part in other clinical trials if you decide to participate in the WIRE trial.

5. Expenses and payment

You will not receive any payment for participating in this trial however we can reimburse reasonable travel and parking costs incurred by your participation in this trial. This is up to a maximum of £180 for the entire duration of your participation in this trial. We will ask you to retain your receipts wherever possible.

6. What are the possible advantages and disadvantages of taking part?

It is unlikely that you will benefit clinically from taking part in this trial, although it is possible that the tumour may shrink very slightly. It is also hoped that taking the medications used in the trial could provide you with a better outcome, but this cannot be directly measured in this trial. You will gain access to drugs that are only available within a clinical trial. You are likely to have more contact and for longer with your care team than if you were not involved in a trial, and some patients gain reassurance from this. It is hoped that information collected as part of your participation in this trial will benefit patients with kidney cancer in the future and you may contribute to their future treatment and care. Your care team will be able to share information with you regarding how the tumour cells responded and/or any tumour size reduction, if you wish to know this.

In addition to the above benefits there are also aspects of your participation you may find difficult. For instance, if you decide to take part:

- You will need to undergo a biopsy at the screening stage which is likely to require a full day at the hospital. It is also likely that this biopsy would not have been required if you were not participating in this trial. This is an extra procedure for about 70% of patients. You can find more information about this under section 9 – Tumour biopsy on page 12.
- There will be an increased number of hospital visits due to participating in the trial. You will be required to come to the hospital an extra 4-5 times during the trial. These visits are to conduct initial assessments and scans (1 extra visit); clinic visits whilst receiving the trial drug(s) (2-3 extra visits); a visit for the scan before surgery (1 extra visit). The average length of time per visit is about 2 or 3 hours, but actual time may vary. The schedule and timing of these visits is available on page 9.
- You will undergo at least an additional 2 MRI scans if you decide to take part in this trial, with potentially another 2 MRI scans using a new type of scan called a

‘hyperpolarised MRI’ if available at your hospital (see section 9, pages 11 and 12 for more information). An additional 1-2 CT scans may be required as recommended by the doctor based on information from your diagnosis and previous scans. You would not have normally received these scans if you were not participating on the trial.

- You could experience drug side effects such as nausea, fatigue, diarrhoea, dizziness, decreased appetite and blood test abnormalities. A full list of known side effects is listed on pages 15 to 16. It is also possible that some of these side effects might delay your surgery.
- There will be 6 additional tissue biopsies (samples) taken during your surgery, this will likely only add a few minutes to your standard surgical procedure. Whilst these are unlikely to impact you or prolong your stay in hospital, it is important that you are aware these are taken. More information about these tissue samples is mentioned on page 13.

  The drugs you will be taking as part of this trial will not be given to you after the surgery, and may prevent you from taking part in other clinical trials after your surgery.

7. What are the alternatives for treatment?

The standard care for you is a nephrectomy (removal of the kidney) or partial nephrectomy (removal of the tumour only) without any drug treatment before surgery.

8. What happens when the trial stops?

Following completion of the trial you will not be able to receive any further trial drug treatment and we will refer you back to your primary consultant. You will still be able to contact your trial Nurse or Practitioner, however, with any questions about the trial.

9. What will happen to me if I take part?

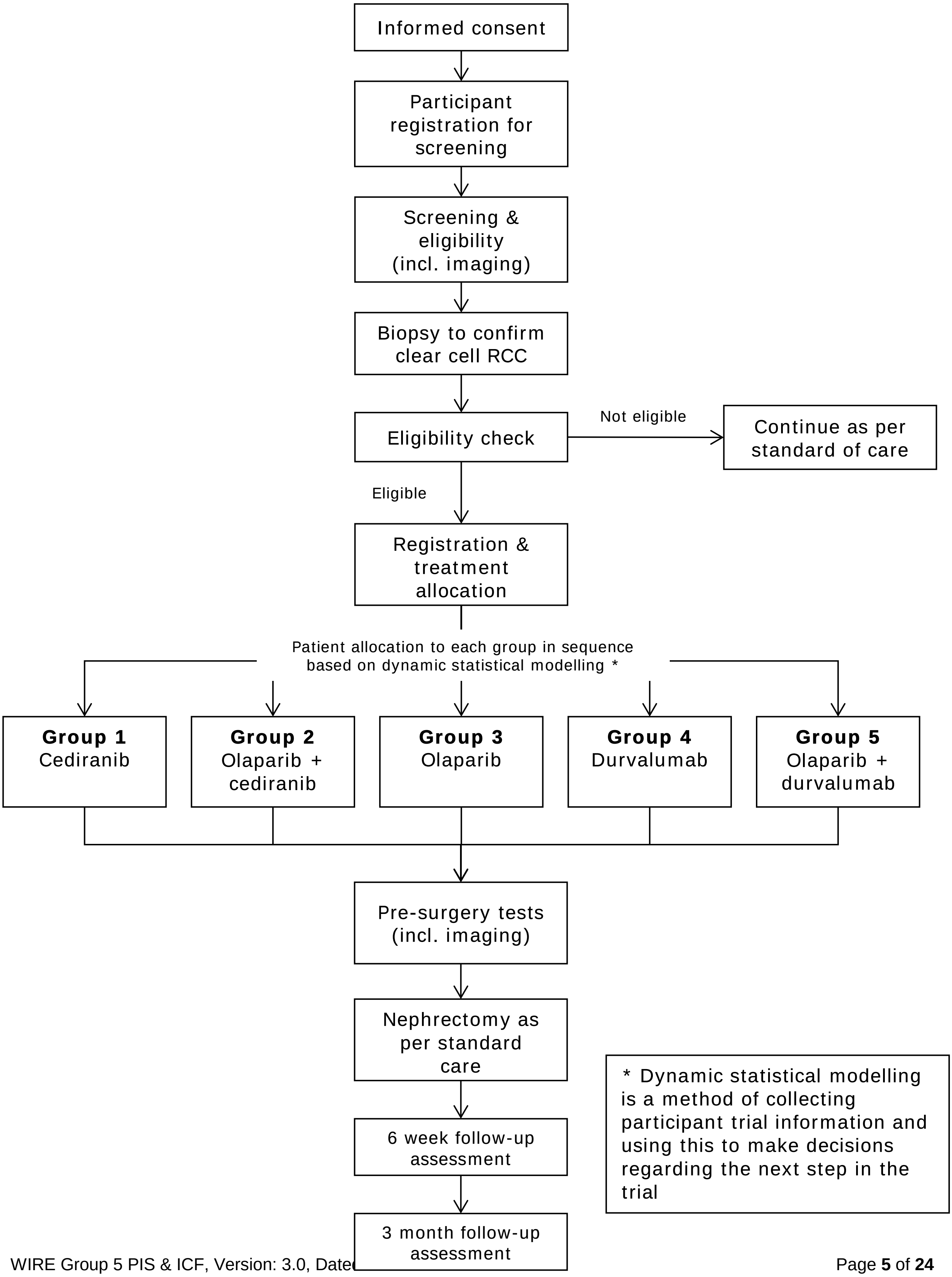

Hospital Appointments

Informed Consent

If you agree to participate in the trial, you will be invited to sign the Informed Consent Form at the end of this document. You will be given a copy of this to take away and refer to later.

Screening and baseline

Standard of care visit plus extra trial assessments 5-6 hours in duration. Screening may require an additional visit to complete all assessments, dependent on hospital capacity.

To check that you meet all of the requirements for entering this trial you will need to have some tests. This is called the "screening period". You will be given appointments to see the trial team for these tests, which will be carried out within a 28 day period. This may include more than one visit to the hospital. All of the tests listed during this period and the following visits are further explained later in this information sheet (see page 5). All of the tests listed under Screening will collect data for the research trial.

- A check of your weight, blood pressure and temperature
- A full physical examination
- Clinical review
- Performance status check
- Blood and urine sampling
- 3 Electrocardiogram assessments (ECGs), 5 minutes apart.
- A minimum of one type of scan, including a Dynamic Contrast Enhanced Magnetic Resonance Imaging (DCE-MRI) scan at least (a type of scan that uses strong magnetic fields and radio waves to produce detailed images of the body and organs). You may undergo a hyperpolarised MRI scan (a new MRI scanning technique which produces extremely detailed images of the body and organs), depending on the group you are allocated to, and the treatment centre you are attending. You may also undergo a CT scan, depending on how long ago your last CT scan took place (the risks of increased radiation exposure are explained further on in this document, in the 'Tests and Assessments' section).
- Collection of your Medical History
- Research blood and urine sampling- we will take these samples before you have the tumour biopsy, on the same day
- Tumour Biopsy
- Pregnancy test (female participants only)

Your weight, blood pressure, temperature, physical examination, performance status check, blood & urine samples, CT and ECGs may be assessed as part of your standard care pathway (these would usually take place without trial participation anyway as part of the usual pre-surgery checks). A tumour biopsy would not normally take place in about 70% of patients, but all participants will have a biopsy in order to be eligible for the trial.

Additional ECGs, a possible repeat CT scan (if you haven't had one within the last 28 days) and all of the other assessments and scans listed are extra for the trial.

Registration

Some of the tests performed during Screening require the results to fall within certain trial ranges. If any of your results do not fall within these ranges, you will not be able to participate in this trial and will be referred to follow the usual treatment pathway for patients with your condition. However, if the screening tests show that you are suitable to participate in this trial, your clinical team will register you into the trial.

There are 5 different treatment groups in this trial, but each participant will only be allocated to one of these groups, and given the appropriate information sheet for the drug that they might receive. If you decide to participate and meet the entry criteria, you will be allocated to Group 5 and be given Durvalumab & Olaparib.

Clinic Visits

Extra visits for the trial, up to 3 hours per visit in duration

Once your participation in this trial has been confirmed we will ask you to attend regular clinic appointments to see the trial team whilst you are receiving the trial drug/s and we will perform a number of trial related tests to ensure you are tolerating the trial drugs well. Initially these will be performed on the 1st and approximately 15th day of your treatment but will be flexible around weekends and bank holidays. You will need to have fasted for 6 hours before the Research urine tests, and your research/care team will advise you regarding the timescales on this. Both of these visits are extra visits for the trial and all assessments for these visits below are needed just for the trial and are outside of standard care:

- A weight, blood pressure and temperature check
- A physical examination (if your doctor believes it is necessary)
- Performance status check
- Clinical review
- At least 1 electrocardiogram assessment (ECG). Based on your results, your trial team may ask you to perform 2 additional ECGs, all 3 taken 5 minutes apart.
- Blood and urine assessments. Pre-treatment (Day 1)
- Pregnancy test (female participants only)
- Dispensing of your tablets to take home, and a diary to record when you take them (at Day 1 only).
- You will be administered your Durvalumab on your Day 1 visit.

In addition to the assessments above, the following tests will be performed on Day 15 ONLY

- Research blood and urine sampling
- Assessment of any side effects from the medication
- Check that you have enough and are correctly taking, your medication

Your clinical trials team may decide to give you an additional assessment on approximately the 22nd day of treatment, if you are still taking the trial drug. This may be by telephone or in clinic; this day 22 assessment will be an extra assessment for the trial, and the below tests will be extra assessments for the trial, outside of standard of care:

- Performance status check
- Assessment of any side effects from the medication
- Check that you have enough and are correctly taking, your medication
- Clinical review

If you experience unmanageable side effects, or if your trial doctor thinks you should not continue, the trial drugs will be stopped and you will return to the normal standard of care provided by the hospital for the treatment of the cancer.

Pre-surgery

Extra visit, up to 6 hours in duration

Within 3 days of your surgery date, you will undergo at least one scan to assess whether the medication you have been taking is affecting the tumour. These are all extra assessments

for the trial, outside of standard care, and the visit could take most of a day or more than one day:

- DCE-MRI
- Hyperpolarised MRI (depending on the group you are allocated to, and the treatment centre you are attending)
- CT scan (if the cancer has spread outside the kidney- your Trial Nurse or Practitioner will advise you)
- Research blood and urine sampling (these are outside of standard care and are required for the trial only)
- Routine blood sampling

Surgery

Standard of care visit. Surgical prep plus 3 hours surgery time.

During your nephrectomy, we will obtain tumour samples, as well as samples of the normal kidney tissue next to the tumour. The surgery is standard care but the amount of samples taken will be higher than in standard care.

Follow-up – 6 weeks after surgery

Standard of care visit, up to 2 hours in duration

You will be given an appointment to see the trial team approximately 6 weeks after surgery to ensure that you have recovered from any side effects. This 6 week check is usual for patients who have undergone nephrectomy, and is standard care. During this visit you will have:

- Performance status check
- Clinical review
- Routine Blood sampling
- Pregnancy Test

End of trial follow-up – 3 months after surgery

Standard of care visit, up to 2 hours in duration

You will be given another appointment to see the trial team approximately 3 months after surgery to ensure that you have tolerated the trial drugs well. A CT scan will be performed prior to this visit. Following this visit, you will not need to attend any further trial related visits. This 3 month check is usual for patients who have undergone nephrectomy, and is standard care. During this visit you will have:

- Performance status check
- Clinical review
- Routine blood sampling
- Research blood and urine sampling (these are outside of standard care and are required for the trial only)
- Pregnancy test

What will happen and when?

Tests and Assessments	Screening (up to 28 days prior to Day 1 of treatment)	Treatment phase			Pre-surgery	Surgery	Follow-up	
		**Day** 1 (± 5 days)	**Day 15** (± 5 days)	**Day 22 (Optional)** ± 5 days			6 weeks after surgery ± 7 days	3 months after surgery ± 14 days
Consent	X							
Medical history	X							
Vital signs	X	X	X					
Performance status check	X	X	X	X			X	X
Clinical review	X	X	X	X			X	X
Physical examination	X	If clinically necessary	If clinically necessary					
ECG	X	X	X					
Urine sample	X	X	X				X	X
Blood for routine tests	X	X	X		X		X	X
Pregnancy test	X	X	X					X
Post-menopausal test	X							
Blood for research tests	X		X		X			X
Urine for research tests	X		X		X			X
DCE-MRI	X				X			
Hyperpolarised MRI	If needed				If needed			
CT scan (*your doctor will tell you if needed)	X*				If needed*			X
Tumour biopsy (screening)	X							
Durvalumab Administration		X						
Olaparib Drug dispensing		X						
Olaparib Drug compliance check			X	X				
Tissue samples (research)						X		
Full or partial nephrectomy						X		
Total time of visit	5-6 hours over 2-3 visits	2-3 hours over 1 visit	2-3 hours over 1 visit	1-2 hours over 1 visit or 20 min phone call	4-6 hours over 1-2 visits	2-3 hours of surgery	1-2 hours over 1 visit	1-2 hours over 1 visit

Tests & Assessments

You will be monitored carefully by the trial team whilst you are on this trial. Most of the tests in this trial are necessary to give you the trial drugs safely and ensure you are tolerating the trial drugs well. These tests are explained below, including the possible **risks and/or benefits** of each.

Medical history
Your medical history will be reviewed by the trial team during the screening period. This will involve a discussion about your past and present health, lifestyle and any symptoms you may currently have. This is so that the trial team are aware of any pre-existing conditions. The trial team will also record your gender, ethnicity and date of birth. This is to collect demographic data for the trial.

Weight, blood pressure and temperature
The trial team will monitor your blood pressure and temperature in order to properly assess the side effects to your treatment. These indicators will help the doctors to properly diagnose you and to manage your care.

Clinical review
The trial team will regularly discuss and review your general wellbeing, discuss any symptoms and/or side-effects that you may have, and record any medications you may be taking. This is so that the trial team has a full picture regarding your current state of health.

Your trial team may end your participation in this trial if you have severe or unmanageable side-effects to the trial drug/s. If this happens your trial team will follow-up with you regarding your progress until the side effect has stabilised or resolved.

Physical examination
Your trial doctor will perform a physical examination during the screening period and whilst you are receiving the trial drugs. This will ensure that the trial team has a full picture regarding your current state of health.

Drug Compliance Check
It is important that you take the trial drugs as directed by your trial team. We will provide you with a diary to log the medication that you are taking as part of this trial (olaparib). You must also return any tablets or the empty container to the trial team at the end of your treatment and bring your diary with you to each visit.

Electrocardiogram (ECG)
You will be asked to have several ECGs as part of this trial during the screening period and whilst you are receiving the trial drugs. These record an electrical trace of your heart rhythm and are required to confirm that you are fit enough to receive the trial drugs. They will also monitor the health of your heart during the trial. We will ask you to lie down whilst a series of leads are placed on your chest area, wrists and ankles with adhesive pads, these will record a trace of your heart rhythm. This takes a few minutes and is painless. This is to check the health of your heart.

Performance Status check
Your performance status check will track the impact that your condition or treatment has on your day-to-day activities and on your “usual” level of day-to-day functioning. This is assessed by asking you questions about your level of activity around the house or at work (as applicable).

Pregnancy test/ Non-childbearing status (female participants only)
If you are female and of childbearing potential you will be asked to give a blood sample during the screening period **to confirm that you are not pregnant before you can be enrolled into this trial.** You will also have several pregnancy tests during the trial. If you are of non-childbearing potential you may be asked to provide a sample of blood during the screening period to confirm your non-childbearing status. This is because we do not yet know the effect of the trial drugs on a developing baby (foetus). This is an extra sample but can be taken from the same bottle as the other blood tests. Most female patients would normally have a urine test.

MRI Scans
You will undergo two DCE-MRI scans if you participate in this trial, and may also have two hyperpolarised MRI scans. It is important that you are aware that the MRI scans would not have been required if you were not participating on this trial. You may need an injection of a contrast agent to improve the quality of the scan. The injection is usually given in a vein in your elbow (the same place as a blood test), which may cause discomfort and/or bruising. When you have the scan, you will be asked to lie still on a couch which will slide into a metal tunnel, which some people find this uncomfortable and/or claustrophobic. The scan lasts for about 45-60 minutes. You will also be able to talk to the radiographer whilst you are having your scan, or listen to music if you prefer. If you are anxious in enclosed spaces you may be prescribed a tablet to help you relax before you have the scan.

DCE-MRI scan
We will ask you to have a DCE-MRI scan during the screening period and before your surgery. DCE-MRI is a type of scan that uses strong magnetic fields and radio waves to produce detailed images of the body and organs. We do this to establish how the trial drugs affect blood flow in the tumour and how the cancer is responding to the trial drugs.
It is known that small amounts of these contrast agents may remain in the brain after a scan with these agents, although there is currently no evidence that these small amounts cause any harm and these agents have been used for many decades. These contrast agents are routinely given during a kidney cancer MRI so we can have a clear understanding of the blood supply to the cancer. You may experience some side effects such as nausea, headaches and dizziness which are very common side effects. Other side effects such as vomiting, injection site reaction, laboured breathing, and allergic reactions are less common. You should tell a staff member immediately if you experience any of these symptoms so you can be treated promptly. We will use the lowest dose of the contrast agent required for a clear image and if you have any questions about your scan, please speak to your doctor.

Hyperpolarised MRI
You may also be asked to have hyperpolarised MRI scans during the screening period and before your surgery. These scans will enable researchers to assess the cancer tumours and the effectiveness of trial drugs. Not every participant will receive this scan, depending on the numbers already in the trial and local availability.
The contrast agent to be injected is a breakdown product of sugar called pyruvate. Although hyperpolarized carbon MRI is a new technology, tests to date have demonstrated no significant safety issues. Pyruvate is a naturally-occurring molecule in the body. Rarely, people who have had an injection of pyruvate in previous studies developed: unusual taste within the mouth, headaches, flushing, diarrhoea or dizziness after the injection. These side effects were mild and short lasting. Although

it is unlikely that an allergic reaction or other side effect will occur, there are facilities in place within the MRI unit, and within the hospital, to manage these appropriately. If you have any questions about your scan, please speak to your doctor.

Computerised Tomography (CT) scan

You may have a CT scan during the screening period (depending on how recent your last one was) and, if required, approximately 3 days before your surgery (you will be told in advance if this includes you). You will also have one just before your 3 month follow up visit. CT scans are used to take pictures of, measure and assess cancer tumours.

When you have the scan, we will ask you to lie still on a couch which will slide through the scanning machine. The scan lasts about 30 minutes, sometimes longer. You may need to drink or have an injection of a contrast agent into a vein which improves the quality of the scan images. You may however be observed for some time after the CT scan to check for a reaction to the contrast agent, which is very rare. Some common side effects that you may experience is feeling hot. Some uncommon side effects include feeling sick, increased sweating, feeling cold, and dizziness. Allergic reaction symptoms can include mild itching or hives (small bumps on the skin) to shortness of breath and swelling of the throat or other parts of the body. You should tell a staff member immediately if you experience any of these symptoms so you can be treated promptly

Some CT scans are part of standard care and you would have had these anyway, whether you take part in the trial or not. Participation in this trial may mean that you receive 1-3 additional CT scans compared with normal care, depending on your result. These procedures use ionising radiation to form images of your body. Ionising radiation can cause cell damage that may, after many years or decades, turn cancerous. The chances of this happening to you as a consequence of taking part in this trial are approximately 0.3 %. If you are concerned about the level of radiation you are having or have had please talk to your trial team.

Tumour biopsy

You will have a tumour biopsy (removal of a sample of tumour tissue with a special needle) during the screening period. The sample will be around the size of two long grains of rice used to diagnose that you have the type of kidney cancer (clear cell) which may confirm that you are eligible for WIRE. The biopsy will be performed in hospital. A type of imaging (e.g. ultrasound) may be used to help guide the needle and reduce the risk of complications. You will receive a sedative to make you comfortable throughout the procedure. The total duration of the tumour biopsy is 30 minutes, plus an observation period of 6 hours. This period is to manage any side effects appropriately. There may be pain, discomfort and/or bleeding as a result of a biopsy.

This is a research procedure for most participants, performed after your DCE-MRI scan.

Routine Blood and Urine sampling

We will ask you to give blood and urine samples for safety purposes throughout the trial to ensure that you are well enough to receive the trial drugs, and to monitor for any trial drugs side effects that you may be suffering from. Please see '*Research biosamples*' for a description of the amount of blood and urine that we are taking.

Kidney Surgery (Nephrectomy)

The nephrectomy (surgical removal of a kidney) or partial nephrectomy (removal of the tumour from the kidney) will be performed by your hospital. The kidney surgery is standard

of care and should not be affected as a result of taking part in this trial. It is important that you inform the trial team of any side effects as early as possible to ensure they do not affect your surgery should you not suffer serious conditions causing a delay.

Research biosamples

We will ask you to donate extra blood, urine and tissue samples for research related to this trial. These samples will be used to gain more detailed information on how your body and the cancer cells are affected by the trial drugs, and to give further details regarding the biology of the cancer.

The specific details of the research samples taken during this trial are given below.

Research Blood samples

The total amount of extra blood we will take from you for research related to this trial depends on the treatment schedule you are allocated to. We will take a maximum of 31mls of blood at each appointment (roughly 6 teaspoons). Of this 31mls, approximately 13mls will be used for your routine lab tests, and 18mls will be used for research purposes. These blood samples will be taken alongside routine bloods wherever possible. You may experience some discomfort when they are taken. These are timed as follows:

- During the screening period (Routine + Research - 31mls).
- On Day 1 (Routine ONLY - 13mls).
- On Day 15 (Routine + Research - 31mls).
- On the visit that takes place 3 days before surgery (Routine + Research - 31mls).
- At the End of Trial Follow-up visit, 3 months after surgery (Routine + Research - 31mls).

Research Urine samples

We will ask you to give extra urine for research related to this trial, and we may also use these samples to investigate the broader biology of the cancer. You will need to have fasted for these samples- your Research team will advise you regarding the requirements.

Research Tumour Tissue samples

During your nephrectomy, extra small samples of tumour and normal kidney tissue will be taken for research just prior to disconnecting the kidney from its blood supply. This procedure is extra and therefore would not normally be done. It will likely only add around 1 minute to your surgery. Each extra sample we take will be around the size of two long grains of rice put together lengthways, end-to-end. We will aim to take 6 samples. There is an additional risk of bleeding from this biopsy, which is predicted by the Investigators to be very small as your kidney will be removed moments later. This biopsy is important for the research: By looking at this sample (taken from this kidney whilst it still has blood flow), researchers can get the fullest picture of its activity following the drug treatment.

Following the nephrectomy, in the pathology department, the pathologist will take extra tissue from the kidney tumour and normal kidney tissue for research.

10. Are there any additional requirements?

If you agree to take part in this trial, there are a number of things that you must and must not do.

Hospital appointments, tests and assessments
You will need to attend all of the trial hospital appointments arranged by your trial team and agree to the tests needed for this trial.

Other medications
There are certain medications which you cannot take whilst you are receiving the trial drug(s) as the interaction between the medication and the trial drug(s) is not yet known. You must inform your trial doctor of any medications, over the counter medications, supplements, herbal remedies or alternative therapies you are taking or using. Your trial doctor will inform you if any changes are needed. You will also need to check with your trial team before taking any newly prescribed, over the counter medications, supplements, herbal remedies or therapies.

Other clinical trials
Whilst you are receiving the trial drugs you will not be able to participate in any other interventional research studies. It is possible that your participation in this trial may stop you being able to take part in some clinical trials in the future, depending on the nature of that trial.

Dietary restrictions
It is prohibited to consume grapefruit juice or Seville oranges if you are taking olaparib.

Contraception, pregnancy and breastfeeding
Please also share this information with your partner if it is appropriate.

Trial medicines can harm an unborn baby or nursing infant. You will not be able to take part in this trial if you are pregnant or breastfeeding. You should also not participate in this trial if you are planning to become pregnant or father a child during the trial or for six (6) months after the completion of trial treatment.

Women of childbearing potential are required to use two highly effective forms of contraception for the duration of the trial (from signing the informed consent form until end of trial treatment) and for three (3) months after the completion of the trial treatment.

Acceptable non-hormonal birth control methods include:

- Intrauterine Device (IUD) PLUS male condom provided coils are copper-banded.
- True abstinence (where the participant refrains from any form of sexual intercourse and is in accordance with the participant's preferred and usual lifestyle).
- Vasectomised sexual partner PLUS male condom (with participant assurance that the partner has received post-vasectomy confirmation of azoospermia).
- Tubal occlusion PLUS male condom.

Acceptable hormonal birth control methods include:

- Hormonal shot or injection (e.g. Depo-Provera) PLUS male condom.
- Etonogestrel implants (e.g. Implanon, Norplant) PLUS male condom.
- Intrauterine system (IUS) device (e.g. levonorgestrel releasing IUS – Mirena) PLUS male condom.

Men and male partners are also required to use adequate contraception for the entire duration of the trial and for three (3) months after the completion of the trial treatment. This includes:

- Barrier contraception (condom and spermicide) even if female partner(s) are using another method of contraception or are already pregnant (also to protect male partners from exposure to the trial medicines).
- True abstinence (where this is in accordance with the participant's preferred and usual lifestyle).

Male participants should refrain from donating sperm for the duration of the trial and for three (3) months thereafter.

The risks of the trial drugs to the unborn child are currently unknown, so we would like to follow any pregnancies that occur to yourself or partner, during trial treatment or up to three (3) months after you complete trial treatment, up until the birth. This is so that we can learn more about the safety of the trial treatment.

If you or your partner becomes pregnant during the trial or within three (3) months of stopping treatment, you should inform your trial team immediately. Your trial team will discuss all the options available to you. The outcome and progress of any pregnancy will be followed by your trial team, (with your/your partner's consent), and we will ask you questions about the pregnancy and baby, if appropriate.

For Women of Child Bearing Potential: At the bottom of this document is an optional consent section. This is so that you can consent to the collection of data relating to your pregnancy, should a pregnancy occur.

For Male Participants: Should a pregnancy occur to your partner, your partner will be approached separately with information regarding the treatments you have received, and asked whether they would be willing to give consent for their pregnancy to be followed up.

<u>Private insurance policies</u>
You should discuss your participation in this trial with any insurance provider you have (e.g., protection insurance, life insurance, income protection, critical illness cover and private medical insurance) and seek advice if necessary, as failure to notify them may affect or invalidate your cover.

11. What are the side effects of the drug being tested?

Each person's reaction to a new drug is different. Some people have very few side-effects, while others may experience several. You will be closely monitored throughout the trial, but you should inform the trial team if you feel unwell or different in any way. If you have any major concerns or are feeling very unwell please contact your trial doctor immediately using the contact numbers at the end of this information sheet. Informing the trial team about any side-effects will help them manage your care, and they may be able to prescribe medication to ease any side effects. We also need to know so that the potential side-effects of this new combination can be recorded. A break from treatment may be necessary if you are experiencing side effects.

Side Effect	***Olaparib***	***Durvalumab***	**This table displays the side effects noted by the drug or**
Abdominal Pain	**Very Common**	**Very Common**	
Blood Result Abnormalities	**Very Common**	**Very Common**	

Bruising and/or pain from injection site		**Very Common**	**drugs you will receive.**<br><br>**Very Common** side effects typically affect more than 1 in 10 people<br><br>**Common** side effects typically affect less than 1 in 10 people<br><br>**<u>Combinations of drugs</u>**<br>Some data has been collected for combinations of drugs that will be taken in this trial. Some side effects were found to be more common when two of the drugs were taken together, than when taken on their own. Such side effects are indicated with an asterisk '*'
Cough	**Very Common**	**Very Common**	
Decreased Appetite	**Very Common**	**Very Common**	
Diarrhoea*	**Very Common***	**Very Common**	
Digestive problems	**Very Common**	Common	
Dizziness and/or Sickness	**Very Common**	**Very Common**	
Flu- like symptoms		Common	
Headache	**Very Common**		
High Temperature		**Very Common**	
Hoarse voice		Common	
Skin problems (itching, rashes)	Common	**Very Common**	
Lung Infections		**Very Common**	
Kidney function changes	Common	Common	
Mouth complications (dry or sore)	Common	Common	
Night Sweats		Common	
Pancreatic Problems		Common	
Shortness of breath	**Very Common**	**Very Common**	
Thyroid Function Changes		Common	
Tiredness and/or Weakness*	**Very Common***	**Very Common**	
Urinary Complications (pain or bleeding)		Common	
Water Retention (in limbs)		**Very Common**	

<u>Uncommon side effects</u>
In rarer instances (fewer than 1 in 100 people), other symptoms of taking these drugs have been noted that you should keep in mind before you agree to participate in the WIRE trial. Below these rare side effects are listed for the drug that you may be allocated, should you take part;

<u>Olaparib</u>
- Hypersensitivity reaction
- Itching and redness of the skin with or without a rash (dermatitis/eczema)
- Increase in volume of red blood cells (mean cell volume (MCV) elevation)

<u>Durvalumab</u>
- Long lasting autoimmune disease e.g.:
  - o Immune related dermatitis (skin rash)
  - o Thyroid problems
  - o Underactivity of the adrenal glands (adrenal insufficiency)
  - Diabetes Mellitus Type 1 (where insulin is required)
  - Inflammation of the Pituitary Gland (hypophysitis)

Section 2: Conduct of the trial

12. What if new information becomes available?

Sometimes during the course of a trial, new information becomes available which might affect your decision to continue participating in this trial. Your trial team will contact you to

discuss the new information and whether you wish to continue participating in the trial. If you still wish to continue on the trial, we will ask you to sign a new Informed Consent Form.

The trial sponsor, the regulatory authority or the trial doctor may decide to stop the trial at any time. If that happens we will tell you why the trial has been stopped and arrange for appropriate care and treatment for you.

13. What if I decide I no longer wish to participate in the trial?

You can stop participating in this trial at any time without giving a reason and without affecting your future care or medical treatment. If you decide not to participate any further, you will no longer receive the trial drugs. No further tests will be performed on you and no further research samples will be collected. Any data or results already collected will continue to be used in the trial analysis. Your trial team may also choose to withdraw you from the trial if they feel it is in your best interests or if you have been unable to comply with the requirements of the trial. Possible reasons for withdrawing you could include:

- Not taking the trial drugs as required
- Experiencing a serious side effect (your doctor will follow-up regarding your progress until this side effect has stabilised or resolved)
- Your disease gets worse whilst receiving trial treatment
- Finding out that you are not eligible after completing the screening tests
- Being unable to complete the visits or trial documentation as required
- Becoming pregnant or planning to become pregnant
- The trial doctor feels you no longer appear to benefit from the trial drugs.

14. What if there is a problem?

Any complaint about the way you have been dealt with during the trial or any possible harm you might suffer would of course be listened to and addressed wherever appropriate. If you have any concerns about any aspect of this trial you should speak to your trial doctor who will do their best to answer your questions.

In the event that something does go wrong and you are harmed by taking part in the research and this is due to someone's negligence then you may have grounds for a legal action for compensation against Cambridge University Hospitals NHS Foundation Trust or the University of Cambridge. The normal National Health Service complaints mechanisms will still be available to you (if appropriate). The University has obtained insurance which provides no-fault compensation, i.e., for non-negligent harm, you may be entitled to make a claim for this.

If you wish to complain or have any concerns about any aspect of the way you have been approached or treated during this trial, you can do this through the NHS complaints procedure. In the first instance it may be helpful to contact the (to be completed locally as appropriate – in England this will refer to the Patient Advice and Liaison Service (PALS)) at your hospital.

15. Will my taking part in this trial be kept confidential?

Cambridge University Hospitals NHS Foundation Trust (CUH) and The University of Cambridge are the Sponsors for this clinical trial based in the United Kingdom. They will be using information from you and your medical records in order to undertake this trial and will act as the data controller for this trial. This means that they are responsible for looking after your information and using it properly. The Sponsor organisations will keep identifiable

information about you for 5 years after the trial has finished to ensure your safety and allow the trial to be reviewed by the authorities after it is finished.

Your rights to access, to change or to move your information are limited, as the Sponsor organisations need to manage your information in specific ways in order for the research to be reliable and accurate. To safeguard your rights, we will use the minimum personally-identifiable information possible.

You can find out more about how the Sponsor (s) use(s) your information using the information below:

- For Cambridge University Hospitals NHS Foundation Trust, please visit: https://www.cuh.nhs.uk/corporate-information/about-us/our-responsibilities/looking-after-your-information

Or email the Data Protection Officer at: gdpr.enquiries@addenbrookes.nhs.uk

- For University of Cambridge, please visit:
https://www.medschl.cam.ac.uk/research/information-governance
Or email the Information Governance team at: researchgovernance@medschl.cam.ac.uk

For participants recruited at CUH (where the Sponsor is also the site):
Cambridge University Hospitals will collect your name, NHS number and contact details to contact you about this trial and make sure that relevant information about the trial is recorded for your care and to oversee the quality of the trial. Individuals from the Sponsors and regulatory organisations may look at your medical and research records to check the accuracy of this trial. Cambridge University Hospitals will pass these details to the Sponsors along with the information collected from you and/or your medical records. The only people in the Sponsor organisations who will have access to information that identifies you will be people who need to contact you in relation to this trial and to audit the data collection process. Cambridge University Hospitals will keep identifiable information about you from this trial for *5* years after the trial has finished.

For participants recruited at other participating sites:
(Add site name) will keep your name, NHS number and contact details to contact you about this trial, and make sure that relevant information about the trial is recorded for your care, and to oversee the quality of the trial. Certain individuals from the Sponsors, and regulatory organisations may look at your medical and research records to check the accuracy of this trial. The Sponsor(s) will only receive information without any identifying information. **(Add site name)** will keep identifiable information about you from this trial for *XX* years after the trial has finished.

All information collected about you as a result of your participation in the trial will be kept strictly confidential. Your personal and medical information will be kept in a secured file and be treated in the strictest confidence.

Once you have agreed to participate in this trial you will be allocated a Trial ID Number. This is a unique trial number which will be used on all your trial documentation along with your date of birth. Your date of birth is considered to be personal information. We collect this personal information on trial documentation to help ensure that the data we receive as part

of your trial participation is correctly allocated to you. By cross checking these two unique references we can ensure the integrity of the data.

When you agree to take part in this trial, the information about your health and care may be provided to researchers running other research studies in this organisation and in other organisations. These organisations may be universities, NHS organisations or companies involved in health and care research. Any serious side effects that occur during the trial will also be shared to the manufacturer of the trial drugs (AstraZeneca) using your trial-specific identifiers. The statistics for this trial is being undertaken by the University of Newcastle. All data that is shared with the University of Newcastle will be identified using only your trial ID and date of birth.

Your information will only be used for the purpose of health and care research, and cannot be used to contact you or to affect your care. It will not be used to make decisions about future services available to you, such as insurance.

Your coded trial data may be sent to other country(ies) outside the European Economic Area (EEA) for analyses, where the data protection laws are not the same. Your coded personal data may also be shared with off-site licenced laboratories for the purpose of analysing samples collected from you in this trial. However this information will not identify you and will not be combined with other information in a way that could identify you. The people who analyse the information will not be able to identify you and will not be able to find out your name, NHS number or contact details. Only anonymous trial data, without any personal information will be published at the end of the trial.
We will need to inform your GP of your participation in this trial. This is so that any medical decisions that your GP makes will take into account the fact that you are receiving drugs as part of this trial.

16. What will happen to my samples and scan images?

With your permission, we will analyse the blood, urine, kidney and tumour samples collected from you during this trial. This analysis will take place over a long period of time, so any results obtained will not alter your care pathway.

If you decide to stop taking part in the trial please tell the trial doctor if you want to change your mind about us using your donated sample(s) to look at the molecular and genetic makeup of the cancer.

Routine blood samples

Blood samples taken will be analysed immediately by your hospital and will be destroyed once the laboratory tests are complete. The results will include those you would receive as part of your standard care pathway, plus any results required for the trial. These results will be accessible to your trial team.

Research samples

Research samples taken will be labelled with your Trial ID Number, processed and stored initially by your trial team at your hospital, before being transferred to an approved facility for analyses. The approved facilities are either part of the Cambridge Biomedical Campus or AstraZeneca. These labs may be inside or outside of the UK. The samples will be securely stored and analysed by specialist teams. You will not be identifiable to researchers from any of the laboratories we may use for research analysis.

These samples will be analysed in a way which will tell us whether WIRE trial treatments are eliciting the biological response that we hope and expect to see. We will be for instance, assessing the tumour for a specific type of cell (CD8 positive T cells) that is indicative of your body fighting the cancer, which we believe should be more prevalent should you receive the Durvalumab drug. There may be other tests that we perform on your research samples to give us additional information regarding the drug activity.

Once the research analysis is complete any remaining samples will either be:

- Stored by the research team in line with the relevant regulatory requirements, in an appropriate facility pending ethical approval for use in a future trial outside of the WIRE trial.
- Will be disposed of in accordance with the UK Human Tissue Authority code of practice.

Archival tumour

You may have previously had a biopsy of your kidney, and therefore have more than one tumour sample. Not all patients will have undergone a previous biopsy. If you have however, we will ask your permission to access this 'Archival' tissue from your local hospital, so that we can compare the changes with your recent biopsy sample. This will also help us in the event that your most recent sample isn't big enough for us to perform our analysis. Granting us permission to access these samples is entirely optional.

Genetic Testing (All samples)

All of our cells in our body get information on how to work and function through a molecule called deoxyribonucleic acid (DNA). DNA is a record of instructions where the instructions are called genes. Genetic testing looks at your genes and could either look at one or some specific genes or all of your genes – your whole DNA. Genes affect how we grow and develop. Nobody else has exactly the same genes as you do, unless you have an identical twin. These differences mean that some people are more likely than others to get certain diseases and they also mean that medicines affect people differently. Some genes can be important in more than one disease.

Genetic testing in this trial is only for research purposes, but is an essential part of the trial. If you do not want to agree it to, you will be unable to take part. These genetic tests will be performed on any samples you donate to the trial, and requires no additional time from you.

The trial team may also undertake laboratory tests using your samples that reenact the environment of natural body conditions in a laboratory setting - so called '*ex vivo* modelling'.

This method of analysis typically provides very meaningful data regarding how cells interact naturally with the drugs received. Tissue cells are grown in a laboratory environment and could be modified to implant in mice with low resistance to disease. The laboratory staff will monitor the growth of the tumour (derived from your own) within the mice. Any laboratory performing this analysis would have the appropriate license for testing on mice. You have the right to take part in the trial without these particular lab procedures taking place. If you give your optional consent for these procedures, you may withdraw your consent for your samples to be used in this way at any point until this laboratory analysis begins.

Cambridge University Hospitals NHS Foundation Trust & the University of Cambridge may do this testing in its own laboratories or it may be done in the laboratories of other organisations (such as universities, collaborators and genetic specialist), or both. Results from this research may be shared and used as described in 'Will my taking part in this trial remain confidential?' In addition Cambridge University Hospitals NHS Foundation Trust &

the University of Cambridge, and its designated organisations may conduct future research where they will share summary results (not your individual results) or anonymous datasets with other researchers, from other companies or universities, for example. The results may be combined with the results of other studies, for example as part of scientific databases. The researchers can only use this information for medical and healthcare related research.

Radiological (Scan) Images

The images obtained by way of CT and/or MRI scans will be coded, using only your trial ID number and Date of Birth. These coded images will then be sent on a secure CD, through the post, to the Sponsors (Cambridge University Hospitals and the University of Cambridge). These images will be stored on password-protected computers at Cambridge University for the duration of the trial.

CT scans will be used by your doctor to monitor general size changes to the tumour, and will likely be kept at your local hospital, but the central trial team may request them for checking of the measurements provided by your hospital. The people performing the analysis on your MRI images will not be able to identify you. They will be experienced Radiologists that will be able to identify areas of the cancer to get a general idea of how the cancer is behaving; so called ‘Regions of Interest’. By using specialist software, the Radiologists can measure the amount of blood flowing through the cancer. This analysis will be part of the assessment of your MRI scan performed before your treatment, and after your treatment for comparison.

The anonymised images may support future research projects, for example finding new ways of gaining even more insight into renal cancer. Therefore, we ask for your permission to use these images, which do not identify you personally, in other future research projects.

17. What will happen to the results of the trial?

The results of the trial will be anonymous and you will not be able to be identified from any of the data produced. When the results of this trial are available they may be published in peer reviewed medical journals and used for medical presentations and conferences. They will also be published on the EU Clinical Trials Register website, a central registry for all clinical trials conducted in the EU.

Anonymised datasets from the trial will be made available to the funder (AstraZeneca), and may also be made available to other researchers in line with national and international data transparency initiatives.

If you would like to obtain a copy of the published results please contact your trial doctor directly who will be able to arrange this for you.

18. Who is funding the trial?

The trial is being funded by AstraZeneca and the Cancer Research UK Cambridge Centre. AstraZeneca is also supporting this trial by providing the trial drugs free of charge.

The trial is being managed by the Cambridge Clinical Trials Unit – Cancer Theme. The Chief Investigator is Mr Grant Stewart who is a University Lecturer at the University of Cambridge and Honorary Consultant Urological Surgeon at CUH.

19. Who has reviewed this trial?

All research within the NHS is reviewed by an independent group of people called a Research Ethics Committee, to protect your interests. This trial has been reviewed and given favourable opinion by the (name of REC here). The Medicines and Healthcare Products

Regulatory Agency (MHRA) who are responsible for regulating medicines in the UK have also reviewed this trial.

20. Further information and contact details

If you have any questions concerning this trial please contact your:

Trial Doctor
Name: [INSERT your hospital contact details here]
Tel: [INSERT your hospital contact details here]
Research Nurse
Name: [INSERT your hospital contact details here]
Tel: [INSERT your hospital contact details here]

In the event of an emergency please contact:

24-hour contact details
Contact: [INSERT your hospital contact details here]
Tel: [INSERT your hospital contact details here]

Outside of an emergency,
You may wish to contact one of the following organisations that are independent of the hospital at which you are being treated:

CancerHelp UK is a registered charity providing information about all aspects of cancer. It can provide useful information on cancer treatments and medical research. You can contact their nurses on freephone 0808 800 4040. You can also access their web site at: www.cancerresearchuk.org

MACMILLAN Cancer Support is a registered charity providing information about all aspects of cancer. They have published several useful booklets on different types of cancer, chemotherapy, radiotherapy, and clinical trials in general. You can contact the nurses on freephone 0808 808 0000. You can also access their website at www.macmillan.org.uk

If you wish to complain or have any concerns about any aspect of the way you have been approached or treated during this trial, you can do this through the NHS complaints procedure. In the first instance it may be helpful to contact the Patient Advice and Liaison Service (PALS) at your hospital.

Patient Advice and Liaison Service (PALS) at:
Hospital Name: [INSERT your hospital PALS contact details here]
Tel: [INSERT your hospital PALS contact details here]

INFORMED CONSENT FORM

Trial Title: WIRE: WIndow-of-opportunity clinical trials platform for evaluation of novel treatments strategies in **RE**nal cell cancer.

TO BE PRINTED ON HEADED PAPER

UNIVERSITY OF CAMBRIDGE

Cambridge University Hospitals NHS
NHS Foundation Trust

Principal Investigator: [INSERT your PI name here]

Participant Number: __________ (please add once the participant is registered)

**If you agree with each sentence below, please initial the box**		**INITIALS**
1	I have read and understood the Group 5, Durvalumab & Olaparib combination, Participant Information Sheet version 3.0, dated 10th February 2020 for the above trial and I confirm that the trial procedures and information have been explained to me. I have had the opportunity to ask questions and I am satisfied with the answers and explanations provided.	
2	I understand that my participation in this trial is voluntary and that I am free to withdraw at any time, without giving a reason and without my medical care or legal rights being affected. I also understand that data or samples already collected will continue to be used in the trial analysis.	
3	I understand that personal information about me will be collected and used in accordance with this information sheet. This information will be kept in the strictest confidence and none of my personal data will be published.	
4	I understand that sections of my medical notes or information related directly to my participation in this trial may be looked at by responsible individuals from the sponsor, regulatory authorities, research personnel where it is relevant to my taking part in research and that they will keep my personal information confidential. I give permission for these individuals to have access to my records.	
5	I understand that my GP will be informed of my participation in this trial and sent details of the WIRE trial.	
6	I have read and understood the compensation arrangements for this trial as specified in the Participant Information Sheet.	
7	I understand that the team/doctors in charge of this trial may close the trial or stop my participation in it at any time without my consent.	
8	I have read and understood my responsibilities for the trial including using appropriate contraception as listed in section 6.	
9	I agree to give blood and urine for research related to this trial.	
10	I agree to give tumour and healthy tissue samples for research related to this trial	
11	I give my permission to allow my kidney tissue and research blood, urine & tumour samples to be sent to specialist teams on the Cambridge Biomedical Campus, or to laboratories contracted and approved by the Sponsor(s) for tests/analysis related to this trial. I understand that only the minimum information will be supplied and that I will not be identifiable to any of the researchers.	
12	I understand that the results from the analysis carried out on the research blood and tumour samples will not be fed back to me.	
13	I understand that the information collected about me will be used to support other research in the future, and may be shared anonymously with other academic and commercial researchers external to the project, within the UK and beyond	
14	I give my permission to allow for DNA testing of my samples	
		**INITIALS**

TO BE PRINTED ON HEADED PAPER

UNIVERSITY OF CAMBRIDGE

Cambridge University Hospitals NHS
NHS Foundation Trust

**OPTIONAL:** Please read each statement below and initial the relevant box		Yes	No
15	I give my permission to allow for any archival tumour tissue to be collected for tests related to this trial. I understand that only the minimum information will be supplied and that I will not be identifiable to any of the researchers.		
16	I give my consent for my tissue samples to be used to grow cell cultures or be implanted into mice		
17	I give my permission to allow any remaining research blood and tumour samples taken as part of this trial to be retained in an HTA licenced facility pending Ethical Approval for use in another project at the discretion of the Chief Investigator. I understand that these samples will be retained in an approved storage facility, that only the minimum information will be supplied and, that I will not be identifiable.		
18	I give my permission for my anonymised radiological images to be used as part of the data for future research studies		
**FOR WOMEN OF CHILDBEARING POTENTIAL ONLY** **OPTIONAL:** Please read each statement below and initial the relevant box		**INITIALS**	
		Yes	No
19	If I become pregnant during, or in the three (3) months after receiving the trial drugs, I agree to information being collected about me, my pregnancy and my baby.		
20	I understand that sections of my medical notes or information related directly to my pregnancy may be looked at by responsible individuals from the sponsor, regulatory authorities and research personnel where it is relevant to my taking part in research. I give permission for these individuals to have access to my records.		
21	I agree to give my pregnancy information voluntarily and understand that I am free to withdraw at any time without giving a reason and without my medical care or legal rights being affected. I understand that all data collected up to the withdrawal of consent will be kept confidential.		

I agree to participate in this trial:

**Name of participant**	**Signature**	**Date**

**Name of person taking consent**	**Signature**	**Date**

Time of consent (24hr clock): _______:_______

1 copy for the participant, 1 copy for the trial team, 1 copy to be retained for hospital records

CANCER RESEARCH UK | CAMBRIDGE CENTRE

UNIVERSITY OF CAMBRIDGE Cambridge University Hospitals NHS

CCTU Cambridge Clinical Trials Unit
